# Evaluation of the Pharmacophoric Role of the O–O Bond in Synthetic Antileishmanial Compounds: Comparison between 1,2-Dioxanes and Tetrahydropyrans

**DOI:** 10.1021/acs.jmedchem.0c01589

**Published:** 2020-10-22

**Authors:** Margherita Ortalli, Stefania Varani, Giorgia Cimato, Ruben Veronesi, Arianna Quintavalla, Marco Lombardo, Magda Monari, Claudio Trombini

**Affiliations:** † Unit of Clinical Microbiology, Regional Reference Centre for Microbiological Emergencies (CRREM), St. Orsola-Malpighi University Hospital, Via Massarenti 9, 40138 Bologna, Italy; ‡ Department of Experimental, Diagnostic and Specialty Medicine, Alma Mater Studiorum - University of Bologna, Via Massarenti 9, 40138 Bologna, Italy; § Department of Chemistry “G. Ciamician”, Alma Mater Studiorum - University of Bologna Via Selmi 2, 40126 Bologna, Italy; ∥ Centro Interuniversitario di Ricerca sulla Malaria (CIRM) - Italian Malaria Network (IMN), University of Milan, 20100 Milan, Italy

## Abstract

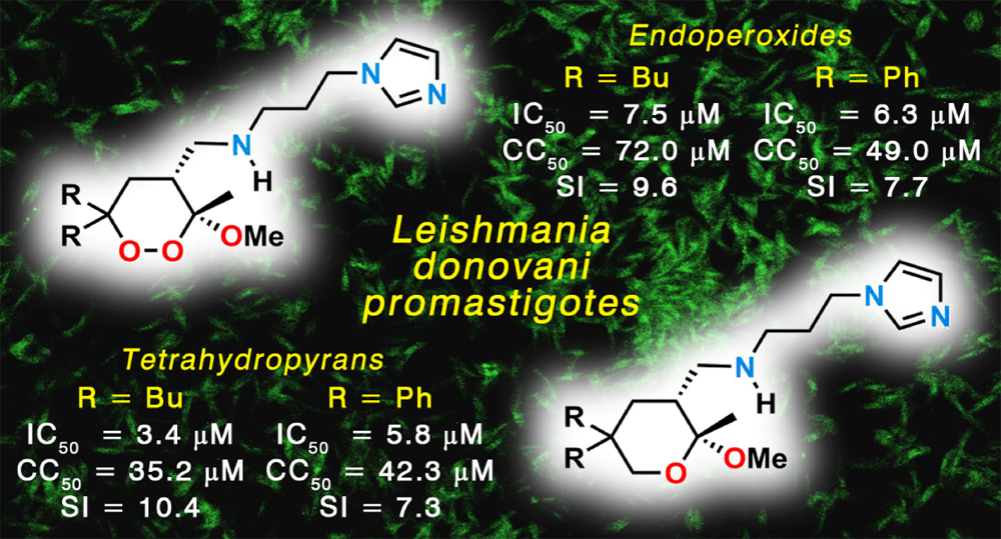

Leishmaniases are neglected diseases that can be treated with a limited drug arsenal; the development of new molecules is therefore a priority. Recent evidence indicates that endoperoxides, including artemisinin and its derivatives, possess antileishmanial activity. Here, 1,2-dioxanes were synthesized with their corresponding tetrahydropyrans lacking the peroxide bridge, to ascertain if this group is a key pharmacophoric requirement for the antileishmanial bioactivity. Newly synthesized compounds were examined *in vitro*, and their mechanism of action was preliminarily investigated. Three endoperoxides and their corresponding tetrahydropyrans effectively inhibited the growth of *Leishmania donovani* promastigotes and amastigotes, and iron did not play a significant role in their activation. Further, reactive oxygen species were produced in both endoperoxide- and tetrahydropyran-treated promastigotes. In conclusion, the peroxide group proved not to be crucial for the antileishmanial bioactivity of endoperoxides, under the tested conditions. Our findings reveal the potential of both 1,2-dioxanes and tetrahydropyrans as lead compounds for novel therapies against *Leishmania*.

## Introduction

**Figure 1:**
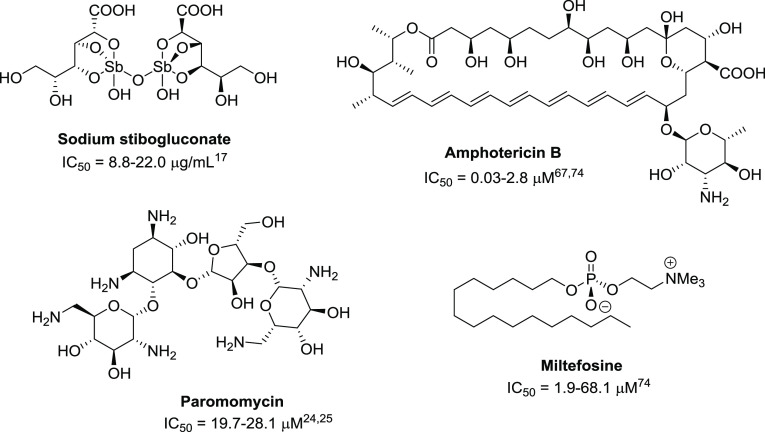
Drugs currently employed for the treatment of human leishmaniases (the reported IC_50_ values are relative to bioassays on promastigotes of *Leishmania donovani* strains).

*Leishmania* protozoa are endemic in most tropical and subtropical areas worldwide and in the Mediterranean Europe.^1−4^ Due to global warming and climate change, these parasites and their sand fly vectors are at risk to spread into countries previously considered nonendemic, including central and western Europe.^5−10^ The protozoa of the genus *Leishmania* are responsible for various forms of human leishmaniasis. While cutaneous leishmaniasis can be self-limiting, mucocutaneous infection can lead to profoundly disfiguring lesions, and visceral leishmaniasis is fatal if left untreated.^11^ Collectively, the parasites belonging to the genus *Leishmania* cause one of the most burdensome neglected tropical diseases, affecting predominantly poor populations, providing annually 1.5–2 million new cases and roughly 70,000 deaths.^12^ Currently, the pharmacological treatment for human leishmaniasis is based on few drugs^13^ (Figure 1), which lead to unsatisfactory results due to their toxicity, complex administration protocols (slow and painful intravenous infusion or intramuscular injection), variable effectiveness (depending on the disease clinical form or the infecting *Leishmania* species), and, more recently, growing drug resistance.^14−26^

**Figure 2:**
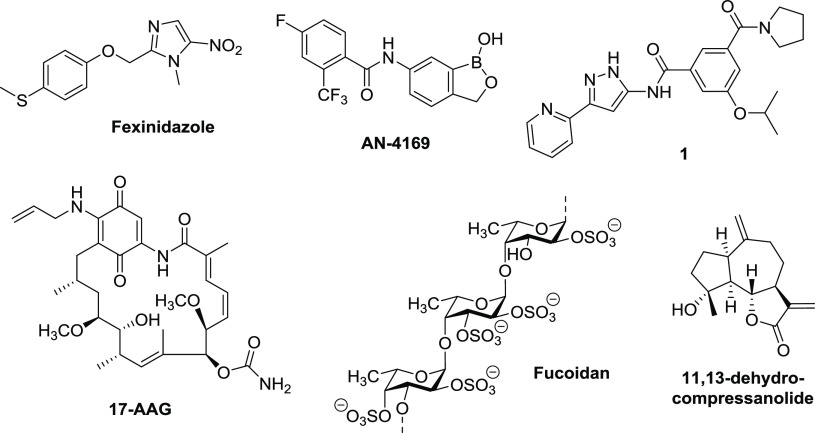
Some potential new leads for the treatment of leishmaniasis.

The early efforts to overcome these limitations were directed to the improvement of the drug delivery systems^27,28^ and the formulation of more effective combination therapies.^29^ However, the cost increase^30−32^ and the rapid recurrence of resistance phenomena^33−37^ prompted the scientific community to turn its attention toward the development of new antileishmanial drugs,^38−51^ which should be effective, safe, and not expensive. Concerning the research and development of new improved drugs, four main approaches are exploited: (i) drug repurposing, as in the case of fexinidazole^40^ (Figure 2); (ii) diversity-oriented screening of collections of chemicals, such as oxaborole **AN-4169**(39,52) and aminopyrazole amide **1**(39) (Figure 2); (iii) phenotypic screening, leading to the identification of new biological targets and effective inhibitors,^53−56^ such as **17-AAG** (HSP90 inhibitor)^57^ (Figure 2); and (iv) use of known or new natural products with limited side effects,^58−61^ especially those deriving from plants, such as fucoidan^58^ and 11,13-dehydrocompressanolide^61^ (Figure 2).

**Figure 3:**
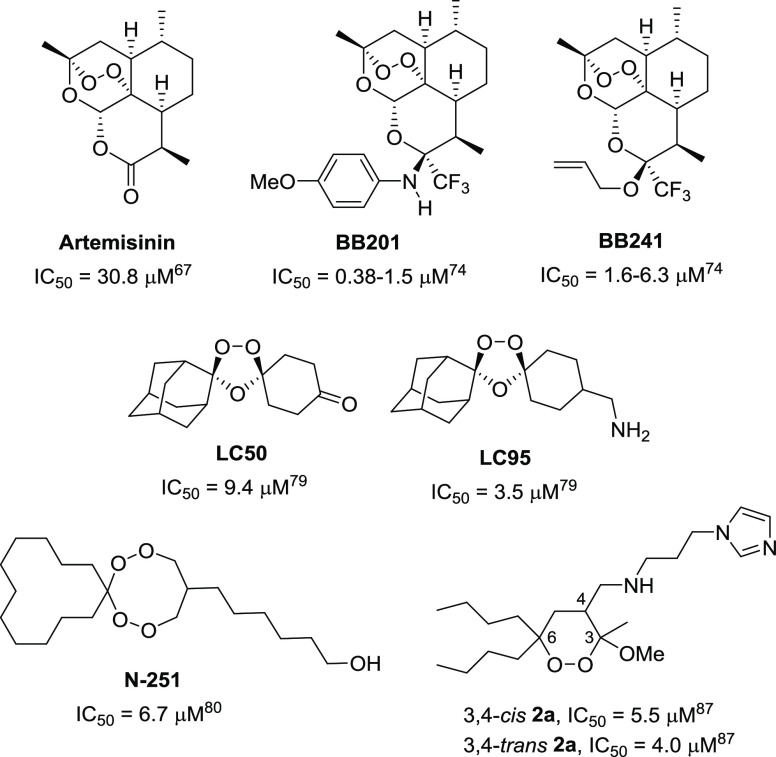
Antileishmanial activity of some recently studied natural and synthetic cyclic peroxides (IC_50_ values are relative to bioassays on promastigotes for all compounds, except for **N-251**, where the value is relative to amastigotes).^78^

Among the plant-derived antiparasitic compounds, some natural endoperoxides have shown a certain antileishmanial activity.^62−65^ In particular, artemisinin and some of its semisynthetic derivatives (Figure 3) have shown remarkable biological activities,^66,67^ especially as antimalarials; these compounds are currently recommended by World Health Organization as first-line treatment for *Plasmodium falciparum* malaria.^68,69^ Artemisinin and its derivatives also exhibited a promising bioactivity against other pathogenic protozoa, including *Trypanosoma* spp. and *Leishmania* spp.^66,67,70−76^ Focusing on the antileishmanial properties, it is worth mentioning that the *in vitro* potency of some semisynthetic artemisinin derivatives (**BB201** and **BB241**; Figure 3) is comparable to or higher than those of some currently employed drugs (amphotericin B and miltefosine).^66,67^ Moreover, artemisinin-derived endoperoxides are able to inhibit the parasite metabolism with limited adverse effects on the host cells.^66,77^

One of the limits to the development of drugs based on natural products is the low availability of the desired compound in the natural source, which renders the large-scale production difficult and expensive. For this reason, the total synthesis of structurally simple cyclic peroxides has been recently proposed. In 2015, Cortes and co-workers reported on the fully synthetic trioxolanes **LC50** and **LC95** (Figure 3), showing a promising bioactivity when tested *in vitro* against promastigotes (IC_50_ range, 3.5–9.4 μM) and intracellular amastigotes (IC_50_ range, 79.8–107.9 μM) of *L. infantum*.^79^ In 2019, Iwanaga *et al.* described the oral activity of the fully synthetic tetraoxaspiro **N-251** (Figure 3) against amastigotes of *L. donovani* (IC_50_ = 6.7 μM).^80^

**Figure 4:**
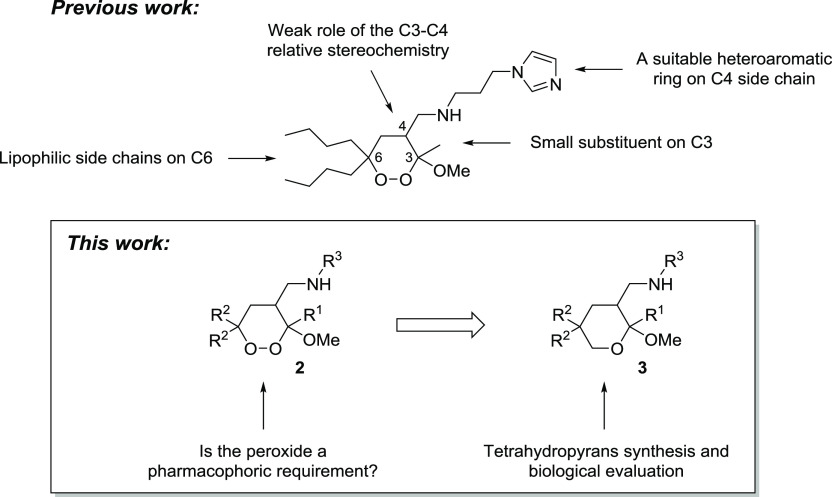
Previously identified pharmacophoric requirements of 3-methoxy-1,2-dioxanes **2** as antileishmanial agents^87^ and aim of the present work.

We recently proposed new structurally simple 3-methoxy-1,2-dioxanes **2** (Figure 3), obtained through an efficient and cheap synthetic approach.^81−87^ The simple and flexible protocol developed for their synthesis allowed us to collect a small library of 1,2-dioxanes **2**, variably substituted on C3, C4, and C6. When tested for *in vitro* antileishmanial activity on promastigotes of *L. donovani*, some of these compounds exhibited good inhibitory activities (IC_50_ range, 4.0–16.3 μM) and low toxicity (selectivity index range, 12.2–35).^87^ The most interesting compounds in terms of activity and selectivity were further tested *in vitro* on *L. donovani* amastigotes and *L. tropica*, *L. major*, and *L. infantum* promastigotes, showing good performance. A preliminary investigation of the structure–activity relationships (SARs) allowed us to identify some of the key pharmacophoric requirements for this class of antileishmanial endoperoxides (Figure 4): (i) lipophilic side chains must be present on C6; (ii) a long lipophilic side chain on C3 increases the cytotoxicity; (iii) the C3–C4 relative stereochemistry weakly affects the antileishmanial activity; and (iv) a heteroaromatic ring must be present on the C4 side chain and its nature significantly affects both activity and toxicity of 1,2-dioxane.

In the present study, we further investigate the structure–activity relationship of our synthetic 1,2-dioxanes, in order to establish whether the peroxide group is a crucial pharmacophoric requirement for the antileishmanial bioactivity (Figure 4). Concerning *P. falciparum* malaria, it has been demonstrated that the antimalarial peroxides act *via* intraerythrocytic activation of the O–O bond by Fe(II). The homolytic reductive decomposition of the peroxide function generates radical oxygen species, which trigger alkylation processes, leading to the parasite death.^88−100^ A pharmacophoric role of the peroxide bond in dioxanes has also been suggested against *Leishmania* parasites,^62,64,65,79,80,101^ but it has not yet entirely proved. Few studies investigated the mechanism of action of endoperoxides on *Leishmania* parasites, focusing only on natural scaffolds. In fact, Chatterjee *et al.* established that artemisinin exerts its activity on *L. donovani* promastigotes by an iron-dependent generation of free radicals.^102,103^ Gille and co-workers stated that the activation by Fe(II) of the peroxide bond in ascaridole and artemisinin is an essential part of their pharmacological mechanism *in vitro* on *L. tarentolae* promastigotes.^104^ Accordingly, the objectives of the present study are as follows: (i) the synthesis of tetrahydropyrans **3**, bearing the same substitution pattern of the already studied endoperoxides **2** and lacking the peroxide bridge (Figure 4); (ii) the evaluation of their antileishmanial properties to establish the role played by the O–O bond; and (iii) the preliminary investigation of the mechanism of action of the most promising bioactive compounds.

## Results

### Synthesis of Tetrahydropyrans **3**

**Scheme 1:**
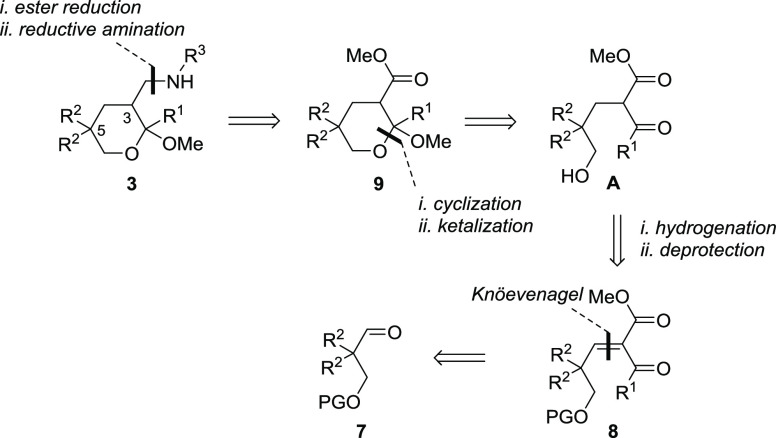
Retrosynthetic Analysis of Tetrahydropyrans **3** (PG = Protective Group)

Our retrosynthetic analysis for the construction of tetrahydropyrans **3** is sketched in Scheme 1. The amino side chain is installed on C3 exploiting the ester group of intermediate **9**, while the six-membered ring is built by a spontaneous intramolecular ketalization of intermediate **A**. The unsaturated β-ketoester **8** could in turn be obtained *via* a Knöevenagel condensation, starting from the protected 2,2-disubstituted 3-hydroxypropanal **7**.

**Scheme 2:**
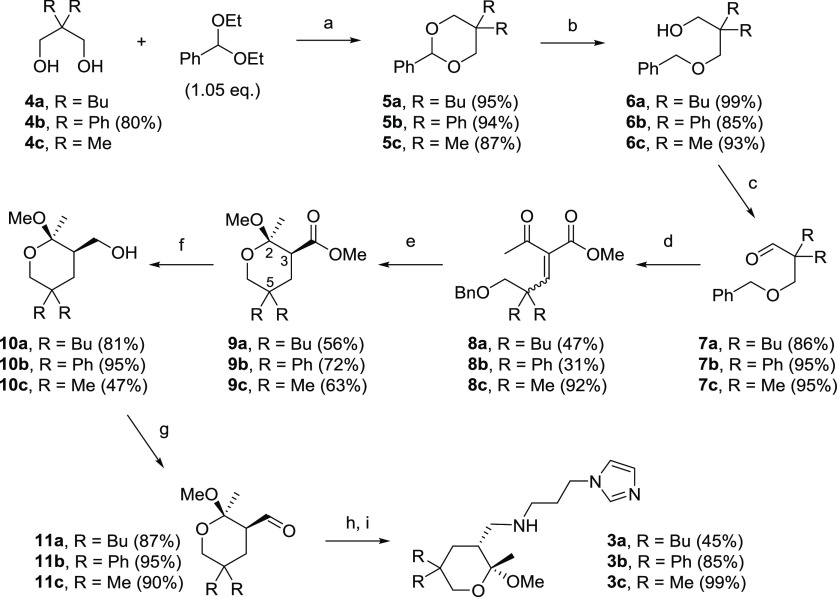
Synthetic Route Developed for the Construction of Tetrahydropyrans **3** (See Experimental Section for Details) Reagents and conditions: (a) CSA (1 mol %), DCM, 0 °C to rt, and 2–12 h; (b) DIBALH (2 equiv), DCM, −78 °C to rt, and 12 h; (c) Dess–Martin periodinane (1.05 equiv), DCM, 0 °C to rt, and overnight; (d) methyl acetoacetate (2 equiv), pyridine (4 equiv), TiCl_4_·2THF (2 equiv), THF, 0 °C to reflux, and 18 h; (e) Pd/C (20% w/w), H_2_ (filled balloon), MeOH, rt, and 12–16 h; (f) LiAlH_4_ (1 equiv), THF, 0 °C, and 1–3 h; (g) Dess–Martin periodinane (1.05 equiv), DCM, 0 °C to rt, and overnight; (h) 1-(3-aminopropyl)imidazole (1 equiv), MeOH, rt, and overnight; and (i) NaBH_4_ (1.5 equiv), 0 °C to rt, and 1 h.

Considering the good antileishmanial properties of C6-diphenyl and C6-dibutyl endoperoxides bearing the aminopropyl imidazole side chain on C4 (**2b** and **2a**, respectively; Table 1),^87^ we initially decided to prepare the analogous tetrahydropyrans **3b** (R^2^ = Ph and R^1^ = Me) and **3a** (R^2^ = Bu and R^1^ = Me) as well as the tetrahydropyran **3c** (R^2^ = R^1^ = Me), analogue of the inactive endoperoxide **2c** (Table 1), to confirm the pharmacophoric requirements of the lipophilic side chains on C6. The synthesis of **3b** (Scheme 2) started with the commercially available diphenylacetaldehyde, which was converted to the desired 2,2-disubstituted propanediol **4b** using a reported literature procedure.^105^ The protected aldehyde **7b** was thus obtained exploiting a sequence of three simple and high-yielding synthetic steps, consisting of a *trans*-acetalization (step a), a DIBALH-promoted acetal reduction (step b), and a Dess–Martin periodinane-promoted oxidation (step c).

**Table 1:**
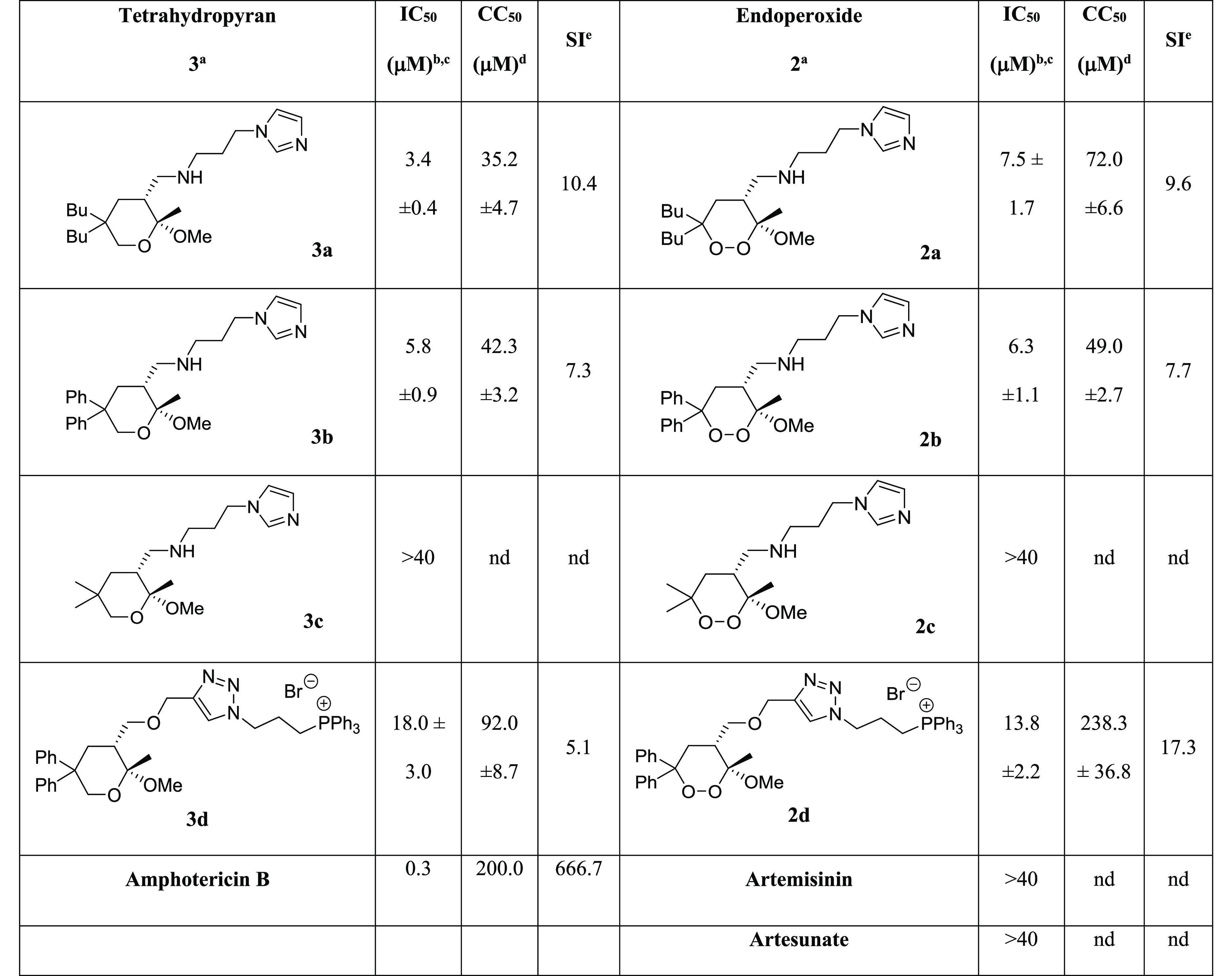
Inhibitory Activity of Tetrahydropyrans **3** and Endoperoxides **2** against Promastigotes of *L. donovani*, Cytotoxicity in Mammalian Kidney Epithelial Cells (Vero), and Selectivity Index (SI)

a Compounds tested as racemates.

b IC_50_ represents the concentration of a compound that causes 50% growth inhibition. Results represent the mean (± standard deviation, SD) of three independent experiments performed in duplicate.

c Additional susceptibility tests for selected compounds were performed on *L. donovani* cultures using 10% FBS in the culture medium, in order to compare the variation of IC_50_ with a less nutrient- and antioxidant-rich environment (see the Supporting Information, Table S2).

d CC_50_ represents 50% cytotoxic concentration on Vero cells. Results represent the mean (± standard deviation, SD) of three independent experiments performed in duplicate.

e Selectivity index (SI) = CC_50_/IC_50_; nd = not determined due to the low antileishmanial potency.

**Figure 5:**
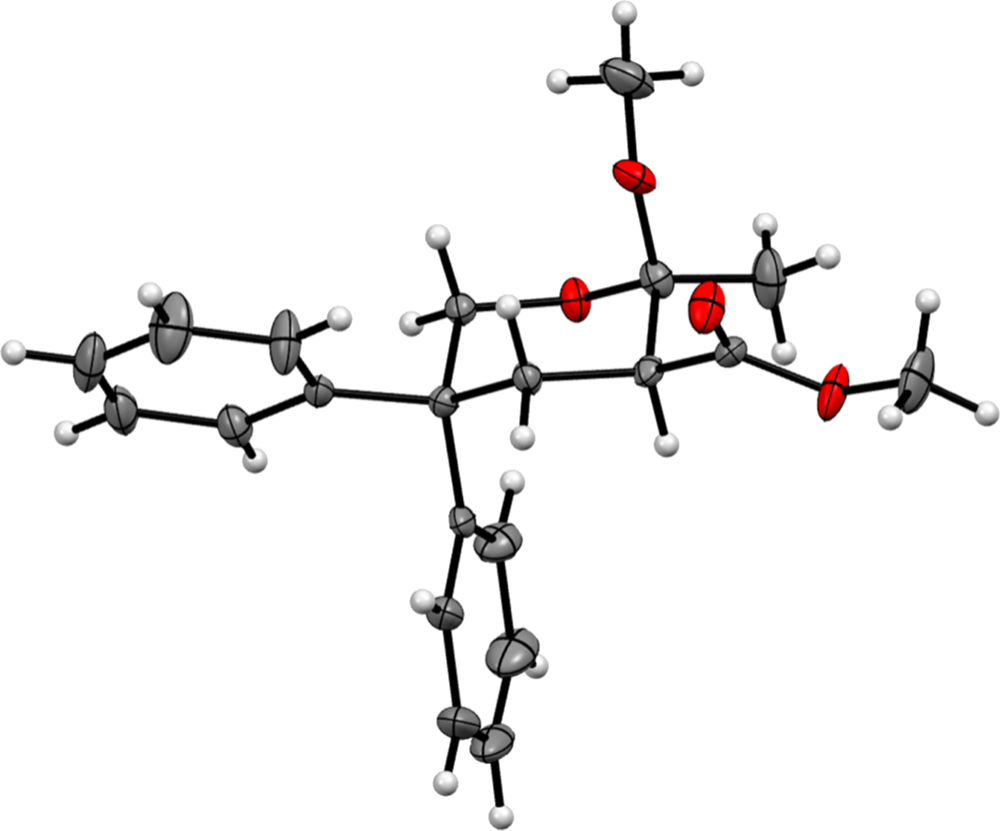
Determination of the relative stereochemistry of 2-methoxy tetrahydropyran **9b** through X-ray crystallographic analysis (thermal ellipsoids are drawn at 30% of the probability level).

The β-ketoester moiety was inserted on the intermediate aldehyde **7b***via* a Knöevenagel reaction. The high steric hindrance close to the reactive site of the α,α-disubstituted aldehyde and the generation of a congested trisubstituted olefin particularly hampered this process, which required harsh reaction conditions and excess of reagents.^106^ Despite the optimization attempts by varying the reaction temperature and reagent ratio, the yield of **8b** could not be significantly improved. A single stereoisomer of the diphenyl olefin **8b** was observed and isolated from the crude mixtures. The benzyl *O*-protecting group was selected in order to integrate in a single synthetic step the alcohol deprotection and the double bond hydrogenation, leading to the desired intermediate **A** (Scheme 1). Unexpectedly, 2-methoxy tetrahydropyran **9b** (Scheme 2) was directly obtained in good yield after the hydrogenation step, proving that four different transformations took place in a one-pot fashion: *O*-debenzylation, double bond reduction, cyclization, and methyl ketal formation.^107^ Moreover, this process was not only very efficient but also highly *cis*-stereoselective, as established by the X-ray crystallographic analysis of the isolated product **9b** (Figure 5, see the Supporting Information for details).^108^

At last, the replacement of the ester group on C3 with the aminopropyl imidazole side chain was accomplished following the protocol developed for the corresponding endoperoxides **2**,^87^ consisting in the reduction to an alcohol, followed by oxidation to the corresponding aldehyde and reductive amination (steps f–i, Scheme 2).

**Scheme 3:**
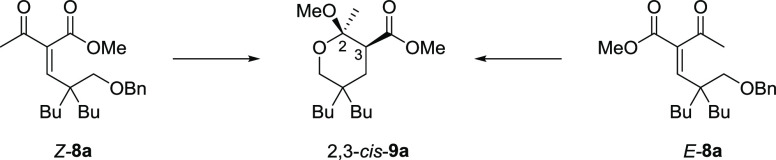
Reagents and Conditions: Pd/C (20% w/w), H_2_ (filled balloon), MeOH, rt, and Overnight

The synthetic approach proposed for tetrahydropyran **3b** was applied also to the construction of **3a** and **3c** (Scheme 2). The dibutyl (**4a**) and dimethyl (**4c**) diols are both commercially available, and they were efficiently converted into the corresponding α,α-disubstituted aldehydes **7a** and **7c**, respectively. The Knöevenagel reaction proceeded, as expected, with a moderate yield on the hindered α,α-dibutyl aldehyde **7a** but with an excellent yield on the α,α-dimethyl aldehyde **7c**, proving that the performance of this process is governed by the steric hindrance. In addition, it is worth mentioning that unlike the diphenyl derivative **8b** (Scheme 2), the dialkyl-substituted olefins **8a** and **8c** were obtained as mixtures of two isomers (**8a**: *Z*/*E* = 65:35, **8c**: *Z*/*E* = 60:40).^109^ To investigate the impact of the double bond geometry on the outcome of the following one-pot transformation, providing the tetrahydropyran system **9**, we carried out the reaction on the two isomers *Z*-**8a** and *E*-**8a**, separately (Scheme 3). Invariably, we obtained only the 2,3-*cis* tetrahydropyran **9a** from both olefins, demonstrating not only the *cis*-stereoconvergence^110^ proper of the process but also the comparable reaction rates for the two isomers. On this basis, the mixtures of *Z* and *E* isomers were directly used without separation.

The subsequent usual manipulations of the C3 side chain provided the desired aminopropyl imidazole tetrahydropyrans **3a** and **3c** (Scheme 2).

**Scheme 4:**
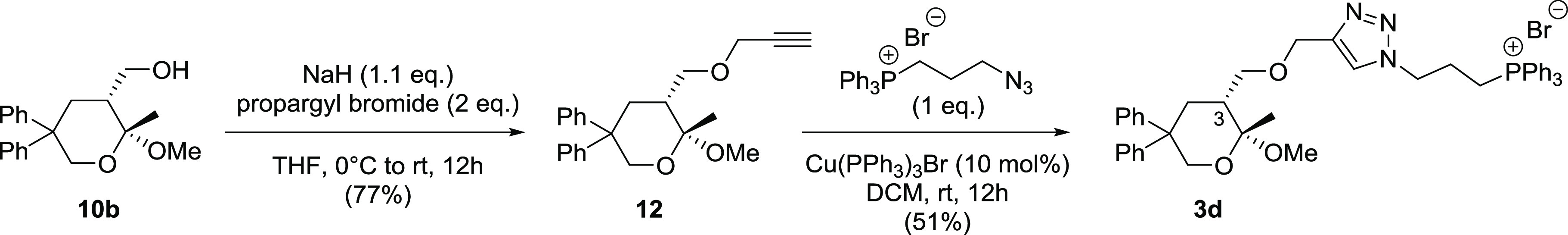
Synthesis of Tetrahydropyran **3d** Starting from Intermediate Alcohol **10b** (see Experimental Section for Details)

Finally, the intermediate alcohol **10b** (Scheme 2) was used as the starting material for the synthesis of the tetrahydropyran **3d**, analogue of the highly active antileishmanial endoperoxide **2d** (Table 1). This molecule, characterized by the presence of a triazole ring and a phosphonium salt in the C3 side chain, was prepared through an alkylation/click cycloaddition sequence developed for the construction of the corresponding endoperoxide **2d** (Scheme 4).^87^

### *In Vitro* Growth Inhibition of *L. donovani* Promastigotes and Amastigotes

The bioactivity of the new tetrahydropyrans **3** was initially evaluated against the extracellular promastigote forms of *L. donovani* (MHOM/NP/02/BPK282/0cl4) as the reference strain, indicative of the leishmaniasis in the Old World. The results were expressed as IC_50_, *i.e.*, the concentration of the product required to inhibit the parasite growth by 50%. When a compound could inhibit the promastigote growth, its cytotoxicity was also evaluated on Vero cells (Table 1) and on THP-1 cells (see the Supporting Information, Table S3) and the corresponding selectivity indexes (SIs) were calculated. The performance of each tetrahydropyran **3** was compared with that of the corresponding endoperoxide **2** (Table 1).

We observed a similar bioactivity profile for endoperoxides **2** and the corresponding tetrahydropyrans **3**; the aminopropyl imidazole derivatives **3a**/**2a** and **3b**/**2b** showed IC_50_ in the low micromolar range (Table 1). The parallel behavior was maintained also for methyl derivatives **3c** and **2c**, which were both inactive under our bioassay conditions (40 μM compound). The same set of tetrahydropyrans **3** and endoperoxides **2** was also tested on intramacrophage amastigote forms of *L. donovani* (Table 2).

**Table 2:**
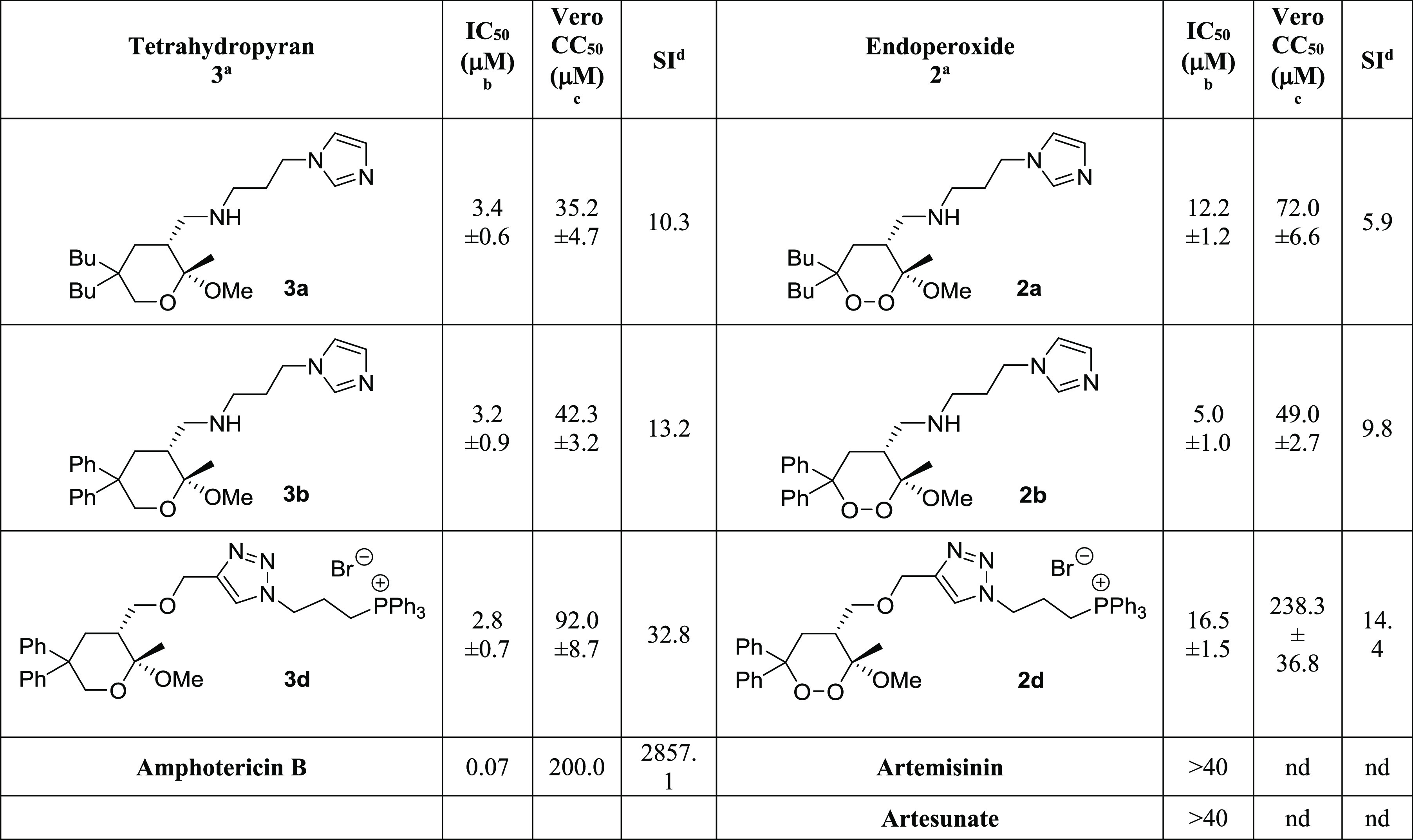
Inhibitory Activity of Tetrahydropyrans **3** and Endoperoxides **2** against Amastigotes of *L. donovani*, Cytotoxicity in Mammalian Kidney Epithelial Cells (Vero), and Selectivity Indexes (SIs)

a Compounds tested as racemates.

b IC_50_ represents the concentration of a compound that causes 50% growth inhibition. Results represent the mean (± standard deviation, SD) of three independent experiments performed in duplicate.

c CC_50_ represents 50% cytotoxic concentration on Vero cells. Results represent the mean (± standard deviation, SD) of three independent experiments performed in duplicate. Additional cytotoxicity tests were performed employing the THP-1 cell line (see the Supporting Information, Table S3).

d Selectivity index (SI) = CC_50_/IC_50_.

The good antileishmanial potency in the low micromolar range observed on promastigotes was preserved on amastigotes for both classes of compounds. Moreover, it is interesting to note that our synthetic structurally simple compounds, endoperoxides **2** and tetrahydropyrans **3**, are significantly more potent against *L. donovani* parasites (promastigote and amastigote forms) than artemisinin and artesunate (Tables 1 and 2).

In particular, tetrahydropyrans **3** revealed to be slightly more active against amastigotes than the corresponding endoperoxides **2**.

### Investigation on the Mechanism of Action of 1,2-Dioxanes **2** and Tetrahydropyrans **3** against *L. donovani*

#### Study on the Iron Role in the Activation of Synthetic Antileishmanial Compounds **2** and **3**

Little is known about the mechanism of action of endoperoxides on *Leishmania* parasites. To investigate whether iron plays a role in triggering the antileishmanial bioactivity of our synthetic compounds, we studied whether the inhibition caused by **2a** and **3a** on *L. donovani* promastigotes was influenced by the presence of an iron chelator (desferrioxamine, DFO);^111,112^ the results are presented in Table 3.

**Table 3:**
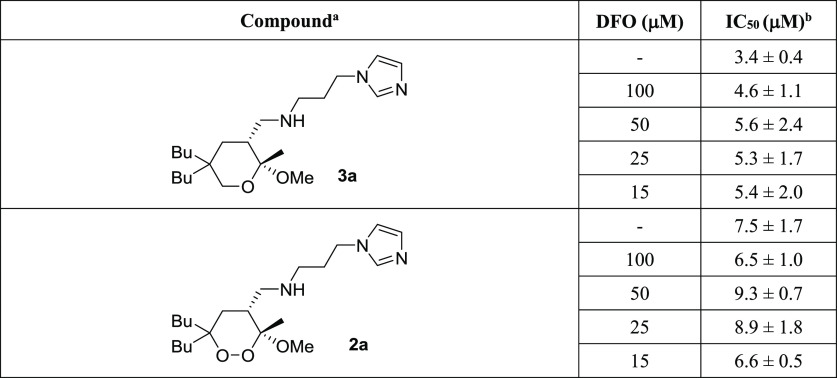
Inhibitory Activity of Tetrahydropyran **3a** and Endoperoxide **2a** against Promastigotes of *L. donovani* in the Presence or Absence of the Iron Chelator DFO

a Compounds tested as racemates.

b IC_50_ represents the concentration of a compound that causes 50% growth inhibition. Results represent the mean (± standard deviation, SD) of three independent experiments performed in duplicate. We also tested these compounds in the presence of deferiprone (DFP), which is a more lipophilic iron chelator than DFO (see the Supporting Information, Table S5 and Figure S3). DFO = desferrioxamine.

**Figure 6:**
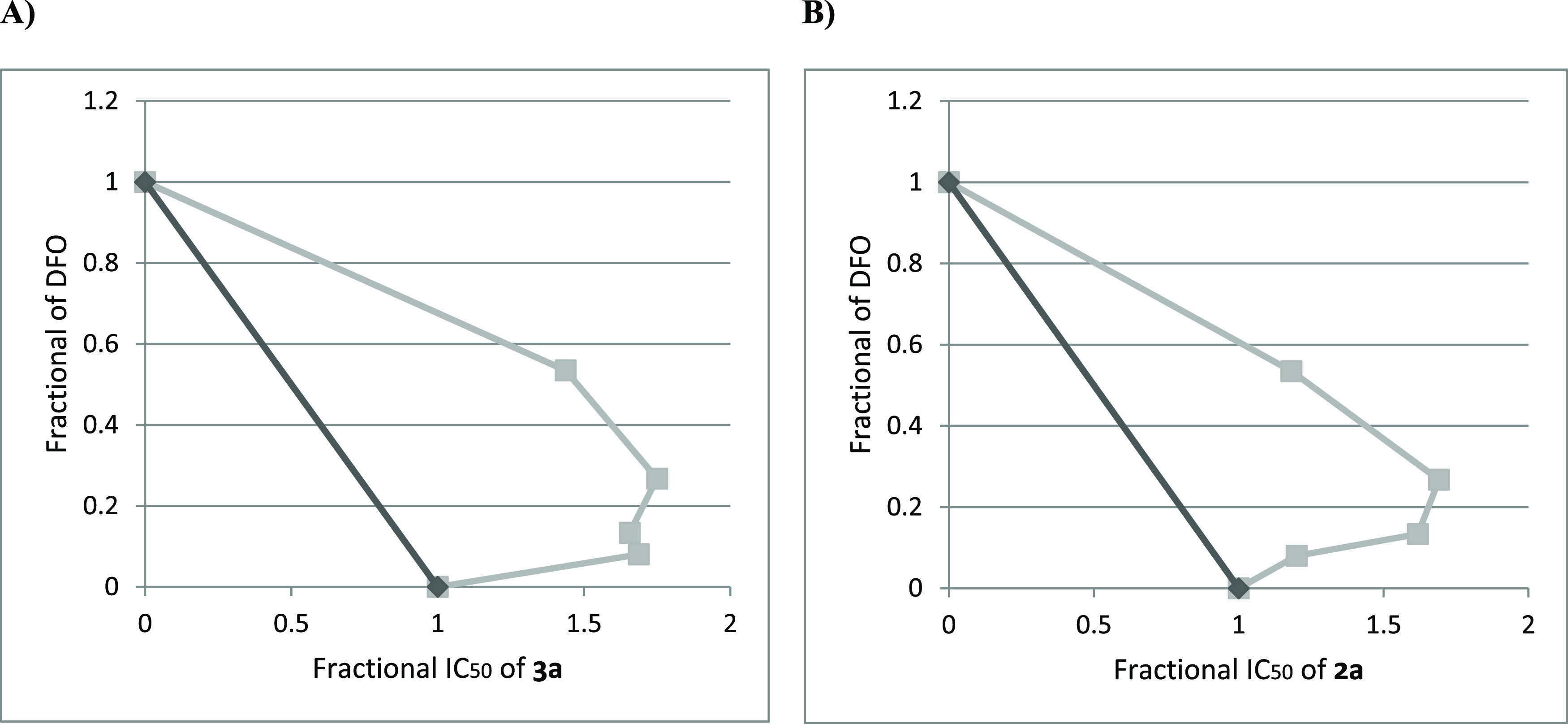
(A, B) Isobolograms depicting the interaction of (A) tetrahydropyran **3a** with the iron chelator DFO and (B) endoperoxide **2a** with the iron chelator DFO. A dark gray line is line of additivity. *X* axes depict the fractional inhibitory concentration (FIC = IC_50_ of the drug in the combination/IC_50_ of the drug when tested alone). *Y* axes depict the fractional of DFO. Square in the figure indicates ΣFIC values from each drug combination.

At first, we determined IC_50_ and CC_50_ values of the iron chelator DFO (187 and >400 μM, respectively). Then, the effect of the iron chelator presence on the bioactivity of the two compounds was studied by coincubating **2a** and **3a** with DFO at different concentrations. The variation of IC_50_ of the compounds in relation to the different doses of DFO was determined by the alamarBlue assay (Table 3), and the isobole technique was employed to depict the interactions of **2a** and **3a** with DFO (Figure 6).

As shown in Table 3, the inhibitory activity of **3a** and **2a** against *Leishmania* parasites did not undergo significant variation in the presence of various concentrations of DFO, proving a scarce influence of the iron chelator on the bioactivity of the compounds. The isobole technique was used for depicting synergistic or antagonistic interaction between the iron chelator and the two compounds on the parasite growth (Figure 6). Combinations are expressed as the sum of the fractional inhibitory concentrations (ΣFIC), and FIC values are defined as synergism (<0.5), antagonism (>4.0), and additivity (no interaction). The strength of synergism (or antagonism) is indicated by the degree of deviation from the line of additivity. As shown in Figure 6, combination of DFO with **2a** or **3a** resulted in additive effects as no antagonism or synergism was observed. These findings suggest that low-molecular-weight iron species do not play a crucial role in triggering the antileishmanial activity of the tested compounds.

The effect of the iron chelator DFO was investigated also on the pair of compounds **2b** and **3b**, confirming the same behavior observed for **2a** and **3a**, respectively (see the Supporting Information, Table S4 and Figure S2).

#### Reactive Oxygen Species (ROS) Production Induced by Antileishmanial Compounds **2** and **3** in *L. donovani* Promastigotes

We evaluated the ability of our synthetic compounds to generate reactive oxygen species (ROS) in *L. donovani* promastigotes. We selected the endoperoxide **2a** and the tetrahydropyran **3a** as model substrates, which were administered to the parasites in the presence of 2,7-dichlorodihydrofluorescein diacetate (H_2_DCFDA), a live-cell-permeable dye, which is oxidized by ROS, providing a fluorescent compound (DCF) (see Experimental Section for details).^103^ Therefore, the resultant fluorescence is a direct measure of the amount of ROS generated.

**Figure 7:**
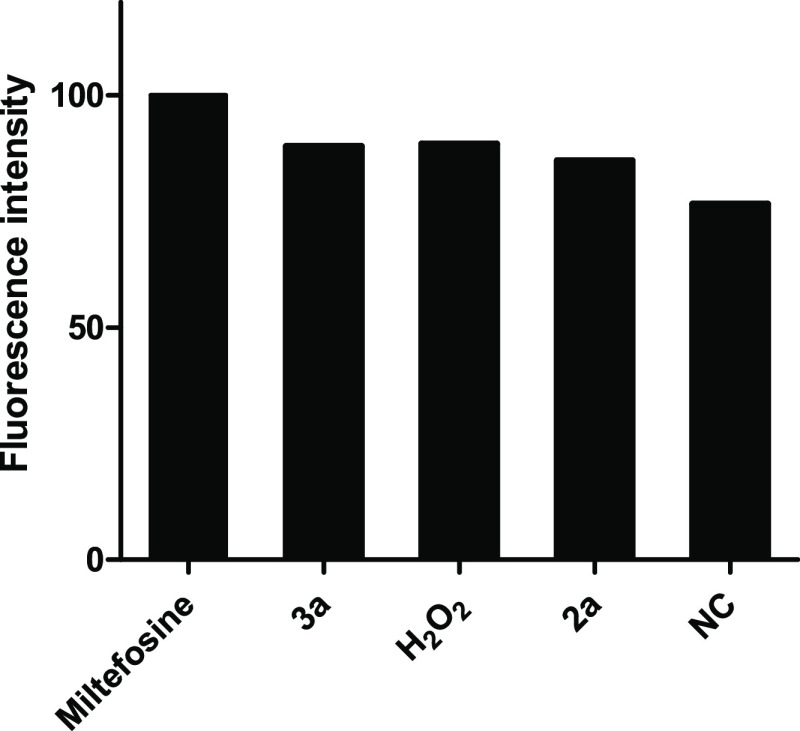
Measurement of ROS in promastigotes of *L. donovani* in the presence of endoperoxide **2a** and tetrahydropyran **3a** tested at their IC_50_. Generation of ROS was measured using 2,7-dichlorodihydrofluorescein diacetate (H_2_DCFDA). Miltefosine (22 μM) and H_2_O_2_ (20 μM) were used as positive controls. NC: negative control (untreated parasites). The parasites were analyzed by fluorimetry. Results represent the mean of three independent experiments performed in triplicate.

**Figure 8:**
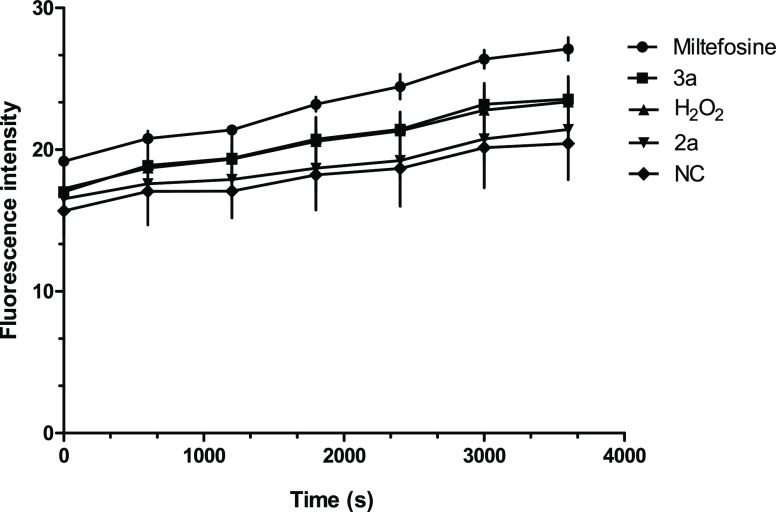
Quantification of ROS generated upon treatment of *L. donovani* promastigotes with tetrahydropyran **3a** (IC_50_), endoperoxide **2a** (IC_50_), miltefosine (22 μM), and H_2_O_2_ (20 μM) or without treatment (negative control, NC) for 1–60 min. Results represent the mean (± standard deviation, SD) of three independent experiments performed in triplicate.

As shown in Figure 7, endoperoxide **2a** and tetrahydropyran **3a** were both capable of inducing an increase in the intracellular ROS levels at their IC_50_ as compared to the negative control (untreated promastigotes). In details, **3a** showed a higher ROS production when compared to H_2_O_2_ (20 μM, positive control) but a slightly lower ROS production when compared to miltefosine (22 μM, positive control). Compound **2a** showed a lower fluorescence intensity when compared to H_2_O_2_ and miltefosine; however, a higher amount of ROS was detected in **2a**-treated promastigotes in comparison with untreated cells. Quantification of the generated ROS was monitored for 1–60 min. As shown in Figure 8, intracellular ROS levels increased over time in untreated and treated promastigotes, but the increase was higher in parasites that were treated with **3a** and **2a** than in untreated cells. These results indicate that **3a** and **2a** induced oxidative stress in *L. donovani* promastigotes, with ROS production induced by **3a** overlapping with the positive control H_2_O_2_ (Figure 8). The data obtained from the ROS levels evaluation led us to two main observations: (i) the tetrahydropyran **3a** induced a higher increase in intracellular ROS levels than the endoperoxide **2a**, and (ii) the amount of ROS generated by **3a** and **2a** was in accordance with the IC_50_ profile of the compounds as a higher inhibitory activity was found for **3a** (IC_50_ 3.4 μM) as compared to **2a** (IC_50_, 7.5 μM).

### Localization Studies of Tetrahydropyrans **3** and 1,2-Dioxanes **2** in Parasitic Cells

**Scheme 5:**
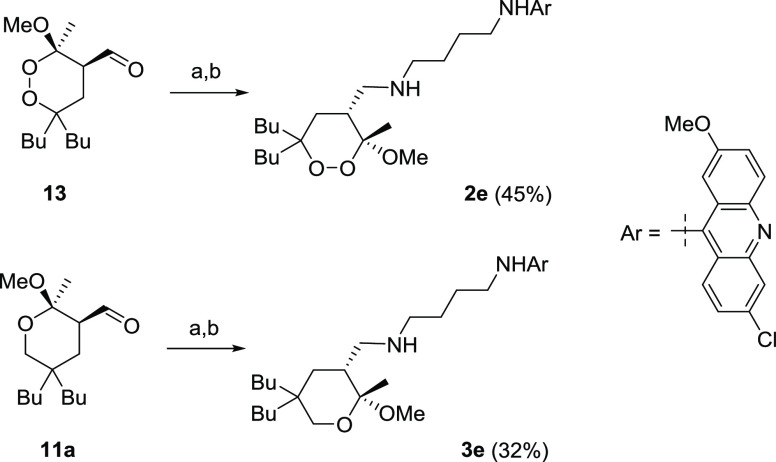
Reagents and Conditions: (a) *N*^1^-(6-Chloro-2-methoxyacridin-9-yl)butane-1,4-diamine (1 equiv), MeOH, rt, and Overnight and (b) NaBH_4_ (1.5 equiv), 0 °C to rt, and 1 h (see Experimental Section for Details) For the synthesis of **13**, see ref (87).

**Scheme 6:**
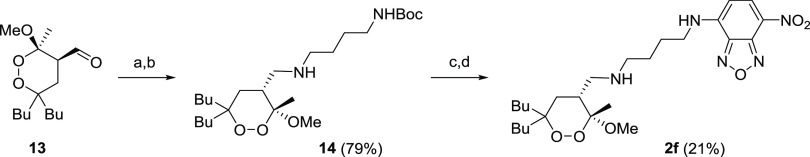
Reagents and Conditions: (a) *tert*-Butyl (4-Aminobutyl)carbamate (1 equiv), MeOH, rt, and Overnight; (b) NaBH_4_ (1.5 equiv), 0 °C to rt, and 1 h; (c) HCl in Et_2_O (1 M, 20 equiv), Et_2_O, rt, and Overnight; and (d) NBD-Cl (1.1 equiv), TEA (3 equiv), DCM, 0 °C to rt, and 4 h (see Experimental Section for Details) For the synthesis of **13**, see ref (87).

In order to acquire information on the ability of the tested compounds to enter the *L. donovani* promastigote cell and on their biodistribution within the parasite, we accomplished a confocal microscopy investigation. For this purpose, we synthesized new compounds carrying a fluorescent probe (Schemes 5 and 6). In particular, the most active butyl derivatives were labeled with an acridine-based fluorescent dye (**2e** and **3e**, Scheme 5), which was introduced exploiting the final reductive amination step of the previously developed synthetic route. The isolation yields of the fluorescent endoperoxide **2e** and tetrahydropyran **3e** were moderate due to their poor solubility in organic solvents, which made the purification difficult.

We were aware that the fluorescence of acridine-derived labels can be quenched under certain conditions (*e.g.*, acidic pH value, presence of hematin, etc.) and that this kind of fluorophore can easily bind to DNA, leading to altered results.^113^ To validate the biodistribution data obtained from acridine-containing compounds **2e** and **3e**, we synthesized also the endoperoxide **2f** (Scheme 6) bearing the nitrobenzyldiazole (NBD) fluorophore, to be used as a comparison since it does not have the abovementioned limitations. The construction of **2f** (Scheme 6) was difficult as the nitrobenzyldiazole system revealed to be not stable under reductive amination conditions. In particular, the introduction of the diamino side chain on C4 proceeded smoothly (**14**, 79% yield). Conversely, the *N*-Boc deprotection employing anhydrous HCl and the isolation of the corresponding product as the hydrochloride salt revealed to be critical (step c, Scheme 6). For this reason, the coupling with NBD-Cl was carried out on the crude intermediate. However, the isolated unoptimized yield of the fluorescent endoperoxide **2f** was modest.

At first, the bioactivity of the new fluorescent compounds was evaluated on promastigotes of *L. donovani* and the corresponding cytotoxicity on Vero cells was also assayed (Table 4). The acridin-containing derivatives **3e** and **2e** revealed to be more potent against *L. donovani* promastigotes than the corresponding imidazole-containing products **3a** and **2a**, respectively (Table 1). This effect could be due to a toxicity contribution of the acridin system, as suggested by the low selectivity index (SI) of these compounds, which were more toxic on Vero cells than the corresponding nonlabeled compounds. However, it is important to emphasize that the behavior of endoperoxide **2e** and tetrahydropyran **3e** remains comparable also for this set of labeled molecules.

**Table 4:**
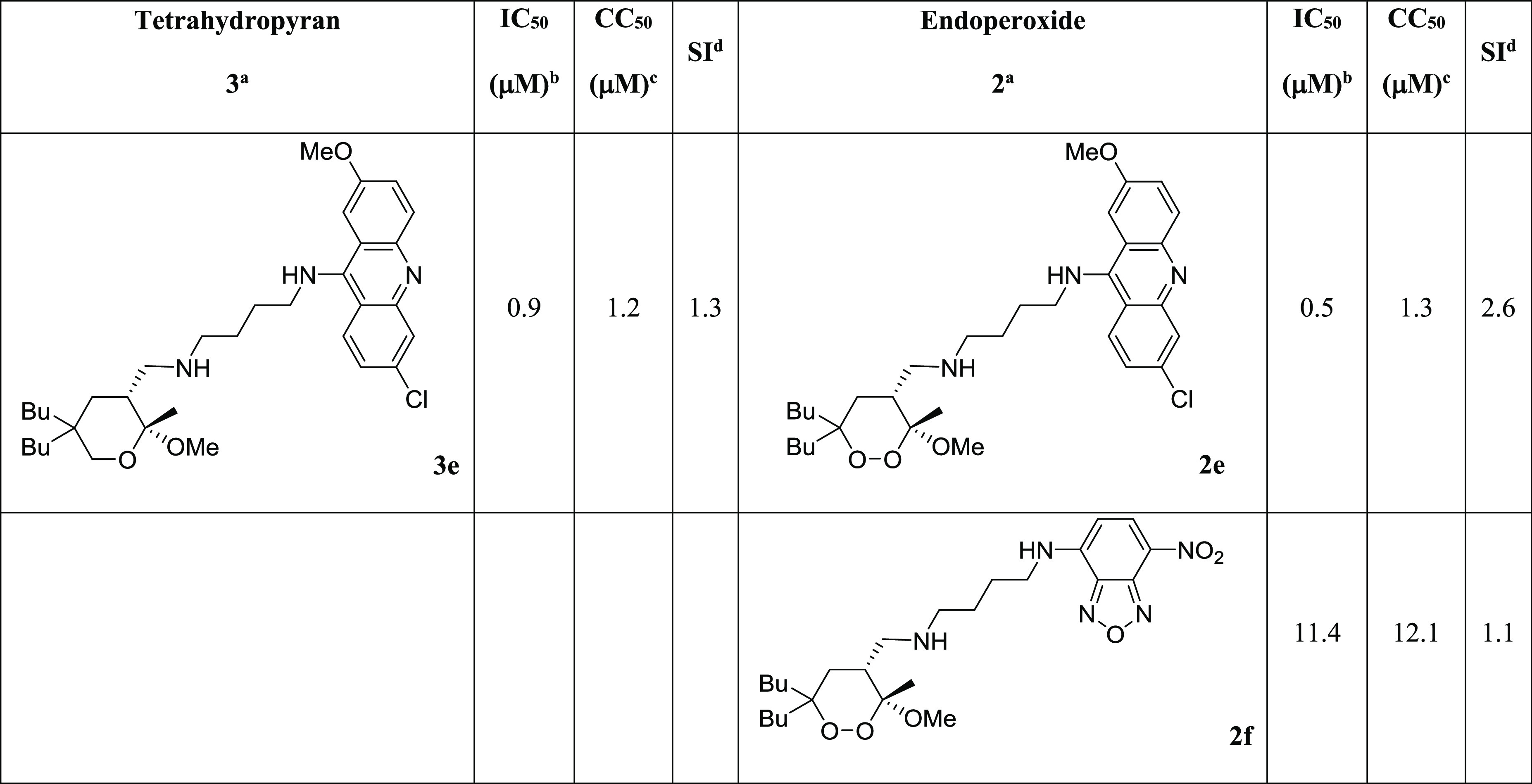
Inhibitory Activity of Fluorescent Compounds **2e**, **2f**, and **3e** against Promastigotes of *L. donovani*, Cytotoxicity in Mammalian Kidney Epithelial Cells (Vero), and Selectivity Index (SI)

a Compounds tested as racemates.

b IC_50_ represents the concentration of a compound that causes 50% growth inhibition.

c CC_50_ represents 50% cytotoxic concentration on Vero cells.

d Selectivity index (SI) = CC_50_/IC_50_.

The NBD derivative **2f** showed lower activity against *L. donovani* promastigotes than the corresponding acridin-derivative **2e** (Table 4), and its IC_50_ value was similar to that of the corresponding imidazole-containing compound **2a** (Table 1). However, its SI was low, confirming a significant toxicity of these fluorescent products on Vero cells.

**Figure 9:**
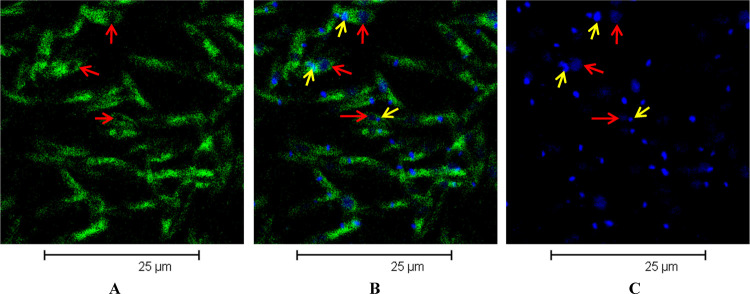
Cell localization of labeled endoperoxide **2f** and TO-PRO-3 in promastigotes of *L. donovani*. (A–C) Confocal microscopy images show (A) uptake of **2f** with green fluorescence, (B) overlay of uptake of **2f** with green fluorescence and uptake of TO-PRO-3 with blue fluorescence, and (C) uptake of TO-PRO-3, staining nuclei and kinetoplasts with blue fluorescence. Red arrows indicate nuclei, and yellow arrows indicate kinetoplasts.

The labeled compounds were incubated with *L. donovani* promastigotes, and then confocal microscopy images were acquired (see Experimental Section for details). Figure 9 shows data obtained with the NBD-labeled endoperoxide **2f**. We noticed a marked accumulation of the fluorescent probe in the parasite cytoplasm, whereas a dark area was evident inside the parasite (Figure 9A), likely corresponding to the nucleus. To confirm this interpretation, we marked the nucleus and kinetoplast of the parasite with TO-PRO-3 (Figure 9C), a specific dye for nucleic acids.^114,115^ The overlapped color image (Figure 9B) clearly shows the dark blue nucleus (red arrows, Figure 9) and the cyan kinetoplast (yellow arrows, Figure 9), whose color derived from a colocalization of green and blue fluorescence. This finding suggested that endoperoxide **2f** spread throughout the parasite cytoplasm and it was able to enter the kinetoplast but not the nucleus.

**Figure 10:**
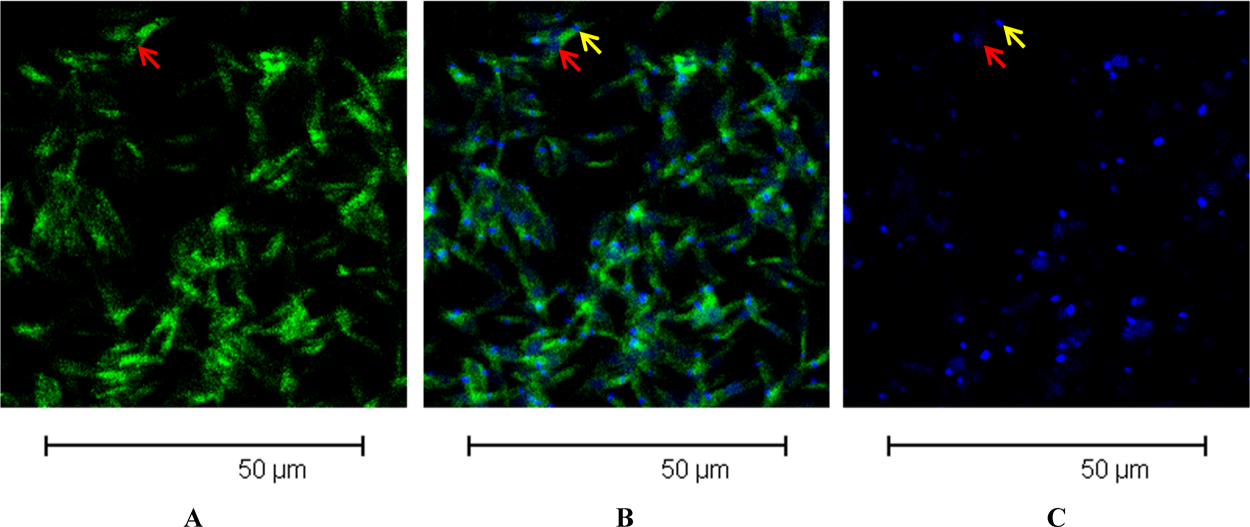
Cell localization of labeled tetrahydropyran **3e** and TO-PRO-3 in promastigotes of *L. donovani*. (A–C) Confocal microscopy images show (A) uptake of **3e** with green fluorescence, (B) overlay of uptake of **3e** with green fluorescence and uptake of TO-PRO-3 with blue fluorescence, and (C) uptake of TO-PRO-3 with blue fluorescence. Red arrows indicate nuclei, and yellow arrows indicate kinetoplasts.

A similar intraparasitic distribution was observed for acridine-labeled tetrahydropyran **3e** by confocal microscopy (Figure 10).

**Figure 11:**
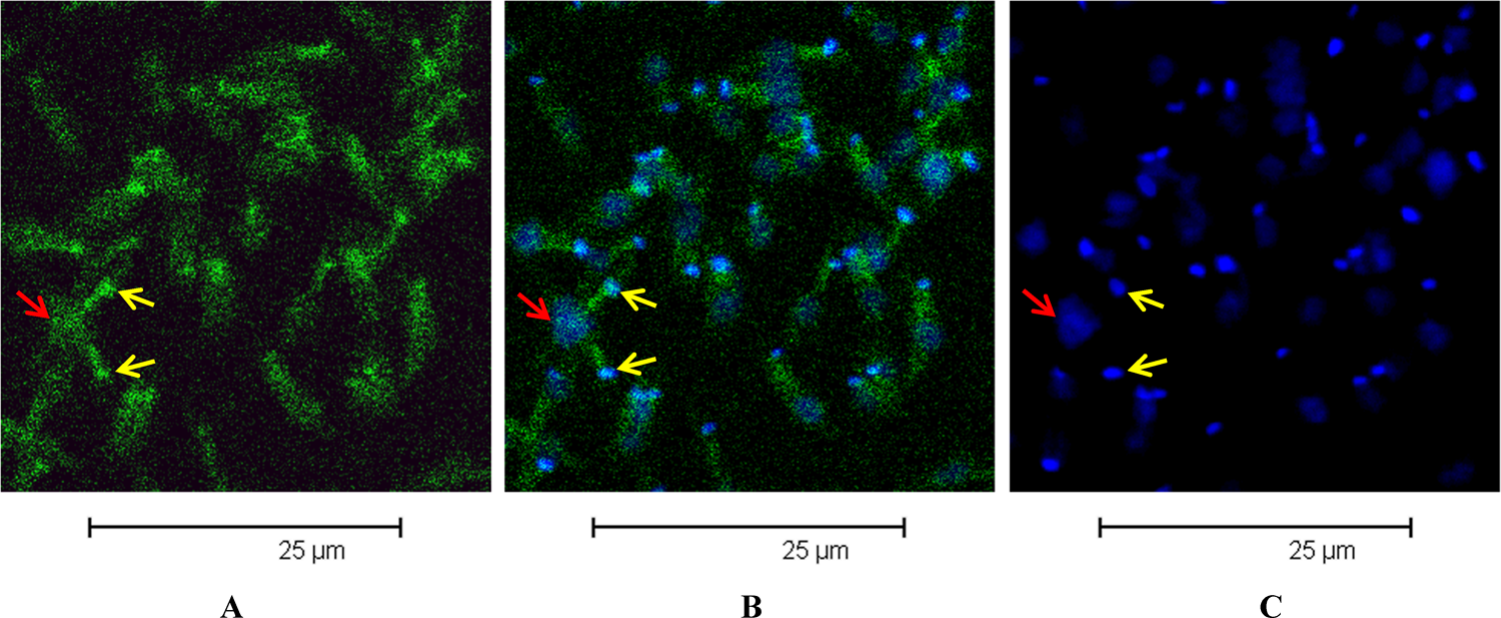
Cell localization of labeled endoperoxide **2e** and TO-PRO-3 in promastigotes of *L. donovani*. (A–C) Confocal microscopy images show (A) uptake of **2e** with green fluorescence, (B) overlay of uptake of **2e** with green fluorescence and uptake of TO-PRO-3 with blue fluorescence, and (C) uptake of TO-PRO-3 with blue fluorescence. Red arrows indicate nuclei, and yellow arrows indicate kinetoplasts.

Concerning acridine-labeled endoperoxide **2e**, we observed a colocalization of green and blue fluorescence not only in the kinetoplast but also in the nucleus (Figure 11B). This finding suggests that this peculiar acridine-containing compound can enter the nucleus core.

## Discussion and Conclusions

Little is known on the mechanism of action of endoperoxides against *Leishmania*. Previous mechanistic studies on natural endoperoxides, such as artemisinin, suggested that they act through an iron(II)-mediated homolytic cleavage of the peroxide function, leading to the formation of cytotoxic radicals.^102−104^ Based on these studies, we expected that endoperoxides **2** would exhibit higher antileishmanial activity than the corresponding tetrahydropyrans **3**, the latter lacking the O–O bond. Surprisingly, we observed a similar bioactivity profile for the two classes of compounds (Table 1) and tetrahydropyrans **3** revealed to be slightly more active against intracellular amastigotes than the corresponding endoperoxides **2** (Table 2). The latter finding is particularly meaningful taking into account that *Leishmania* amastigotes obtain the iron necessary for its metabolism from the host macrophage.^102,116^ Considering the greater iron availability for amastigotes than promastigotes, the compounds activated by iron are normally more active against amastigotes than promastigotes,^102^ while we observed an opposite behavior for our products. These findings suggest that in the tested conditions, the primary mechanism of action of endoperoxides **2** and tetrahydropyrans **3** on *L. donovani* parasites does not involve an iron-mediated activation. Thus, the peroxide group appears not to be a crucial pharmacophoric requirement for the tested 1,2-dioxanes, which is divergent from previous findings obtained with the natural endoperoxide artemisinin and ascaridole.^102−104^

Iron is an essential nutrient for all the organisms, including *Leishmania*, and its supply plays a crucial role in the parasite survival.^117,118^ However, the parasitic cells are also vulnerable to the toxicity of iron and iron-induced ROS. Based on the abovementioned results, we investigated the role of iron in the activation of our synthetic compounds **2** and **3**. In contrast with the mentioned previous studies, we showed that the inhibitory activity of **3a** and **2a** against *Leishmania* did not undergo significant variation in the presence of various concentrations of the iron chelators DFO and DFP, suggesting that low-molecular-weight iron species do not play a crucial role in triggering the antileishmanial bioactivity of our synthetic endoperoxides. This result is particularly meaningful for endoperoxide **2a**, and it is supported by the previous observations that tetrahydropyrans **3** and endoperoxides **2** show a similar antileishmanial potency (Table 1) and that they have a comparable activity against both promastigote and amastigote forms of *L. donovani* (Tables 1 and 2).

To further investigate the role of the peroxide bridge on bioactivity, we analyzed the levels of free radicals in *Leishmania* cultures treated with our synthetic compounds **2** and **3**. We observed that the tetrahydropyran **3a** induced a higher increase in intracellular ROS levels than the endoperoxide **2a** in *L. donovani* promastigotes. This finding corroborates our hypothesis that the generation of free radicals by cleaving the O–O bond is not the main mechanism of action of the synthetic peroxides tested in our biological model. Indeed, the peroxide bond in our compounds could be very stable, thus not contributing to the pharmacological activity.

At last, in order to acquire information on the ability of the tested molecules to enter the *L. donovani* promastigote cell and on the compound distribution within the parasite, we carried out confocal microscopy investigations on new synthesized fluorescent endoperoxides and tetrahydropyrans. We detected endoperoxide **2f** and tetrahydropyran **3e** in the parasite cytoplasm and in the kinetoplast but not in the nucleus, while the acridine-labeled endoperoxide **2e** was also found in the nucleus. Although these findings are preliminary, they allow us to make some considerations. It is known that the localization of a labeled species in a cell depends on the structure of both the bioactive compound and the fluorescent tag. By tagging our products with different probes, we observed a common feature, *i*.*e*., a marked accumulation of fluorescent compounds in the parasitic cytoplasm and kinetoplast. Compound **2f**, bearing the nitrobenzyldiazole (NBD) fluorophore, appears as the most similar to the corresponding not labeled bioactive product **2a**, considering its IC_50_ value (Table 4) and calculated log *P* (see the Supporting Information). Therefore, considering the cell localization of the most representative labeled derivative **2f**, we speculate that cytoplasm and kinetoplast are the main accumulation sites of the newly synthesized molecules, where the biological target/s of this family of compounds might be localized.

In conclusion, we synthesized a small library of tetrahydropyrans **3**, bearing the same substitution pattern of the corresponding endoperoxides **2** but lacking the peroxide bridge. Both classes of compounds were tested for their antileishmanial activity, and we observed a similar bioactivity profile for 1,2-dioxanes **2** and tetrahydropyrans **3**. We also found that in our biological systems: (i) iron appeared not to play a crucial role in triggering the activity against *Leishmania* of the selected molecules; (ii) both 1,2-dioxanes **2** and tetrahydropyrans **3** induced a significant oxidative stress in *L. donovani* promastigotes, implying that the cleavage of the O–O bond is not necessary for the generation of free radicals in treated promastigotes; and finally, (iii) fluorescent-tagged 1,2-dioxanes and tetrahydropyrans mainly accumulated in the *Leishmania* cytoplasm and kinetoplast, suggesting that their biological target/s might be localized in these parasitic compartments. Our findings reveal the potential role of both 1,2-dioxanes and tetrahydropyrans as lead compounds for novel therapies against *Leishmania* and provide new insights into their mechanism of action; the peroxide group proved not to be a crucial pharmacophoric requirement for the antileishmanial bioactivity of the new synthesized endoperoxides in our biological system. Additional studies are needed to corroborate our findings.

## Experimental Section

### Chemistry

#### General Information

All the commercial chemicals were purchased from Sigma-Aldrich, VWR, Alfa Aesar, or TCI Chemicals and used without additional purifications. The ^1^H and ^13^C NMR spectra were recorded on a Varian INOVA 400 NMR instrument with a 5 mm probe. All chemical shifts have been quoted relative to deuterated solvent signals; chemical shifts (δ) are reported in ppm, and coupling constants (*J*) are reported in hertz (Hz). Low-resolution MS (LRMS) ESI analyses were performed on an Agilent Technologies MSD1100 single-quadrupole mass spectrometer. Mass spectrometric detection was performed in the full-scan mode from *m/z* 50 to 2500, with a scan time of 0.1 s in the positive ion mode, ESI spray voltage of 4500 V, nitrogen gas pressure of 35 psi, drying gas flow rate of 11.5 mL min^–1^, and fragmentor voltage of 30 V. LRMS EI analyses were performed on a Hewlett-Packard 5971 with EI ionization at 70 eV. High-resolution MS (HRMS) ESI analyses were performed on an LTQ Orbitrap XL (Thermo Scientific) mass spectrometer. Melting point (mp) measurements were performed on Bibby Stuart Scientific SMP3 apparatus. Flash chromatography purifications were carried out using VWR silica gel (40–63 μm particle size). Thin-layer chromatography was performed on Merck 60 F254 plates.

The purity of bioactive target compounds was ≥95% established by HPLC analyses performed on an Agilent Technologies HP1100 instrument. A Phenomenex Gemini C18 3 μm (100 × 3 mm) column was employed for the chromatographic separation, and two different analytical methods were used: method A: mobile phase, H_2_O/CH_3_CN; gradient from 30 to 80% of CH_3_CN in 8 min, 80% of CH_3_CN until 22 min, then up to 90% of CH_3_CN in 2 min, and stop time at 25 min; and flow rate, 0.4 mL min^–1^; method B: gradient analogous to method A employing a mobile phase (H_2_O/CH_3_CN) containing 0.2% of formic acid. The peak identity was confirmed by LRMS ESI analyses.

Endoperoxides **2a**–**2d** are known, and they were prepared according to our previous work.^87^ Compound **4b** is known, and it was synthesized according to the literature procedure.^119^ Its physical and spectroscopic data matched the reported ones. *N*^1^-(6-Chloro-2-methoxyacridin-9-yl)butane-1,4-diamine is a known compound, and it was prepared according to the literature procedure.^120^ Its physical and spectroscopic data matched the reported ones. Benzaldehyde diethyl acetal is known, and it was prepared according to the literature procedure.^121^ After being synthesized, it was used in the following reaction without purification.

#### Synthetic Procedures and Compound Characterizations

##### Synthesis of 2-Phenyl-5,5-disubstituted-1,3-dioxanes (**5**)

Compounds **5** were prepared according to the literature procedure^122^ modified as follows. (1*S*)-(+)-Camphorsulfonic acid (CSA, 0.01 equiv) was added at 0 °C to a solution of the starting diol **4** and benzaldehyde diethyl acetal (1.05 equiv) in anhydrous CH_2_Cl_2_ (2 mL mmol^–1^) under a N_2_ atmosphere. The reaction mixture was warmed to room temperature and stirred for 2–12 h, before being quenched with a saturated aqueous solution of NaHCO_3_. The mixture was extracted with CH_2_Cl_2_ (3 × 10 mL), and the organic phase was dried over Na_2_SO_4_ and filtered. The solvent was removed under reduced pressure, and the product was purified by flash chromatography on silica gel.

##### 5,5-Dibutyl-2-phenyl-1,3-dioxane (**5a**)

Yield: 95%. Colorless liquid. Mobile phase for chromatographic purification: CyH/Et_2_O, 9:1. ^1^H NMR (400 MHz, CDCl_3_): δ 7.49 (dd, *J* = 7.9, 1.8 Hz, 2H), 7.40–7.31 (m, 3H), 5.39 (s, 1H), 3.95 (dd, *J* = 10.3, 1.2 Hz, 2H), 3.60 (dd, *J* = 10.3, 1.2 Hz, 2H), 1.84–1.68 (m, 2H), 1.42–1.32 (m, 2H), 1.32–1.23 (m, 4H), 1.21–1.12 (m, 2H), 1.11–1.03 (m, 2H), 0.94 (t, *J* = 7.2 Hz, 3H), 0.92 (t, *J* = 7.2 Hz, 3H). ^13^C NMR (100 MHz, CDCl_3_): δ 138.5, 128.3, 127.7, 125.8, 101.5, 75.0, 34.3, 32.1, 30.4, 25.1, 24.0, 23.2, 23.2, 13.8, 13.6. LRMS (EI^+^) *m/z* (%): 276 (53) [M]^+^, 275 (92), 245 (5), 140 (49), 105 (65), 56 (100).

##### 2,5,5-Triphenyl-1,3-dioxane (**5b**)

Yield: 94%. White solid. Purified by recrystallization from cold CH_2_Cl_2_. Melting point, 97–101 °C. ^1^H NMR (400 MHz, CDCl_3_): δ 7.58–7.50 (m, 2H), 7.47–7.39 (m, 2H), 7.38–7.29 (m, 7H), 7.25–7.19 (m, 2H), 7.17–7.08 (m, 2H), 5.64 (s, 1H), 4.87 (d, *J* = 11.6 Hz, 2H), 4.42 (d, *J* = 11.6 Hz, 2H). ^13^C NMR (100 MHz, CDCl_3_): δ 144.6, 143.6, 138.2, 129.2, 129.0, 128.8, 128.4, 128.2, 127.0, 126.6, 126.3, 102.3, 75.4, 44.7. LRMS (ESI^+^) *m/z*: 339.2 [M + Na]^+^.

##### 5,5-Dimethyl-2-phenyl-1,3-dioxane (**5c**)

Yield: 87%. The compound is known, and its physical and spectroscopic data matched the reported ones.^122^

##### Synthesis of 3-(Benzyloxy)-2,2-disubstituted-propan-1-ols (**6**)

Compounds **6** were synthesized from the corresponding starting materials **5** according to the literature procedure.^119^

##### 2-((Benzyloxy)methyl)-2-butylhexan-1-ol (**6a**)

Yield: 99%. Colorless liquid. Mobile phase for the chromatographic purification: CyH/AcOEt, 1:1. ^1^H NMR (400 MHz, CDCl_3_): δ 7.40–7.27 (m, 5H), 4.50 (s, 2H), 3.51 (d, *J* = 6.1 Hz, 2H), 3.37 (s, 2H), 2.67 (t, *J* = 5.8 Hz, 1H), 1.35–1.07 (m, 12H), 0.89 (t, *J* = 7.1 Hz, 6H). ^13^C NMR (100 MHz, CDCl_3_): δ 138.0, 127.9, 127.1, 127.0, 75.6, 75.6, 73.0, 67.5, 67.5, 40.6, 30.6, 24.7, 23.3, 13.7. LRMS (EI^+^) *m/z* (%): 278 (2) [M]^+^, 187 (1), 91 (100).

##### 3-(Benzyloxy)-2,2-diphenylpropan-1-ol (**6b**)

Yield: 85%. White waxy solid. Mobile phase for chromatographic purification: CyH/Et_2_O, 8:2. ^1^H NMR (400 MHz, CDCl_3_): δ 7.39–7.26 (m, 7H), 7.25–7.16 (m, 8H), 4.58 (s, 2H), 4.36 (d, *J* = 6.4 Hz, 2H), 4.14 (s, 2H), 2.50 (t, *J* = 6.5 Hz, 1H). ^13^C NMR (100 MHz, CDCl_3_): δ 143.8, 137.6, 128.6, 128.2, 128.1, 127.8, 127.7, 126.5, 76.2, 73.6, 68.8, 52.6. LRMS (ESI^+^) *m/z*: 301.2 [M – OH]^+^, 319.2 [M + H]^+^, 341.2 [M + Na]^+^.

##### 3-(Benzyloxy)-2,2-dimethylpropan-1-ol (**6c**)

The compound is known, and its physical and spectroscopic data matched the reported ones.^122^

##### Synthesis of 3-(Benzyloxy)-2,2-disubstituted-propanals (**7**)

Compounds **7** were synthesized from the corresponding starting materials **6** according to the literature procedure.^122^

##### 2-((Benzyloxy)methyl)-2-butylhexanal (**7a**)

Yield: 86%. Colorless liquid. Mobile phase for the chromatographic purification: CyH/Et_2_O, 9:1. ^1^H NMR (400 MHz, CDCl_3_): δ 9.47 (s, 1H), 7.31–7.18 (m, 5H), 4.43 (s, 2H), 3.46 (s, 2H), 1.52 (ddd, *J* = 13.8, 9.0, 5.7 Hz, 4H), 1.27 (h, *J* = 7.3 Hz, 4H), 1.11 (dq, *J* = 7.9, 4.4 Hz, 4H), 0.87 (t, *J* = 7.3 Hz, 6H). ^13^C NMR (100 MHz, CDCl_3_): δ 205.2, 137.9, 128.0, 127.2, 127.2, 73.0, 70.3, 52.9, 29.5, 25.2, 23.1, 13.6. LRMS (EI^+^) *m/z* (%): 276 (<1) [M]^+^, 113 (15), 91 (100).

##### 3-(Benzyloxy)-2,2-diphenylpropanal (**7b**)

Yield: 95%. White waxy solid. Mobile phase for the chromatographic purification: CyH/AcOEt, 95:5. ^1^H NMR (400 MHz, CDCl_3_): δ 9.91 (s, 1H), 7.41–7.27 (m, 8H), 7.25–7.11 (m, 7H), 4.52 (s, 2H), 4.25 (s, 2H). ^13^C NMR (100 MHz, CDCl_3_): δ 199.0, 138.8, 137.8, 129.2, 128.5, 128.3, 127.5, 127.4, 73.6, 72.5, 64.1. LRMS (ESI^+^) *m/z*: 299.2 [M – OH]^+^, 317.2 [M + H]^+^, 334.2 [M + NH_4_]^+^, 339.2 [M + Na]^+^.

##### 3-(Benzyloxy)-2,2-dimethylpropanal (**7c**)

The compound is known, and its physical and spectroscopic data matched the reported ones.^122^

##### Synthesis of the Knöevenagel Adducts (**8**)

Compounds **8** were synthesized according to the literature procedure^106^ from the corresponding starting materials **7** using the commercially available preformed titanium complex (2 equiv) and by stirring the reaction mixture at reflux for 18 h.

##### Methyl (*E*)-2-Acetyl-4-((benzyloxy)methyl)-4-butyloct-2-enoate (*E*-**8a**)

Total (*Z* and *E* isomers) yield: 47%. Yellowish liquid. Mobile phase for the chromatographic purification: CyH/AcOEt, 95:5. ^1^H NMR (400 MHz, CDCl_3_): δ 7.37–7.25 (m, 5H), 6.69 (s, 1H), 4.40 (s, 2H), 3.77 (s, 3H), 3.31 (s, 2H), 2.30 (s, 3H), 1.49–1.41 (m, 4H), 1.26 (q, *J* = 7.3 Hz, 6H), 1.19–1.07 (m, 2H), 0.87 (t, *J* = 7.2 Hz, 6H). ^13^C NMR (100 MHz, CDCl_3_): δ 202.5, 165.4, 151.3, 138.2, 128.2, 127.6, 127.5, 72.6, 72.4, 52.2, 44.9, 35.8, 31.3, 25.9, 23.3, 13.9. LRMS (ESI^+^) *m/z*: 343.2 [M – OMe]^+^, 392.4 [M + NH_4_]^+^.

##### Methyl (*Z*)-2-Acetyl-4-((benzyloxy)methyl)-4-butyloct-2-enoate (*Z*-**8a**)

Total (*Z* and *E* isomers) yield: 47%. Yellowish liquid. Mobile phase for the chromatographic purification: CyH/AcOEt, 95:5. ^1^H NMR (400 MHz, CDCl_3_): δ 7.39–7.27 (m, 5H), 6.65 (s, 1H), 4.48 (s, 2H), 3.74 (s, 3H), 3.38 (s, 2H), 2.28 (s, 3H), 1.52–1.42 (m, 3H), 1.26 (h, *J* = 7.3 Hz, 5H), 1.20–1.07 (m, 4H), 0.87 (t, *J* = 7.2 Hz, 6H). ^13^C NMR (100 MHz, CDCl_3_): δ 195.3, 168.1, 151.8, 138.2, 135.9, 128.2, 127.5, 73.0, 72.6, 51.9, 44.7, 34.9, 25.9, 25.8, 23.2, 13.9. LRMS (ESI^+^) *m/z*: 343.2 [M – OMe]^+^, 392.4 [M + NH_4_]^+^.

##### Methyl 2-Acetyl-5-(benzyloxy)-4,4-diphenylpent-2-enoate (**8b**)

Yield: 31%. Yellowish oil. Mobile phase for the chromatographic purification: CyH/AcOEt, 9:1. ^1^H NMR (400 MHz, CDCl_3_): δ 7.52 (s, 1H), 7.33–7.26 (m, 8H), 7.25–7.18 (m, 7H), 4.52 (s, 2H), 4.12 (s, 2H), 3.11(s, 3H), 2.35 (s, 3H). ^13^C NMR (100 MHz, CDCl_3_): δ 195.4, 166.3, 149.0, 141.0, 137.5, 136.9, 128.9, 128.2, 128.0, 127.6, 127.6, 127.0, 75.5, 73.3, 55.3, 51.4, 26.5. LRMS (ESI^+^) *m/z*: 415.2 [M + H]^+^, 432.2 [M + NH_4_]^+^, 437.2 [M + Na]^+^.

##### Methyl (*E*)-2-Acetyl-5-(benzyloxy)-4,4-dimethylpent-2-enoate (*E*-**8c**)

Total (*Z* and *E* isomers) yield: 92%. Yellowish oil. Mobile phase for the chromatographic purification: CyH/AcOEt, 95:5. ^1^H NMR (400 MHz, CDCl_3_): δ 7.36–7.25 (m, 5H), 6.82 (s, 1H), 4.47 (s, 2H), 3.75 (s, 3H), 3.24 (s, 2H), 2.35 (d, *J* = 1.2 Hz, 3H), 1.10 (s, 6H). ^13^C NMR (100 MHz, CDCl_3_): δ 203.1, 165.2, 151.3, 138.1, 133.8, 128.2, 127.5, 127.4, 78.0, 72.9, 52.2, 38.6, 31.7, 24.6. LRMS (ESI^+^) *m/z*: 291.2 [M + H]^+^, 308.2 [M + NH_4_]^+^, 313.2 [M + Na]^+^.

##### Methyl (*Z*)-2-Acetyl-5-(benzyloxy)-4,4-dimethylpent-2-enoate (*Z*-**8c**)

Total (*Z* and *E* isomers) yield: 92%. Yellowish oil. Mobile phase for the chromatographic purification: CyH/AcOEt, 95:5. ^1^H NMR (400 MHz, CDCl_3_): δ 7.36–7.26 (m, 5H), 6.82 (s, 1H), 4.51 (s, 2H), 3.78 (s, 3H), 3.27 (s, 2H), 2.27 (s, 3H), 1.13 (s, 6H). ^13^C NMR (100 MHz, CDCl_3_): δ 195.6, 168.1, 151.7, 138.2, 135.6, 128.3, 127.6, 127.5, 78.6, 73.2, 52.1, 38.5, 25.9, 23.6. LRMS (ESI^+^) *m/z*: 291.2 [M + H]^+^, 308.2 [M + NH_4_]^+^, 313.2 [M + Na]^+^.

##### Synthesis of Methyl Tetrahydropyran-3-carboxylates (**9**)

The appropriate starting materials **8** were added to a suspension of 10% Pd/C (20% w/w with respect to the substrate) in MeOH (10 mL mmol^–1^), and the mixture was vigorously stirred at room temperature under a H_2_ atmosphere (1 atm) for 12–16 h. The reaction mixture was then filtered, and the solvent was removed under reduced pressure. The product was isolated from the crude by flash chromatography on silica gel.

##### Methyl 5,5-Dibutyl-2-methoxy-2-methyltetrahydro-2*H*-pyran-3-carboxylate (**9a**)

Yield: 56%. Colorless oil. Mobile phase for the chromatographic purification: CyH/AcOEt, 95:5. ^1^H NMR (400 MHz, CDCl_3_): δ 3.68 (d, *J* = 1.0 Hz, 3H), 3.32 (d, *J* = 11.1 Hz, 1H), 3.24 (dd, *J* = 11.1, 2.5 Hz, 1H), 3.19 (s, 3H), 2.70 (dd, *J* = 13.3, 4.3 Hz, 1H), 1.88 (t, *J* = 13.4 Hz, 1H), 1.54 (ddd, *J* = 13.6, 4.3, 2.4 Hz, 1H), 1.46 (s, 3H), 1.40 (ddd, *J* = 9.9, 5.6, 3.5 Hz, 2H), 1.35–1.04 (m, 10H), 0.90 (dt, *J* = 8.4, 7.1 Hz, 6H). ^13^C NMR (100 MHz, CDCl_3_): δ 172.6, 97.3, 67.8, 51.6, 48.0, 47.0, 36.3, 34.1, 31.5, 30.9, 25.4, 24.7, 23.5, 23.5, 22.8, 14.1, 14.0. LRMS (ESI^+^) *m/z*: 269.2 [M – OMe]^+^, 301.2 [M + H]^+^, 323.2 [M + Na]^+^, 623.4 [2 M + Na]^+^.

##### Methyl 2-Methoxy-2-methyl-5,5-diphenyltetrahydro-2*H*-pyran-3-carboxylate (**9b**)

Yield: 72%. White solid. Mobile phase for the chromatographic purification: CyH/AcOEt, 95:5. ^1^H NMR (400 MHz, CDCl_3_): δ 7.41–7.39 (m, 2H), 7.31–7.25 (m, 3H), 7.22–7.18 (m, 5H), 4.28 (dd, *J* = 11.9, 2.9 Hz, 1H), 3.71 (d, *J* = 11.8 Hz, 1H), 3.68 (s, 3H), 3.23 (s, 3H), 3.09 (t, *J* = 12.9 Hz, 1H), 2.44 (dd, *J* = 12.8, 3.4 Hz, 1H), 2.37 (dt, *J* = 12.9, 3.2 Hz, 1H), 1.43 (s, 3H). ^13^C NMR (100 MHz, CDCl_3_): δ 172.2, 145.7, 144.7, 128.3, 127.0, 126.4, 126.0, 97.3, 67.2, 51.7, 48.1, 47.5, 45.7, 32.4, 22.6. LRMS (ESI^+^) *m/z*: 309.2 [M – OMe]^+^, 363.2 [M + Na]^+^.

##### Methyl 2-Methoxy-2,5,5-trimethyltetrahydro-2*H*-pyran-3-carboxylate (**9c**)

Yield: 63%. Colorless oil. Mobile phase for the chromatographic purification: CyH/AcOEt, 95:5. ^1^H NMR (400 MHz, CDCl_3_): δ 3.69 (s, 3H), 3.38 (d, *J* = 10.9 Hz, 1H), 3.20 (s, 3H), 3.09 (dd, *J* = 10.9, 2.6 Hz, 1H), 2.74 (dd, *J* = 13.3, 4.3 Hz, 1H), 2.00 (t, *J* = 13.3 Hz, 1H), 1.48 (s, 3H), 1.41 (ddd, *J* = 13.4, 4.4, 2.8 Hz, 1H), 1.02 (s, 3H), 0.87 (s, 3H). ^13^C NMR (100 MHz, CDCl_3_): δ 172.0, 96.8, 69.8, 51.3, 47.7, 47.3, 34.7, 29.3, 26.6, 23.0, 22.5. LRMS (ESI^+^) *m/z*: 185.2 [M – OMe]^+^, 239.2 [M + Na]^+^, 455.2 [2 M + Na]^+^.

##### Synthesis of 3-Hydroxymethyltetrahydropyrans (**10**)

Compounds **10** were synthesized from the corresponding starting materials **9** according to our previously reported procedure.^87^

##### (5,5-Dibutyl-2-methoxy-2-methyltetrahydro-2*H*-pyran-3-yl)methanol (**10a**)

Yield: 81%. Colorless oil. Mobile phase for the chromatographic purification: CyH/AcOEt, 9:1. ^1^H NMR (400 MHz, CDCl_3_): δ 3.90 (d, *J* = 11.4 Hz, 1H), 3.43 (t, *J* = 10.4 Hz, 1H), 3.26 (d, *J* = 1.2 Hz, 2H), 3.22 (s, 3H), 2.86 (d, *J* = 9.8 Hz, 1H), 1.81–1.70 (m, 2H), 1.45 (dd, *J* = 12.2, 5.0 Hz, 2H), 1.40 (s, 3H), 1.38–1.04 (m, 10H), 0.90 (q, *J* = 7.3 Hz, 6H). ^13^C NMR (100 MHz, CDCl_3_): δ 100.6, 68.1, 63.9, 47.5, 41.3, 36.6, 34.9, 31.8, 31.1, 25.5, 24.6, 23.6, 23.5, 21.9, 14.1, 14.0. LRMS (ESI^+^) *m/z*: 273.2 [M + H]^+^.

##### (2-Methoxy-2-methyl-5,5-diphenyltetrahydro-2*H*-pyran-3-yl)methanol (**10b**)

Yield: 95%. White waxy solid. Mobile phase for the chromatographic purification: CyH/AcOEt, 75:25. ^1^H NMR (400 MHz, CDCl_3_): δ 7.48–7.35 (m, 2H), 7.34–7.12 (m, 8H), 4.32 (dd, *J* = 11.9, 3.0 Hz, 1H), 3.90 (dd, *J* = 11.6, 2.8 Hz, 1H), 3.66 (d, *J* = 11.9 Hz, 1H), 3.43 (dd, *J* = 11.7, 3.4 Hz, 1H), 3.26 (s, 3H), 3.00 (t, *J* = 13.0 Hz, 1H), 2.17 (dt, *J* = 13.0, 3.2 Hz, 1H), 1.50 (dt, *J* = 13.0, 3.2 Hz, 1H), 1.37 (s, 3H). ^13^C NMR (100 MHz, CDCl_3_): δ 146.2, 145.5, 128.3, 128.3, 128.1, 126.9, 126.4, 125.8, 100.4, 67.5, 63.9, 47.6, 46.6, 41.8, 32.9, 21.7. LRMS (ESI^+^) *m/z*: 313.2 [M + H]^+^.

##### (2-Methoxy-2,5,5-trimethyltetrahydro-2*H*-pyran-3-yl)methanol (**10c**)

Yield: 47%. Colorless oil. Mobile phase for the chromatographic purification: CyH/AcOEt, 75:25. ^1^H NMR (400 MHz, CDCl_3_): δ 3.91 (dd, *J* = 11.5, 2.7 Hz, 1H), 3.44 (bt, *J* = 10.4 Hz, 1H), 3.32 (d, *J* = 10.9 Hz, 1H), 3.23 (s, 3H), 3.10 (dd, *J* = 10.9, 2.6 Hz, 1H), 2.87 (bd, *J* = 9.6 Hz, 1H), 1.87 (t, *J* = 12.9 Hz, 1H), 1.83–1.75 (m, 1H), 1.41 (s, 3H), 1.21 (dt, *J* = 12.5, 3.1 Hz, 1H), 1.04 (s, 3H), 0.86 (s, 3H). ^13^C NMR (100 MHz, CDCl_3_): δ 100.0, 70.3, 63.6, 47.4, 42.0, 35.2, 30.2, 27.1, 23.5, 21.8. LRMS (ESI^+^) *m/z*: 189.1 [M + H]^+^.

##### Synthesis of Tetrahydropyran-3-carboxyaldehydes (**11**)

Compounds **11** were synthesized from the corresponding starting materials **10** according to our previously reported procedure,^87^ and they were immediately used in the following step.

##### Synthesis of 3-Aminopropyltetrahydropyrans (**3**)

Compounds **3** were synthesized from the corresponding starting materials **11** according to our previously reported procedure.^87^

##### *N*-((5,5-Dibutyl-2-methoxy-2-methyltetrahydro-2*H*-pyran-3-yl)methyl)-3-(1*H*-imidazol-1-yl)propan-1-amine (**3a**)

Yield: 45%. Colorless oil. Mobile phase for the chromatographic purification: AcOEt/MeOH, 6:4. ^1^H NMR (400 MHz, CDCl_3_): δ 7.46 (s, 1H), 7.04 (s, 1H), 6.90 (s, 1H), 4.02 (t, *J* = 6.9 Hz, 2H), 3.28–3.18 (m, 2H), 3.15 (s, 3H), 2.64 (dd, *J* = 11.8, 3.8 Hz, 1H), 2.54 (t, *J* = 6.8 Hz, 2H), 2.42 (dd, *J* = 11.8, 7.8 Hz, 1H), 1.91 (p, *J* = 6.8 Hz, 2H), 1.74 (ddt, *J* = 12.1, 7.8, 4.0 Hz, 1H), 1.52–1.39 (m, 4H), 1.32 (s, 3H), 1.38–1.02 (m, 10H), 0.89 (q, *J* = 7.3 Hz, 6H). ^13^C NMR (100 MHz, CDCl_3_): δ 137.2, 129.4, 118.8, 99.1, 67.8, 51.4, 47.6, 46.6, 44.7, 40.8, 36.6, 34.8, 33.8, 31.2, 31.2, 25.5, 24.7, 23.6, 23.5, 22.0, 14.2, 14.0. LRMS (ESI^+^) *m/z*: 348.4 [M – OMe]^+^, 402.4 [M + Na]^+^. HRMS (ESI^+^) *m/z*: [M + Na]^+^ calcd for C_22_H_41_N_3_NaO_2_, 402.3096; found, 402.3104.

##### 3-(1*H*-Imidazol-1-yl)-*N*-((2-methoxy-2-methyl-5,5-diphenyltetrahydro-2*H*-pyran-3-yl)methyl)propan-1-amine (**3b**)

Yield: 85%. White waxy solid. Mobile phase for the chromatographic purification: AcOEt/MeOH, 8:2. ^1^H NMR (400 MHz, CD_3_OD): δ 7.61 (d, *J* = 1.3 Hz, 1H), 7.47–7.39 (m, 2H), 7.34–7.11 (m, 8H), 7.10 (s, 1H), 6.96 (s, 1H), 4.33 (dd, *J* = 11.9, 2.8 Hz, 1H), 4.03 (td, *J* = 7.0, 1.6 Hz, 2H), 3.63 (d, *J* = 11.8 Hz, 1H), 3.21 (s, 3H), 2.66 (dd, *J* = 12.4, 3.5 Hz, 1H), 2.61–2.52 (m, 2H), 2.49 (d, *J* = 12.3 Hz, 1H), 2.47–2.38 (m, 2H), 1.99–1.87 (m, 2H), 1.56 (ddd, *J* = 12.1, 8.2, 4.0 Hz, 1H), 1.27 (s, 3H). ^13^C NMR (100 MHz, CD_3_OD): δ 147.9, 147.0, 138.4, 129.5, 129.3, 129.1, 129.1, 127.9, 127.3, 126.9, 120.5, 100.2, 68.3, 51.7, 48.1, 47.5, 47.4, 45.9, 42.1, 35.3, 31.5, 22.1. LRMS (ESI^+^) *m/z*: 388.2 [M – OMe]^+^, 420.2 [M + H]^+^. HRMS (ESI^+^) *m/z*: [M + H]^+^ calcd for C_26_H_34_N_3_O_2_, 420.2651; found, 420.2657.

##### 3-(1*H*-Imidazol-1-yl)-*N*-((2-methoxy-2,5,5-trimethyltetrahydro-2*H*-pyran-3-yl)methyl)propan-1-amine (**3c**)

Yield: 99%. Colorless oil. Mobile phase for the chromatographic purification: CH_2_Cl_2_/MeOH/NH_3_, 9:1:0.1. ^1^H NMR (400 MHz, CDCl_3_): δ 7.47 (s, 1H), 7.05 (d, *J* = 1.2 Hz, 1H), 6.91 (d, *J* = 1.3 Hz, 1H), 4.03 (t, *J* = 7.0 Hz, 2H), 3.30 (d, *J* = 10.8 Hz, 1H), 3.17 (s, 3H), 3.06 (dd, *J* = 10.8, 2.4 Hz, 1H), 2.66 (dd, *J* = 11.8, 3.8 Hz, 1H), 2.55 (t, *J* = 6.8 Hz, 2H), 2.46 (dd, *J* = 11.9, 7.8 Hz, 1H), 1.92 (p, *J* = 6.9 Hz, 2H), 1.79 (ddt, *J* = 12.3, 8.1, 4.3 Hz, 1H), 1.51–1.40 (m, 1H), 1.41–1.34 (m, 1H), 1.34 (s, 3H), 1.03 (s, 3H), 0.83 (s, 3H). ^13^C NMR (100 MHz, CDCl_3_): δ 137.0, 129.1, 118.7, 98.8, 70.1, 51.1, 47.5, 46.5, 44.6, 41.4, 37.2, 31.1, 30.1, 27.1, 23.6, 21.8. LRMS (ESI^+^) *m/z*: 296.2 [M + H]^+^, 559.8 [2M – OMe]^+^. HRMS (ESI^+^) *m/z*: [M + H]^+^ calcd for C_16_H_30_N_3_O_2_, 296.2338; found, 296.2332.

##### Synthesis of Tetrahydropyran-3-propargyl Ether (**12**)

Compound **12** was synthesized from the corresponding starting material **10b** according to our previously reported procedure.^87^

##### 2-Methoxy-2-methyl-5,5-diphenyl-3-((prop-2-yn-1-yloxy)methyl)tetrahydro-2*H*-pyran (**12**)

Yield: 77%. Colorless waxy solid. Mobile phase for the chromatographic purification: CyH/AcOEt, 95:5 to 8:2. ^1^H NMR (400 MHz, CDCl_3_): δ 7.46–7.40 (m, 2H), 7.32–7.22 (m, 4H), 7.21–7.14 (m, 4H), 4.30 (dd, *J* = 11.8, 2.9 Hz, 1H), 4.06 (d, *J* = 2.4 Hz, 2H), 3.71–3.65 (m, 2H), 3.35 (dd, *J* = 9.4, 7.9 Hz, 1H), 3.20 (s, 3H), 2.48 (t, *J* = 12.5 Hz, 2H), 2.38 (t, *J* = 2.4 Hz, 1H), 1.76 (ddt, *J* = 12.2, 8.5, 4.2 Hz, 1H), 1.31 (s, 3H). ^13^C NMR (100 MHz, CDCl_3_): δ 146.5, 145.4, 128.3, 128.2, 128.1, 126.9, 126.3, 125.8, 98.3, 79.9, 74.2, 71.5, 67.2, 58.1, 47.7, 46.1, 41.2, 33.7, 22.0. LRMS (ESI^+^) *m/z*: 319.2 [M – OMe]^+^, 373.2 [M + Na]^+^.

##### Synthesis of Tetrahydropyran-3-triazolyl Ether (**3d**)

Compound **3d** was synthesized from the corresponding starting material **12** according to our previously reported procedure.^87^

##### (3-(4-(((2-Methoxy-2-methyl-5,5-diphenyltetrahydro-2*H*-pyran-3-yl)methoxy)methyl)-1*H*-1,2,3-triazol-1-yl)propyl)triphenylphosphonium bromide (**3d**)

Yield: 51%. Pinkish waxy solid. Mobile phase for the chromatographic purification: CH_2_Cl_2_/MeOH, 9:1. ^1^H NMR (400 MHz, CD_3_OD): δ 8.13 (bs, 1H), 7.95–7.79 (m, 4H), 7.81–7.69 (m, 11H), 7.38 (d, *J* = 7.7 Hz, 2H), 7.22 (td, *J* = 7.5, 4.7 Hz, 4H), 7.13 (tt, *J* = 9.0, 5.3 Hz, 4H), 4.63 (t, *J* = 5.7 Hz, 2H), 4.49 (s, 2H), 4.31 (d, *J* = 11.8 Hz, 1H), 3.71 (bs, 1H), 3.60 (d, *J* = 11.8 Hz, 1H), 3.53–3.34 (m, 3H), 3.17 (s, 3H), 2.45–2.36 (m, 2H), 2.35–2.22 (m, 2H), 1.78–1.59 (bs, 1H), 1.23 (s, 3H). ^13^C NMR (100 MHz, CD_3_OD): δ 146.5, 145.4, 135.2 (d, *J* = 3.0 Hz), 133.8 (d, *J* = 10.0 Hz), 130.6 (d, *J* = 12.6 Hz), 128.3, 128.2, 128.1, 126.9, 126.1, 125.7, 117.8 (d, *J* = 86.4 Hz), 98.3, 72.2, 67.1, 60.3, 46.9 (d, *J* = 166.7 Hz), 41.2, 33.7, 29.6, 23.4 (d, *J* = 2.3 Hz), 22.1, 21.0, 20.0 (d, *J* = 52.9 Hz), 14.1. LRMS (ESI^+^) *m/z*: 332.8 [M – OMe]^2+^, 696.6 [M]^+^. HRMS (ESI^+^) *m/z*: [M – Br^–^]^+^ calcd for C_44_H_47_N_3_O_3_P^+^, 696.3350; found, 696.3360.

##### Synthesis of Acridine-Labeled Compounds

Compounds **2e** and **3e** were synthesized from the corresponding starting materials (**13**^87^ and **11a**, respectively) and *N*^1^-(6-chloro-2-methoxyacridin-9-yl)butane-1,4-diamine following our previously reported procedure.^87^

##### *N*^1^-(6-Chloro-2-methoxyacridin-9-yl)-*N*^4^-((6,6-dibutyl-3-methoxy-3-methyl-1,2-dioxan-4-yl)methyl)butane-1,4-diamine (**2e**)

Yield: 45%. Yellow waxy solid. Mobile phase for the chromatographic purification: CH_2_Cl_2_/MeOH/NH_3_, 9:1:0.1. ^1^H NMR (400 MHz, CDCl_3_): δ 8.04 (d, *J* = 2.1 Hz, 1H), 8.01 (d, *J* = 9.3 Hz, 1H), 7.97 (d, *J* = 9.4 Hz, 1H), 7.39 (dd, *J* = 9.5, 2.6 Hz, 1H), 7.28 (dd, *J* = 9.2, 2.1 Hz, 1H), 7.23 (d, *J* = 2.7 Hz, 1H), 3.95 (s, 3H), 3.73 (t, *J* = 6.9 Hz, 2H), 3.26 (s, 3H), 2.74 (dd, *J* = 11.9, 4.0 Hz, 1H), 2.64 (td, *J* = 6.7, 1.5 Hz, 2H), 2.50 (dd, *J* = 11.9, 8.1 Hz, 1H), 1.94 (ddt, *J* = 12.4, 8.4, 4.4 Hz, 1H), 1.87–1.74 (m, 3H), 1.69–1.55 (m, 4H), 1.50 (dd, *J* = 13.2, 5.0 Hz, 1H), 1.47–1.26 (m, 4H), 1.25 (s, 3H), 1.24–1.08 (m, 6H), 0.86 (dt, *J* = 16.4, 7.1 Hz, 6H). ^13^C NMR (100 MHz, CDCl_3_): δ 155.8, 149.9, 148.0, 134.9, 131.0, 124.3, 124.2, 117.7, 115.5, 102.2, 99.6, 81.8, 55.5, 50.8, 50.5, 49.7, 48.6, 39.2, 36.4, 32.2, 31.5, 29.5, 27.5, 25.5, 24.8, 23.2, 23.2, 18.9, 14.1, 13.9. LRMS (ESI^+^) *m/z*: 277.8 [M – OMe + H]^2+^, 586.6 [M + H]^+^.

##### *N*^1^-(6-Chloro-2-methoxyacridin-9-yl)-*N*^4^-((5,5-dibutyl-2-methoxy-2-methyltetrahydro-2*H*-pyran-3-yl)methyl)butane-1,4-diamine (**3e**)

Yield: 32%. Yellow waxy solid. Mobile phase for the chromatographic purification: CH_2_Cl_2_/MeOH/NH_3_, 9:1:0.1. ^1^H NMR (400 MHz, CDCl_3_): δ 8.05 (d, *J* = 9.3 Hz, 1H), 8.02 (d, *J* = 2.0 Hz, 1H), 7.93 (d, *J* = 9.3 Hz, 1H), 7.36 (d, *J* = 2.5 Hz, 1H), 7.31 (d, *J* = 9.4 Hz, 1H), 7.22 (dd, *J* = 9.3, 2.1 Hz, 1H), 3.97 (s, 3H), 3.84 (t, *J* = 6.8 Hz, 2H), 3.16 (s, 3H), 2.87–2.67 (m, 3H), 2.58 (dd, *J* = 12.1, 7.9 Hz, 1H), 2.00–1.85 (m, 2H), 1.85–1.72 (m, 2H), 1.57 (d, *J* = 13.0 Hz, 1H), 1.48–1.36 (m, 2H), 1.34 (s, 3H), 1.32–0.98 (m, 14H), 0.86 (dt, *J* = 10.3, 7.1 Hz, 6H). LRMS (ESI^+^) *m/z*: 276.2 [M + H – OMe]^2+^, 584.6 [M + H]^+^.

##### Synthesis of NBD-Labeled Endoperoxide (**2f**)

Compound **14** was prepared from **13**(87) and the commercially available *N*-Boc-1,4-butanediamine following our previously reported procedure.^87^

##### *tert*-Butyl (4-(((6,6-Dibutyl-3-methoxy-3-methyl-1,2-dioxan-4-yl)methyl)amino)butyl)carbamate (**14**)

Yield: 79%. Colorless waxy solid. Mobile phase for the chromatographic purification: CH_2_Cl_2_/MeOH, 9:1. ^1^H NMR (400 MHz, CDCl_3_): δ 4.76 (bs, 1H), 3.30 (s, 3H), 3.14 (d, *J* = 6.6 Hz, 2H), 2.87 (bs, 1H), 2.68 (bs, 2H), 2.07 (bs, 1H), 1.95–1.83 (m, 1H), 1.74–1.46 (m, 14H), 1.44 (s, 9H), 1.32 (s, 3H), 1.37–1.23 (m, 4H), 0.91 (dt, *J* = 9.1, 6.8 Hz, 6H). ^13^C NMR (100 MHz, CDCl_3_): δ 156.0, 102.0, 81.7, 50.5, 49.5, 48.5, 40.2, 38.8, 36.3, 32.1, 31.4, 28.3, 27.7, 26.6, 25.4, 24.7, 23.1, 18.7, 14.0, 13.8. LRMS (ESI^+^) *m/z*: 445.6 [M + H]^+^, 711.8 [2M – Boc]^+^.

Compound **14** was subsequently converted into the corresponding *N*-deprotected hydrochloride salt according to a literature procedure,^100^ and it was immediately used in the following synthetic step without any purification.

Compound **2f** was prepared according to a literature procedure^100^ modified as follows: 4-chloro-7-nitrobenzofurazan (1.1 equiv) was added to a solution of the hydrochloride salt and triethylamine (3 equiv) in anhydrous CH_2_Cl_2_ (30 mL mmol^–1^) at 0 °C under a N_2_ atmosphere. The reaction mixture was warmed at room temperature and stirred for 4 h. The solvent was removed under reduced pressure, and the product was isolated by flash chromatography on silica gel.

##### *N*^1^-((6,6-Dibutyl-3-methoxy-3-methyl-1,2-dioxan-4-yl)methyl)-*N*^4^-(7-nitrobenzo[*c*][1,2,5]oxadiazol-4-yl)butane-1,4-diamine (**2f**)

Yield: 21%. Dark waxy solid. Mobile phase for the chromatographic purification: CH_2_Cl_2_/MeOH, 9:1 to 8:2. ^1^H NMR (400 MHz, CD_3_OD): δ 8.50 (d, *J* = 8.9 Hz, 1H), 6.36 (d, *J* = 8.9 Hz, 1H), 3.75–3.49 (m, 2H), 3.27 (s, 3H), 3.07–2.93 (m, 1H), 2.89 (t, *J* = 7.4 Hz, 2H), 2.79 (dd, *J* = 12.5, 8.5 Hz, 1H), 2.28–2.07 (m, 1H), 1.96–1.71 (m, 5H), 1.57–1.46 (m, 1H), 1.37–1.30 (m, 2H), 1.31 (s, 3H), 1.31–1.19 (m, 10H), 0.97–0.82 (m, 6H). LRMS (ESI^+^) *m/z*: 508.6 [M + H]^+^, 1015.0 [2 M + H]^+^.

### Parasitology

#### Parasites

Promastigotes of a *L. donovani* reference strain (MHOM/NP/02/BPK282/0cl4) were maintained at 26 °C in a liquid custom-made medium, HOMEM Cat. no: 1140-082 (Gibco Thermo Fisher Scientific Inc., Waltham, USA), complete composition: S-MEM (Eagle) 1 × 1 liter pack, glucose (1–2 g), sodium bicarbonate (0.3 g), sodium pyruvate (0.11 g), *p*-aminobenzoic acid (1 mg), biotin (0.1 mg), HEPES (4.77–5.96 g), MEM amino acids (10 mL), MEM nonessential amino acids (10 mL), pH 7.5–7.6, supplemented with 20% fetal bovine serum (FBS, EuroClone SpA, Milan, Italy)^123^ and 1% penicillin–streptomycin (EuroClone SpA).

#### Cell Cultures

Vero cells (kidney of African green monkey epithelial cell line) were cultured at 37 °C in the MEM liquid medium supplemented with 10% FBS (EuroClone SpA), 1% levoglutamine (EuroClone SpA), and 1% penicillin–streptomycin (EuroClone SpA). THP-1 cells (human leukemia monocytic cell line) were cultured at 37 °C in the RPMI 1640 (EuroClone SpA) liquid medium supplemented with 10% FBS (EuroClone SpA), 1% levoglutamine (EuroClone SpA), 1% penicillin–streptomycin, and mercaptoethanol (Gibco) 50 μM.

#### Promastigote Growth Inhibition Assay

To evaluate the efficacy of the compounds on the extracellular forms of *L. donovani*, promastigotes in their late log/stationary phase were seeded with the complete HOMEM medium at 10^6^ cells mL^–1^ in 96-well plates and incubated with tested compounds at a range concentration of 600–1.6 μM in a 26 °C incubator for 72 h. The antileishmanial drug amphotericin B was used as the positive control, and untreated promastigotes were the negative control. Each experiment was performed in duplicate. Stock solution of the compounds was 8 mM in DMSO. To estimate the concentration at which the compounds caused 50% inhibition of growth (IC_50_), the alamarBlue assay was employed (Life Technologies, Thermo Fisher Scientific Inc., Waltham, USA). The alamarBlue assay includes a colorimetric growth indicator based on detection of metabolic activity. Specifically, the system incorporates an oxidation–reduction (REDOX) indicator that changes color in response to chemical reduction of the growth medium resulting from cell growth. The method monitors the reducing environment of proliferating cells; the cell-permeable resazurin is added (nonfluorescent form, blue color) and, upon entering cells, is reduced to resorufin (fluorescent form, red color) as a result of cellular metabolic activity. Evaluation was performed by adding 20 μL of alamarBlue and incubating at 26 °C for 24 h. The reducing environment was evaluated after 24 h by absorbance measurement at the Multiskan ascent plate reader (Thermo Fisher Scientific Inc.) at 550 and 630 nm.

#### Cytotoxicity Test

The cytotoxicity of the compounds was determined using mammalian kidney epithelial cells (Vero cell line) and human acute monocytic leukemia cell line (THP-1). Cells were seeded (10^5^ cells mL^–1^) on 96-well plates with the complete MEM medium and incubated with test compounds at 37 °C in a 5% CO_2_ incubator. After 72 h of incubation, 20 μL of the alamarBlue reagent was added to each well and incubated at 37 °C for 24 h. Reduction of resazurin to resorufin was evaluated after 24 h by absorbance measurement at the Multiskan ascent plate reader (Thermo Fisher Scientific Inc.,) at 550 and 630 nm. DMSO used for compound dilution was also tested to control the employed concentration, which was not toxic and did not influence the toxicity of the compounds. Each experiment was performed in duplicate. The selectivity index (SI) for each compound was calculated as the ratio between cytotoxicity (CC_50_/72 h) and activity (IC_50_/72 h) against *Leishmania* promastigotes.

#### Amastigote Growth Inhibition Assay

To evaluate the efficacy of the compounds on the intracellular form of *L. donovani*, human acute monocytic leukemia cell line (THP-1) was infected with *L. donovani* promastigotes. Cells were harvested and seeded in a 96-well plate (10^5^ cells mL^–1^) in the complete RPMI 1640 medium, and PMA (0.1 μM, Cayman Chemical Company, Ann Arbor, Michigan, USA) was added for obtaining maturation and cell adherence. Cells were incubated at 37 °C in a 5% CO_2_ incubator. After 48 h, the medium was replaced with the fresh medium containing stationary-phase promastigotes that were then phagocyted by monocytic cells and transformed into intracellular amastigotes. After 24 h of incubation, newly synthesized compounds were added and the plates were incubated at 37 °C in a 5% CO_2_ incubator for 72 h. After incubation, wells were washed, fixed, and stained with Giemsa. Staining was detected using a Nikon Eclipse E200 light microscope (Nikon, Tokyo, Japan). The infectivity index (% of infected macrophages × average number of amastigotes per macrophage) was determined by counting at least 100 cells in duplicate cultures.

#### Role of an Iron Chelator in the Activation of Compounds^111,112^

To analyze the effect of an iron chelator (DFO or DFP) on the compounds, the late log/stationary phase of promastigotes was seeded with the complete HOMEM medium at 10^6^ cells mL^–1^ in 96-well plates and incubated with the iron chelator for 24 h at 26 °C. Four nontoxic doses of the iron chelator were prepared, in 1:2 dilutions. Each compound was added using a concentration range including the IC_50_ of each compound. Each experiment was performed in duplicate. The viability of the parasites was determined by the alamarBlue assay. The fractional inhibitory concentration (FIC; FIC = IC_50_ of the drug in the combination/IC_50_ of the drug when tested alone) of each compound was calculated and plotted as an isobologram.

#### Evaluation of Intracellular ROS Levels

To monitor the effect of the compounds on the production of ROS in promastigotes of *L. donovani*, an oxidant-sensitive green fluorescent dye (2,7-dichlorofluorescein diacetate, H_2_DCFDA) was used. Promastigotes in their late log/stationary phase were seeded with the complete HOMEM medium at 10^6^ cells mL^–1^ in 96-well plates and incubated with tested compounds at their IC_50_ in a 26 °C incubator for 24 h. Subsequently, parasitic cells were washed in PBS (pH 7.4), loaded with 10 μM permeant probe H_2_DCFDA (Sigma, St. Louis, MO, USA), and incubated in the dark for 35 min at 26 °C.^103^ H_2_O_2_ (20 μM)^124^ and miltefosine (22 μM)^125^ were used as positive controls. Each experiment was performed in triplicate. Reactive oxygen species (ROS) production was measured as an increase in fluorescence caused by the conversion of nonfluorescent dye to highly fluorescent 2,7-dichlorofluorescein, with an excitation wavelength of 488 nm and an emission wavelength of 530 nm. Plates were read by employing a fluorescence microplate reader Varioskan Flash (Thermo Fisher Scientific Inc.).

#### Confocal Microscopy

For imaging, promastigotes of *L. donovani* were seeded (10^6^ cells mL^–1^) on 96-well plates at 26 °C for 72 h. Then, the labeled compounds, at their IC_50_ concentrations, were added and the plate was incubated at 26 °C for 45/50 min. Subsequently, cells were collected, washed with PBS, and immobilized on a slide. TO-PRO-3 (1 μM) (Thermo Fisher Scientific Inc.), a specific dye for nucleic acids,^114,115^ was also added after cell permeabilization, made by incubating promastigotes in methanol/acetone for 10 min at −20 °C. Fluorescence microscopy analysis was performed using a TCS SP2 scanning confocal microscope (Leica Microsystems, Wetzlar, Germany) with a 63× oil objective, the excitation wavelength utilized for the labeled compounds was 488 nm, and emission was detected at 505 nm. The excitation wavelength for TO-PRO-3 was 642 nm, and emission was detected at 661 nm. An antifade agent to reduce photo bleaching was not used.

## Supporting Information Available

X-ray crystallographic analysis, hypothesis on *cis*-stereopreference in tetrahydropyran **9** construction, susceptibility tests on *Leishmania* promastigotes with the modified assay medium, cytotoxicity evaluation in THP-1 cell line, study on the iron chelator (DFO) effect on **2b** and **3b** bioactivity, study on the iron chelator (DFP) effect on **2a** and **3a** bioactivity, calculated log *P*, NMR spectra, HLPC analyses (PDF)
Compound number, SMILES formula, IC_50_ against *L. donovani* promastigotes and amastigotes, CC_50_ against Vero cells and THP-1 cells of compounds **2a**–**2f** and **3a**–**3e** (CSV)

## Abbreviations

CC_50_: half-maximum toxic concentration
SI: selectivity index = CC_50_/IC_50_
R&D: research and development
HSP90: 90 kDa heat shock protein
PG: protective group
CSA: camphorsulfonic acid
equiv: equivalents
nd: not determined
*L*.: *Leishmania*
DFO: desferrioxamine
DFP: deferiprone
FIC: fractional inhibitory concentration = IC_50_ of drug in combination/IC_50_ of drug alone
H_2_DCFDA: 2,7-dichlorodihydrofluorescein diacetate
DCF: dichlorofluorescein
NC: negative control
NBD: nitrobenzyldiazole
TEA: triethylamine
TO-PRO-3: a carbocyanine monomer nucleic acid stain
LRMS: low-resolution mass spectrometry
CyH: cyclohexane
bs: broad singlet
bt: broad triplet
dq: double quartet
FBS: fetal bovine serum
THP-1 cells: human leukemia monocytic cells

## References

[ref1] https://www.who.int/news-room/fact-sheets/detail/leishmaniasis

[ref2] Torres-Guerrero E.; Quintanilla-Cedillo M. R.; Ruiz-Esmenjaud J.; Arenas R. Leishmaniasis: a review. F1000Res. 2017, 6, 750 10.12688/f1000research.11120.1. 28649370 PMC5464238

[ref3] Inceboz T. Epidemiology and Ecology of Leishmaniasis. In Current Topics in Neglected Tropical Diseases; Rodriguez-Morales A. J., Ed.; IntechOpen: London, 2019, 1–15, 10.5772/intechopen.86359.

[ref4] Aronson N. E.; Magill A. J. Leishmaniasis. In Hunter’s Tropical Medicine and Emerging Infectious Diseases; Ryan E. T.; Hill D. R.; Solomon T.; Aronson N.; Endy T. P., Eds.; Elsevier: Amsterdam, 2020; 776–798.

[ref5] Dujardin J.-C.; Campino L.; Cañavate C.; Dedet J.-P.; Gradoni L.; Soteriadou K.; Mazeris A.; Ozbel Y.; Boelaert M. Spread of vector-borne diseases and neglect of Leishmaniasis, Europe. Emerg. Infect. Dis. 2008, 14, 1013–1018. 10.3201/eid1407.071589. 18598618 PMC2600355

[ref6] Alvar J.; Vélez I. D.; Bern C.; Herrero M.; Desjeux P.; Cano J.; Jannin J.; den Boer M.; Leishmaniasis worldwide and global estimates of its incidence. PLoSOne 2012, 7, e35671 10.1371/journal.pone.0035671. PMC3365071 22693548

[ref7] Steverding D. The history of Leishmaniasis. Parasites Vectors 2017, 10, 82 10.1186/s13071-017-2028-5. 28202044 PMC5312593

[ref8] Horrillo L.; Castro A.; Matía B.; Molina L.; García-Martínez J.; Jaqueti J.; García-Arata I.; Carrillo E.; Moreno J.; Ruiz-Giardin J. M.; San Martín J. Clinical aspects of visceral Leishmaniasis caused by *L. infantum* in adults. Ten years of experience of the largest outbreak in Europe: what have we learned?. Parasites Vectors 2019, 12, 359 10.1186/s13071-019-3628-z. 31340851 PMC6657057

[ref9] Koch L. K.; Kochmann J.; Klimpel S.; Cunze S. Modeling the climatic suitability of Leishmaniasis vector species in Europe. Sci. Rep. 2017, 7, 13325 10.1038/s41598-017-13822-1. 29042642 PMC5645347

[ref10] Chalghaf B.; Chemkhi J.; Mayala B.; Harrabi M.; Benie G. B.; Michael E.; Salah A. B. Ecological niche modeling predicting the potential distribution of leishmanial vectors in the Mediterranean basin: impact of climate change. Parasites Vectors 2018, 11, 461 10.1186/s13071-018-3019-x. 30092826 PMC6085715

[ref11] Saliba M.; Shalhoub A.; Taraif S.; Loya A.; Houreih M. A.; El Hajj R.; Khalifeh I. Cutaneous leishmaniasis: an evolving disease with ancient roots. Int. J. Dermatol. 2019, 58, 834–843. 10.1111/ijd.14451. 30968403

[ref12] https://www.who.int/leishmaniasis/en/

[ref13] Gradoni L.; Rogelio L. V.; Mourad M. Manual on case management and surveillance of the leishmaniases in the WHO European Region, 2017; World Health Oragnization: Web site: http://www.euro.who.int/en/publications/abstracts/manual-on-case-management-and-surveillance-of-the-leishmaniases-in-the-who-european-region-2017 (accessed Dec 20, 2019).

[ref14] Barrett M. P.; Croft S. L. Management of trypanosomiasis and leishmaniasis. Br. Med. Bull. 2012, 104, 175–196. 10.1093/bmb/lds031. 23137768 PMC3530408

[ref15] Singh N.; Kumar M.; Singh R. K. Leishmaniasis: current status of available drugs and new potential drug targets. Asian Pac. J. Trop. Med. 2012, 5, 485–497. 10.1016/S1995-7645(12)60084-4. 22575984

[ref16] Uliana S. R. B.; Trinconi C. T.; Coelho A. C. Chemotherapy of leishmaniasis: present challenges. Parasitology 2018, 145, 464–480. 10.1017/S0031182016002523. 28103966

[ref17] Vermeersch M.; da Luz R. I.; Toté K.; Timmermans J.-P.; Cos P.; Maes L. *In vitro* susceptibilities of *Leishmania donovani* promastigote and amastigote stages to antileishmanial reference drugs: practical relevance of stage-specific differences. Antimicrob. Agents Chemother. 2009, 53, 3855–3859. 10.1128/AAC.00548-09. 19546361 PMC2737839

[ref18] Lachaud L.; Bourgeois N.; Plourd M.; Leproho P.; Bastien P.; Ouellette M. Parasite susceptibility to amphotericin B in failures of treatment for visceral leishmaniasis in patients coinfected with HIV type 1 and *Leishmania infantum*. Clin. Infect. Dis. 2009, 48, e16 10.1086/595710. 19093811

[ref19] den Boer M.; Davidson R. N. Treatment options for visceral leishmaniasis. Exp. Rev. Anti-Infect. Ther. 2014, 4, 187–197. 10.1586/14787210.4.2.187. 16597201

[ref20] Rodrigues I. A.; Mazotto A. M.; Cardoso V.; Alves R. L.; Amaral A. C. F.; de Andrade Silva J. R.; Pinheiro A. S.; Vermelho A. B. Natural products: insights into leishmaniasis inflammatory response. Mediators Inflammation 2015, 2015, 1 10.1155/2015/835910. PMC4619978 26538837

[ref21] Croft S. L.; Olliaro P. Leishmaniasis chemotherapy–challenges and opportunities. Clin. Microbiol. Infect. 2011, 17, 1478–1483. 10.1111/j.1469-0691.2011.03630.x. 21933306

[ref22] Ponte-Sucre A.; Gamarro F.; Dujardin J.-C.; Barrett M. P.; López-Vélez R.; García-Hernández R.; Pountain A. W.; Mwenechanya R.; Papadopoulou B. Drug resistance and treatment failure in leishmaniasis: A 21st century challenge. PLoS Neglected Trop. Dis. 2017, 11, e0006052 10.1371/journal.pntd.0006052. PMC5730103 29240765

[ref23] Vanaerschot M.; Huijben S.; Van den Broeck F.; Dujardin J. C. Drug resistance in vectorborne parasites: multiple actors and scenarios for an evolutionary arms race. FEMS Microbiol. Rev. 2014, 38, 41–55. 10.1111/1574-6976.12032. 23815683

[ref24] Hendrickx S.; Boulet G.; Mondelaers A.; Dujardin J. C.; Rijal S.; Lachaud L.; Cos P.; Delputte P.; Maes L. Experimental selection of paromomycin and miltefosine resistance in intracellular amastigotes of *Leishmania donovani* and *L. infantum*. Parasitol. Res. 2014, 113, 1875–1881. 10.1007/s00436-014-3835-7. 24615359

[ref25] Hendrickx S.; Mondelaers A.; Eberhardt E.; Delputte P.; Cos P.; Maes L. *In vivo* selection of paromomycin and miltefosine resistance in *Leishmania donovani* and *L. infantum* in a syrian hamster model. Antimicrob. Agents Chemother. 2015, 59, 4714–4718. 10.1128/AAC.00707-15. 26014955 PMC4505234

[ref26] Mandal S.; Maharjan M.; Singh S.; Chatterjee M.; Madhubala R. Assessing aquaglyceroporin gene status and expression profile in antimony-susceptible and -resistant clinical isolates of *Leishmania donovani* from India. J. Antimicrob. Chemother. 2010, 65, 496–507. 10.1093/jac/dkp468. 20067981

[ref27] Mostafavi M.; Sharifi I.; Farajzadeh S.; Khazaeli P.; Sharifi H.; Pourseyedi E.; Kakooei S.; Bamorovat M.; Keyhani A.; Parizi M. H.; Khosravi A.; Khamesipour A. Niosomal formulation of amphotericin B alone and in combination with glucantime: *in vitro* and *in vivo* leishmanicidal effects. Biomed. Pharmacother. 2019, 116, 108942 10.1016/j.biopha.2019.108942. 31152929

[ref28] Scariot D. B.; Volpato H.; Fernandes N. d. S.; Soares E. F. P.; Ueda-Nakamura T.; Dias-Filho B. P.; Din Z. U.; Rodrigues-Filho E.; Rubira A. F.; Borges O.; Sousa M. D. C.; Nakamura C. V. Activity and cell-death pathway in *Leishmania infantum* induced by sugiol: vectorization using yeast cell wall particles obtained from *Saccharomyces cerevisiae*. Front. Cell. Infect. Microbiol. 2019, 9, 208 10.3389/fcimb.2019.00208. 31259161 PMC6587907

[ref29] Rebello K. M.; Andrade-Neto V. V.; Gomes C. R. B.; de Souza M. V. N.; Branquinha M. H.; Santos A. L. S.; Torres-Santos E. C.; d’Avila-Levy C. M. Miltefosine-lopinavir combination therapy against *Leishmania infantum* infection: *in vitro* and *in vivo* approaches. Front. Cell. Infect. Microbiol. 2019, 9, 229 10.3389/fcimb.2019.00229. 31316919 PMC6611157

[ref30] http://www.who.int/leishmaniasis/research/en/ (accessed Dec 15, 2019). In particular, see:

[ref31] Seifert K. Structures, targets and recent approaches in anti-leishmanial drug discovery and development. Open Med. Chem. J. 2011, 5, 31–39. 10.2174/1874104501105010031. 21629509 PMC3103891

[ref32] Olliaro P.; Darley S.; Laxminarayan R.; Sundar S. Cost-effectiveness projections of single and combination therapies for visceral leishmaniasis in Bihar, India. Trop. Med. Int. Health. 2009, 14, 918–925. 10.1111/j.1365-3156.2009.02306.x. 19563434

[ref33] Srivastava S.; Mishra J.; Gupta A. K.; Singh A.; Shankar P.; Singh S. Laboratory confirmed miltefosine resistant cases of visceral leishmaniasis from India. Parasites Vectors 2017, 10, 49 10.1186/s13071-017-1969-z. 28137296 PMC5282768

[ref34] Garcia-Hernández R.; Manzano J. I.; Castanys S.; Gamarro F. *Leishmania donovani* develops resistance to drug combinations. PLoS Neglected Trop. Dis. 2012, 6, e1974 10.1371/journal.pntd.0001974. PMC3527373 23285310

[ref35] Hendrickx S.; da Luz R. A. I.; Bhandari V.; Kuypers K.; Shaw C. D.; Lonchamp J.; Salotra P.; Carter K.; Sundar S.; Rijal S.; Dujardin J.-C.; Cos P.; Maes L. Experimental induction of paromomycin resistance in antimony-resistant strains of *L. donovani*: outcome dependent on *in vitro* selection protocol. PLoS Neglected Trop. Dis. 2012, 6, e1664 10.1371/journal.pntd.0001664. PMC3362622 22666513

[ref36] Hendrickx S.; Guerin P. J.; Caljon G.; Croft S. L.; Maes L. Evaluating drug resistance in visceral leishmaniasis: the challenges. Parasitology 2018, 145, 453–463. 10.1017/S0031182016002031. 27866478 PMC5989324

[ref37] Collett C. F.; Kitson C.; Baker N.; Steele-Stallard H. B.; Santrot M.-V.; Hutchinson S.; Horn D.; Alsford S. Chemogenomic profiling of anti-leishmanial efficacy and resistance in the related kinetoplastid parasite *Trypanosoma brucei*. Antimicrob. Agents Chemother. 2019, 63, e00795 10.1128/AAC.00795-19. 31160283 PMC6658743

[ref38] http://www.who.int/neglected_diseases/NTD_RoadMap_2012_Fullversion.pdf

[ref39] Burrows J. N.; Elliott R. L.; Kaneko T.; Mowbray C. E.; Waterson D. The role of modern drug discovery in the fight against neglected and tropical diseases. Med. Chem. Commun. 2014, 5, 688–700. 10.1039/c4md00011k.

[ref40] Wyllie S.; Patterson S.; Stojanovski L.; Simeons F. R. C.; Norval S.; Kime R.; Read K. D.; Fairlamb A. H. The anti-trypanosome drug fexinidazole shows potential for treating visceral leishmaniasis. Sci. Transl. Med. 2012, 4, 119re1 10.1126/scitranslmed.3003326. PMC3457684 22301556

[ref41] de Melo Mendes V.; Tempone A. G.; Borborema S. E. T. Antileishmanial activity of H1-antihistamine drugs and cellular alterations in *Leishmania (L.) infantum*. Acta Trop. 2019, 195, 6–14. 10.1016/j.actatropica.2019.04.017. 31002807

[ref42] Miranda-Sapla M. M.; Tomiotto-Pellissier F.; Assolini J. P.; Carloto A. C. M.; da Silva Bortoleti B. T.; Gonçalves M. D.; Tavares E. R.; da Silva Rodrigues J. H.; Simão A. N. C.; Yamauchi L. M.; Nakamura C. V.; Verri W. A. Jr.; Costa I. N.; Conchon-Costa I.; Pavanelli W. R. *Trans*-chalcone modulates *Leishmania amazonensis* infection *in vitro* by Nrf2 overexpression affecting iron availability. Eur. J. Pharmacol. 2019, 853, 275–288. 10.1016/j.ejphar.2019.03.049. 30965057

[ref43] Upadhyay A.; Chandrakar P.; Gupta S.; Parmar N.; Singh S. K.; Rashid M.; Kushwaha P.; Wahajuddin M.; Sashidhara K. V.; Kar S. Synthesis, biological evaluation, structure–activity relationship, and mechanism of action studies of quinoline–metronidazole derivatives against experimental visceral leishmaniasis. J. Med. Chem. 2019, 62, 5655–5671. 10.1021/acs.jmedchem.9b00628. 31124675

[ref44] Otero E.; García E.; Palacios G.; Yepes L. M.; Carda M.; Agut R.; Vélez I. D.; Cardona W. I.; Robledo S. M. Triclosan-caffeic acid hybrids: synthesis, leishmanicidal, trypanocidal and cytotoxic activities. Eur. J. Med. Chem. 2017, 141, 73–83. 10.1016/j.ejmech.2017.09.064. 29028533

[ref45] Baquedano Y.; Moreno E.; Espuelas S.; Nguewa P.; Font M.; Gutierrez K. J.; Jiménez-Ruiz A.; Palop J. A.; Sanmartín C. Novel hybrid selenosulfonamides as potent antileishmanial agents. Eur. J. Med. Chem. 2014, 74, 116–123. 10.1016/j.ejmech.2013.12.030. 24448421

[ref46] Yousuf M.; Mukherjee D.; Dey S.; Pal C.; Adhikari S. Antileishmanial ferrocenylquinoline derivatives: synthesis and biological evaluation against *Leishmania donovani*. Eur. J. Med. Chem. 2016, 124, 468–479. 10.1016/j.ejmech.2016.08.049. 27598235

[ref47] Félix M. B.; de Souza E. R.; de Lima M. d. C. A.; Frade D. K. G.; Serafim V. d. L.; Rodrigues K. A. d. F.; Néris P. L. d. N.; Ribeiro F. F.; Scotti L.; Scotti M. T.; de Aquino T. M.; Mendoça Junior F. J. B.; de Oliveira M. R. Antileishmanial activity of new thiophene-indole hybrids: design, synthesis, biological and cytotoxic evaluation, and chemometric studies. Bioorg. Med. Chem. 2016, 24, 3972–3977. 10.1016/j.bmc.2016.04.057. 27515718

[ref48] Ramu D.; Garg S.; Ayana R.; Keerthana A. K.; Sharma V.; Saini C. P.; Sen S.; Pati S.; Singh S. Novel β-carbolinequinazolinone hybrids disrupt *Leishmania donovani* redox homeostasis and show promising antileishmanial activity. Biochem. Pharmacol. 2017, 129, 26–42. 10.1016/j.bcp.2016.12.012. 28017772

[ref49] Baréa P.; Barbosa V. A.; Bidóia D. L.; de Paula J. C.; Stefanello T. F.; da Costa W. F.; Nakamura C. V.; Sarragiotto M. H. Synthesis, antileishmanial activity and mechanism of action studies of novel β-carboline-1,3,5-triazine hybrids. Eur. J. Med. Chem. 2018, 150, 579–590. 10.1016/j.ejmech.2018.03.014. 29549842

[ref50] Tiuman T. S.; Santos A. O.; Ueda-Nakamura T.; Filho B. P. D.; Nakamura C. V. Recent advances in leishmaniasis treatment. Int. J. Infect. Dis. 2011, 15, e525 10.1016/j.ijid.2011.03.021. 21605997

[ref51] Gil C. Drug Discovery for Leishmaniasis; The Royal Society of Chemistry: London, 2017.

[ref52] Elliott R. L. Third World Diseases; Springer-Verlag: Berlin Heidelberg, 2011.

[ref53] Vijayakumar S.; Das P. Recent progress in drug targets and inhibitors towards combating leishmaniasis. Acta Trop. 2018, 181, 95–104. 10.1016/j.actatropica.2018.02.010. 29452111

[ref54] Negrão F.; Eberlin M. N.; Giorgio S. Proteomic approaches for drug discovery against tegumentary leishmaniasis. Biomed. Pharmacother. 2017, 95, 577–582. 10.1016/j.biopha.2017.08.089. 28869896

[ref55] Sharma M.; Shaikh N.; Yadav S.; Singh S.; Garg P. A systematic reconstruction and constraint-based analysis of *Leishmania donovani* metabolic network: identification of potential antileishmanial drug targets. Mol. BioSyst. 2017, 13, 955–969. 10.1039/C6MB00823B. 28367572

[ref56] Rossi M.; Fasel N. How to master the host immune system? *Leishmania* parasites have the solutions!. Int. Immunol. 2018, 30, 103–111. 10.1093/intimm/dxx075. 29294040 PMC5892169

[ref57] de Oliveira Almeida Petersen A. L.; Campos T. A.; dos Santos Dantas D. A.; de Souza Rebouças J.; da Silva J. C.; de Menezes J. P. B.; Formiga F. R.; de Melo J. V.; Machado G.; Veras P. S. T. Encapsulation of the HSP-90 chaperone inhibitor 17-AAG in stable liposome allow increasing the therapeutic index as assessed, *in vitro*, on *Leishmania (L) amazonensis* amastigotes-hosted in mouse CBA macrophages. Front. Cell. Infect. Microbiol. 2018, 8, 303 10.3389/fcimb.2018.00303. 30214897 PMC6126448

[ref58] Sharma G.; Kar S.; Ball W. B.; Ghosh K.; Das P. K. The curative effect of fucoidan on visceral leishmaniasis is mediated by activation of map kinases through specific protein kinase c isoforms. Cell. Mol. Immunol. 2014, 11, 263–274. 10.1038/cmi.2013.68. 24561457 PMC4085487

[ref59] Kyriazis I. D.; Koutsoni O. S.; Aligiannis N.; Karampetsou K.; Skaltsounis A.-L.; Dotsika E. The leishmanicidal activity of oleuropein is selectively regulated through inflammation- and oxidative stress-related genes. Parasites Vectors 2016, 9, 441 10.1186/s13071-016-1701-4. 27501956 PMC4977900

[ref60] Nirma C.; Rangel G. T.; Alves M. A.; Casanova L. M.; Moreira M. M.; Rodrigues L. M.; Hamerski L.; Tinoco L. W. New *Leishmania donovani* nucleoside hydrolase inhibitors from Brazilian flora. RSC Adv. 2019, 9, 18663–18669. 10.1039/C9RA02382H. PMC9065027 35515226

[ref61] da Silva B. P.; Cortez D. A.; Violin T. V.; Filho B. P. D.; Nakamura C. V.; Ueda-Nakamura T.; Ferreira I. C. P. Antileishmanial activity of a guaianolide from *Tanacetum parthenium* (L.) Schultz Bip. Parasitol. Int. 2010, 59, 643–646. 10.1016/j.parint.2010.08.005. 20732450

[ref62] Correa E.; Cardona D.; Quiñones D.; Torres F.; Franco A. E.; Vélez I. D.; Robledo S.; Echeverri F. Leishmanicidal activity of *Pycnoporus sanguineus*. Phytother. Res. 2006, 20, 497–499. 10.1002/ptr.1890. 16619346

[ref63] Chianese G.; Fattorusso E.; Scala F.; Teta R.; Calcinai B.; Bavestrello G.; Dien H. A.; Kaiser M.; Tasdemir D.; Taglialatela-Scafati O. Manadoperoxides, a new class of potent antitrypanosomal agents of marine origin. Org. Biomol. Chem. 2012, 10, 7197–7207. 10.1039/c2ob26124c. 22859016

[ref64] Monzote L.; Geroldinger G.; Tonner M.; Scull R.; De Sarkar S.; Bergmann S.; Bacher M.; Staniek K.; Chatterjee M.; Rosenau T.; Gille L. Interaction of ascaridole, carvacrol, and caryophyllene oxide from essential oil of *Chenopodium ambrosioides* L. with mitochondria in *Leishmania* and other eukaryotes. Phytother. Res. 2018, 32, 1729–1740. 10.1002/ptr.6097. 29672979 PMC6208284

[ref65] Monzote L.; García M.; Pastor J.; Gil L.; Scull R.; Maes L.; Cos P.; Gille L. Essential oil from *Chenopodium ambrosioides* and main components: activity against *Leishmania*, their mitochondria and other microorganisms. Exp. Parasitol. 2014, 136, 20–26. 10.1016/j.exppara.2013.10.007. 24184772

[ref66] Loo C. S. N.; Lam N. S. K.; Yu D.; Su X.-z.; Lu F. Artemisinin and its derivatives in treating protozoan infections beyond malaria. Pharmacol. Res. 2017, 117, 192–217. 10.1016/j.phrs.2016.11.012. 27867026 PMC5316320

[ref67] Mishina Y. V.; Krishna S.; Haynes R. K.; Meade J. C. Artemisinins inhibit Trypanosoma cruzi and Trypanosoma brucei rhodesiense *in vitro* growth. Antimicrob. Agents Chemother. 2007, 51, 1852–1854. 10.1128/AAC.01544-06. 17339374 PMC1855540

[ref68] WHO World Malaria Report; 2013, ISBN: 9 789241 56469 4.

[ref69] http://whqlibdoc.who.int/publications/2010/9789241547925_eng.pdf

[ref70] Sen R.; Bandyopadhyay S.; Dutta A.; Mandal G.; Ganguly G.; Saha P.; Chatterjee M. Artemisinin triggers induction of cell-cycle arrest and apoptosis in *Leishmania donovani* promastigotes. J. Med. Microbiol. 2007, 56, 1213–1218. 10.1099/jmm.0.47364-0. 17761485

[ref71] Want M. Y.; Islamuddin M.; Chouhan G.; Ozbak H. A.; Hemeg H. A.; Dasgupta A. K.; Chattopadhyay A. P.; Afrin F. Therapeutic efficacy of artemisinin-loaded nanoparticles in experimental visceral leishmaniasis. Colloids Surf., B 2015, 130, 215–221. 10.1016/j.colsurfb.2015.04.013. 25936561

[ref72] Ghaffarifar F.; Heydari F. E.; Dalimi A.; Hassan Z. M.; Delavari M.; Mikaeiloo H. Evaluation of apoptotic and antileishmanial activities of artemisinin on promastigotes and BALB/C mice infected with *leishmania major*. Iran J. Parasitol. 2015, 10, 258–267. 26246824 PMC4522302

[ref73] Mutiso J. M.; Macharia J. C.; Barasa M.; Taracha E.; Bourdichon A. J.; Gicheru M. M. *In vitro* and *in vivo* antileishmanial efficacy of a combination therapy of diminazene and artesunate against *Leishmania donovani* in BALB/c mice. Rev. Inst. Med. Trop. Sao Paulo 2011, 53, 129–132. 10.1590/S0036-46652011000300003. 21755234

[ref74] Chollet C.; Crousse B.; Bories C.; Bonnet-Delpon D.; Loiseau P. M. *In vitro* antileishmanial activity of fluoro-artemisinin derivatives against *Leishmania donovani*. Biomed. Pharmacother. 2008, 62, 462–465. 10.1016/j.biopha.2008.04.003. 18538529

[ref75] Sen R.; Ganguly S.; Saha P.; Chatterjee M. Efficacy of artemisinin in experimental visceral leishmaniasis. Int. J. Antimicrob. Agents 2010, 36, 43–49. 10.1016/j.ijantimicag.2010.03.008. 20403680

[ref76] Want M. Y.; Islammudin M.; Chouhan G.; Ozbak H. A.; Hemeg H. A.; Chattopadhyay A. P.; Afrin F. Nanoliposomal artemisinin for the treatment of murine visceral leishmaniasis. Int. J. Nanomed. 2017, 12, 2189–2204. 10.2147/IJN.S106548. PMC5367595 28356736

[ref77] Krishna S.; Bustamante L.; Haynes R. K.; Staines H. M. Artemisinins: their growing importance in medicine. Trends Pharmacol. Sci. 2008, 29, 520–527. 10.1016/j.tips.2008.07.004. 18752857 PMC2758403

[ref78] *Leishmania* parasites are transmitted into a mammalian host by the bite of phlebotomine sand flies (genus *Phlebotomus* in the Old World and *Lutzomyia* in the New World) as promastigotes (flagellates), which transform into aflagellated amastigotes in host macrophages

[ref79] Cortes S.; Albuquerque A.; Cabral L. I. L.; Lopes L.; Campino L.; Cristiano M. L. S. *In vitro* susceptibility of *Leishmania infantum* to artemisinin derivatives and selected trioxolanes. Antimicrob. Agents Chemother. 2015, 59, 5032–5035. 10.1128/AAC.00298-15. 26014947 PMC4505222

[ref80] Kwofie K. D.; Sato K.; Sanjoba C.; Hino A.; Shimogawara R.; Amoa-Bosompem M.; Ayi I.; Boakye D. A.; Anang A. K.; Chang K.-S.; Ohashi M.; Kim H.-S.; Ohta N.; Matsumoto Y.; Iwanaga S. Oral activity of the antimalarial endoperoxide 6-(1,2,6,7-tetraoxaspiro[7.11]nonadec-4-yl) hexan-1-ol (N-251) against *Leishmania donovani* complex. PLoS Neglected Trop. Dis. 2019, 13, e0007235 10.1371/journal.pntd.0007235. PMC6433226 30908481

[ref81] Persico M.; Quintavalla A.; Rondinelli F.; Trombini C.; Lombardo M.; Fattorusso C.; Azzarito V.; Taramelli D.; Parapini S.; Corbett Y.; Chianese G.; Fattorusso E.; Taglialatela-Scafati O. A new class of antimalarial dioxanes obtained through a simple two-step synthetic approach: rational design and structure–activity relationship studies. J. Med. Chem. 2011, 54, 8526–8540. 10.1021/jm201056j. 22054038

[ref82] Persico M.; Parapini S.; Chianese G.; Fattorusso C.; Lombardo M.; Petrizza L.; Quintavalla A.; Rondinelli F.; Basilico N.; Taramelli D.; Trombini C.; Fattorusso E.; Taglialatela-Scafati O. Further optimization of plakortin pharmacophore: structurally simple 4-oxymethyl-1,2-dioxanes with promising antimalarial activity. Eur. J. Med. Chem. 2013, 70, 875–886. 10.1016/j.ejmech.2013.10.050. 24262380

[ref83] Lombardo M.; Sonawane D. P.; Quintavalla A.; Trombini C.; Dhavale D. D.; Taramelli D.; Corbett Y.; Rondinelli F.; Fattorusso C.; Persico M.; Taglialatela-Scafati O. Optimized synthesis and antimalarial activity of 1,2-dioxane-4-carboxamides. Eur. J. Org. Chem. 2014, 1607–1614. 10.1002/ejoc.201301394.

[ref84] Sonawane D. P.; Persico M.; Corbett Y.; Chianese G.; Di Dato A.; Fattorusso C.; Taglialatela-Scafati O.; Taramelli D.; Trombini C.; Dhavale D. D.; Quintavalla A.; Lombardo M. New antimalarial 3-methoxy-1,2-dioxanes: optimization of cellular pharmacokinetics and pharmacodynamics properties by incorporation of amino and *N*-heterocyclic moieties at C4. RSC Adv. 2015, 5, 72995–73010. 10.1039/C5RA10785G.

[ref85] Sonawane D. P.; Corbett Y.; Dhavale D. D.; Taramelli D.; Trombini C.; Quintavalla A.; Lombardo M. D-glucose-derived 1,2,4-trioxepanes: synthesis, conformational study, and antimalarial activity. Org. Lett. 2015, 17, 4074–4077. 10.1021/acs.orglett.5b01996. 26237035

[ref86] Persico M.; Fattorusso R.; Taglialatela-Scafati O.; Chianese G.; de Paola I.; Zaccaro Z.; Rondinelli F.; Lombardo M.; Quintavalla A.; Trombini C.; Fattorusso E.; Fattorusso C.; Farina B. The interaction of heme with plakortin and a synthetic endoperoxide analogue: new insights into the heme-activated antimalarial mechanism. Sci. Rep. 2017, 7, 45485 10.1038/srep45485. 28383076 PMC5382535

[ref87] Ortalli M.; Varani S.; Rosso C.; Quintavalla A.; Lombardo M.; Trombini C. Evaluation of synthetic substituted 1,2-dioxanes as novel agents against human leishmaniasis. Eur. J. Med. Chem. 2019, 170, 126–140. 10.1016/j.ejmech.2019.02.070. 30878827

[ref88] Robert A.; Dechy-Cabaret O.; Cazelles J.; Meunier B. From mechanistic studies on artemisinin derivatives to new modular antimalarial drugs. Acc. Chem. Res. 2002, 35, 167–174. 10.1021/ar990164o. 11900520

[ref89] Jefford C. W. Why artemisinin and certain synthetic peroxides are potent antimalarials. Implications for the mode of action. Curr. Med. Chem. 2001, 8, 1803–1826. 10.2174/0929867013371608. 11772352

[ref90] Posner G. H.; Oh C. H.; Wang D.; Gerena L.; Milhous W. K.; Meshnick S. R.; Asawamahasadka W. Mechanism-based design, synthesis, and *in vitro* antimalarial testing of new 4-methylated trioxanes structurally related to artemisinin: the importance of a carbon-centered radical for antimalarial activity. J. Med. Chem. 1994, 37, 1256–1258. 10.1021/jm00035a003. 8176702

[ref91] Posner G. H.; Wang D.; Cumming J. N.; Oh C. H.; French A. N.; Bodley A. L.; Shapiro T. A. Further evidence supporting the importance of and the restrictions on a carbon-centered radical for high antimalarial activity of 1,2,4-trioxanes like artemisinin. J. Med. Chem. 1995, 38, 2273–2275. 10.1021/jm00013a001. 7608890

[ref92] Wang J.; Zhang C.-J.; Chia W. N.; Loh C. C. Y.; Li Z.; Lee Y. M.; He Y.; Yuan L.-X.; Lim T. K.; Liu M.; Liew C. X.; Lee Y. Q.; Zhang J.; Lu N.; Lim C. T.; Hua Z.-C.; Liu B.; Shen H.-M.; Tan K. S. W.; Lin Q. Haem-activated promiscuous targeting of artemisinin in *Plasmodium falciparum*. Nat. Commun. 2015, 6, 10111 10.1038/ncomms10111. 26694030 PMC4703832

[ref93] Robert A.; Cazelles J.; Meunier B. Characterization of the alkylation product of heme by the antimalarial drug artemisinin. Angew. Chem., Int. Ed. 2001, 40, 1954–1957. 10.1002/1521-3773(20010518)40:10<1954::AID-ANIE1954>3.0.CO;2-9. 11385684

[ref94] Cazelles J.; Robert A.; Meunier B. Alkylating capacity and reaction products of antimalarial trioxanes after activation by a heme model. J. Org. Chem. 2002, 67, 609–619. 10.1021/jo010688d. 11855997

[ref95] Robert A.; Benoit-Vical F.; Meunier B. The key role of heme to trigger the antimalarial activity of trioxanes. Coord. Chem. Rev. 2005, 249, 1927–1936. 10.1016/j.ccr.2004.12.022.

[ref96] Creek D. J.; Charman W. N.; Chiu F. C. K.; Prankerd R. J.; Dong Y.; Vennerstrom J. L.; Charman S. A. Relationship between antimalarial activity and heme alkylation for spiro- and dispiro-1,2,4-trioxolane antimalarials. Antimicrob. Agents Chemother. 2008, 52, 1291–1296. 10.1128/AAC.01033-07. 18268087 PMC2292547

[ref97] Taglialatela-Scafati O.; Fattorusso E.; Romano A.; Scala F.; Barone V.; Cimino P.; Stendardo E.; Catalanotti B.; Persico M.; Fattorusso C. Insight into the mechanism of action of plakortins, simple 1,2-dioxane antimalarials. Org. Biomol. Chem. 2010, 8, 846–856. 10.1039/B918600J. 20135043

[ref98] O’Neill P. M.; Barton V. E.; Ward S. A. The molecular mechanism of action of artemisinin - the debate continues. Molecules 2010, 15, 1705–1721. 10.3390/molecules15031705. 20336009 PMC6257357

[ref99] Tilley L.; Straimer J.; Gnädig N. F.; Ralph S. A.; Fidock D. A. Artemisinin action and resistance in *Plasmodium falciparum*. Trends Parasitol. 2016, 32, 682–696. 10.1016/j.pt.2016.05.010. 27289273 PMC5007624

[ref100] Stocks P. A.; Bray P. G.; Barton V. E.; Al-Helal M.; Jones M.; Araujo N. C.; Gibbons P.; Ward S. A.; Hughes R. H.; Biagini G. A.; Davies J.; Amewu R.; Mercer A. E.; Ellis G.; O’Neill P. M. Evidence for a common non-heme chelatable-iron-dependent activation mechanism for semisynthetic and synthetic endoperoxide antimalarial drugs. Angew. Chem., Int. Ed. 2007, 46, 6278–6283. 10.1002/anie.200604697. 17640025

[ref101] Geroldinger G.; Tonner M.; Fudickar W.; De Sarkar S.; Dighal A.; Monzote L.; Staniek K.; Linker T.; Chatterjee M.; Gille L. Activation of anthracene endoperoxides in *Leishmania* and impairment of mitochondrial functions. Molecules 2018, 23, 1680 10.3390/molecules23071680. PMC6100073 29996524

[ref102] Sen R.; Saha P.; Sarkar A.; Ganguly S.; Chatterjee M. Iron enhances generation of free radicals by artemisinin causing a caspase-independent, apoptotic death in *Leishmania donovani* promastigotes. Free Radical Res. 2010, 44, 1289–1295. 10.3109/10715762.2010.498475. 20815780

[ref103] De Sarkar S.; Sarkar D.; Sarkar A.; Dighal A.; Chakrabarti S.; Staniek K.; Gille L.; Chatterjee M. The leishmanicidal activity of artemisinin is mediated by cleavage of the endoperoxide bridge and mitochondrial dysfunction. Parasitology 2019, 146, 511–520. 10.1017/S003118201800183X. 30392476

[ref104] Geroldinger G.; Tonner M.; Hettegger H.; Bacher M.; Monzote L.; Walter M.; Staniek K.; Rosenau T.; Gille L. Mechanism of ascaridole activation in *Leishmania*. Biochem. Pharmacol. 2017, 132, 48–62. 10.1016/j.bcp.2017.02.023. 28263719

[ref105] Rideau E.; Mäsing F.; Fletcher S. P. Asymmetric conjugate addition of alkylzirconocenes to cyclopent-4-ene-1,3-dione monoacetals. Synthesis 2015, 47, 2217–2222. 10.1055/s-0034-1379928.

[ref106] Hong R.; Chen Y.; Deng L. Catalytic enantioselective total syntheses of bisorbicillinolide, bisorbicillinol, and bisorbibutenolide. Angew. Chem. 2005, 44, 3478–3481. 10.1002/anie.200500480. 15861438

[ref107] Prasad K. R.; Anbarasan P. Enantioselective synthesis of α-benzyloxy-ω-alkenals: application to the synthesis of (+)-*exo*-brevicomin, (+)-iso-*exo*-brevicomin, and (−)-isolaurepan. Tetrahedron: Asymmetry 2007, 18, 1419–1427. 10.1016/j.tetasy.2007.05.014.

[ref108] Access Structures; Crystallographic data of compound 9b have been deposited with the Cambridge Crystallographic Data Centre (CCDC) as supplementary publication number CCDC 1972697. Copies of the data can be obtained free of charge via www.ccdc.cam.ac.uk/getstructures.

[ref109] NMR experiments (nOe) on the two isomers of olefin **8a** were used to establish the double bond geometry (see the Supporting Information for details). The results were extended to olefin **8c**.

[ref110] The favored *cis* relative stereochemistry established by X-ray crystallographic analysis for product **9b** was extended to the other tetrahydropyrans **9a** and **9c** obtained using the same stereoselective synthetic approach. For a hypothesis justifying the *cis*-stereopreference in the construction of tetrahydropyrans **9**, see the Supporting Information.

[ref111] Bell A. Antimalarial drug synergism and antagonism: mechanistic and clinical significance. FEMS Microbiol. Lett. 2005, 253, 171–184. 10.1016/j.femsle.2005.09.035. 16243458

[ref112] Benoit-Vical F.; Robert A.; Meunier B. *In vitro* and *in vivo* potentiation of artemisinin and synthetic endoperoxide antimalarial drugs by metalloporphyrins. Antimicrob. Agents Chemother. 2000, 2836–2841. 10.1128/AAC.44.10.2836-2841.2000. 10991867 PMC90158

[ref113] See ref (100) and references cited therein

[ref114] Milani L.; Maurizii M. G. Vasa expression in spermatogenic cells during the reproductive-cycle phases of *Podarcis sicula* (Reptilia, Lacertidae). J. Exp. Zool., Part B 2015, 324, 424–434. 10.1002/jez.b.22628. 25944282

[ref115] Milani L.; Pecci A.; Ghiselli F.; Passamonti M.; Bettini S.; Franceschini V.; Maurizii M. G. VASA expression suggests shared germ line dynamics in bivalve molluscs. Histochem. Cell Biol. 2017, 148, 157–171. 10.1007/s00418-017-1560-x. 28386635 PMC5508042

[ref116] Das N. K.; Biswas S.; Solanki S.; Mukhopadhyay C. K. *Leishmania donovani* depletes labile iron pool to exploit iron uptake capacity of macrophage for its intracellular growth. Cell. Microbiol. 2009, 11, 83–94. 10.1111/j.1462-5822.2008.01241.x. 18823384 PMC2774478

[ref117] Zaidi A.; Singh K. P.; Ali V. *Leishmania* and its quest for iron: an update and overview. Mol. Biochem. Parasitol. 2017, 211, 15–25. 10.1016/j.molbiopara.2016.12.004. 27988301

[ref118] Sarkar A.; Andrews N. W.; Laranjeira-Silva M. F. Intracellular iron availability modulates the requirement for *Leishmania* Iron Regulator 1 (LIR1) during macrophage infections. Int. J. Parasitol. 2019, 49, 423–427. 10.1016/j.ijpara.2019.02.002. 30910463 PMC6527483

[ref119] Sato T.; Onuma T.; Nakamura I.; Terada M. Platinum-catalyzed cycloisomerization of 1,4-enynes via 1,2-alkenyl rearrangement. Org. Lett. 2011, 13, 4992–4995. 10.1021/ol202104c. 21913660

[ref120] Ishikawa Y.; Yamashita A.; Uno T. Efficient photocleavage of DNA by cationic porphyrin-acridine hybrids with the effective length of diamino alkyl linkage. Chem. Pharm. Bull. 2001, 49, 287–293. 10.1248/cpb.49.287. 11253918

[ref121] Shi M.; Shen Y.-M. Transition-metal-catalyzed reactions of propargylamine with carbon dioxide and carbon disulfide. J. Org. Chem. 2002, 67, 16–21. 10.1021/jo0014966. 11777433

[ref122] Yang Y.; Wang J.; Kayser M. Approaches to the synthesis of enantiopure α-hydroxy-β-lactams with functionalized side chains. Tetrahedron: Asymmetry 2007, 18, 2021–2029. 10.1016/j.tetasy.2007.08.012.

[ref123] Silva A. M.; Cordeiro-da-Silva A.; Coombs G. H. Metabolic variation during development in culture of *Leishmania donovani* promastigotes. PLoS Neglected Trop. Dis. 2011, 5, e1451 10.1371/journal.pntd.0001451. PMC3243725 22206037

[ref124] Ahmad B.; Islam A.; Khan A.; Khan M. A.; ul Haq I.; Jafri L.; Ahmad M.; Mehwish S.; Khan A.; Ullah N. Comprehensive investigations on anti-leishmanial potentials of *Euphorbia wallichii* root extract and its effects on membrane permeability and apoptosis. Comp. Immunol., Microbiol. Infect. Dis. 2019, 64, 138–145. 10.1016/j.cimid.2019.03.007. 31174688

[ref125] Stroppa P. H. F.; Antinarelli L. M. R.; Carmo A. M. L.; Gameiro J.; Coimbra E. S.; da Silva A. D. Effect of 1,2,3-triazole salts, non-classical bioisosteres of miltefosine, on *Leishmania amazonensis*. Bioorg. Med. Chem. 2017, 25, 3034–3045. 10.1016/j.bmc.2017.03.051. 28433512

