# Supplementary material for: Evaluation of the Pharmacophoric Role of the O–O Bond in Synthetic Antileishmanial Compounds: Comparison between 1,2-Dioxanes and Tetrahydropyrans
Source: J Med Chem. 2020 Oct 22;63(21):13140–58. doi: 10.1021/acs.jmedchem.0c01589 (PMC8018184; doi:10.1021/acs.jmedchem.0c01589)
Supplement: Supplementary file 1 — jm0c01589_si_001.pdf [file jm0c01589_si_001.pdf]

Supporting Information

Evaluation of the pharmacophoric role of the O-O bond in synthetic anti-leishmanial compounds: comparison between 1,2-dioxanes and tetrahydropyrans

Margherita Ortalli,[a] Stefania Varani,[a,b] Giorgia Cimato,[a] Ruben Veronesi,[c] Arianna Quintavalla,*[c,d] Marco Lombardo,*[c,d] Magda Monari,[c] Claudio Trombini[c,d]

[a] Unit of Clinical Microbiology, Regional Reference Centre for Microbiological Emergencies (CRREM), St. Orsola-Malpighi University Hospital, Via Massarenti 9, 40138 Bologna, Italy.

[b] Alma Mater Studiorum - University of Bologna, Department of Experimental, Diagnostic and Specialty Medicine, Via Massarenti 9, 40138 Bologna, Italy.

[c] Alma Mater Studiorum - University of Bologna, Department of Chemistry "G. Ciamician", Via Selmi 2, 40126 Bologna, Italy.

[d] Centro Interuniversitario di Ricerca sulla Malaria (CIRM) - Italian Malaria Network (IMN), University of Milan, 20100 Milan, Italy.

Corresponding Authors:

*E-mail: arianna.quintavalla@unibo.it

*E-mail: marco.lombardo@unibo.it

List of contents:

X-ray crystallography	S3
Cis-stereopreference in tetrahydropyrans **9**	S5
Susceptibility test on *Leishmania* promastigotes with 10% FBS in the HOMEM assay medium	S6
Cytotoxicity in THP-1 cell line	S7
Effect of iron chelator DFO on bioactivity of **2b** and **3b**	S8
Effect of iron chelator DFP on bioactivity of **2a** and **3a**	S10
Calculated LogP	S12
NMR spectra	S17
HLPC analyses	S45
References	S52

X-ray crystallography

The X-ray intensity data were measured on a Bruker Apex II CCD diffractometer. Cell dimensions, and the orientation matrix were initially determined from a least-squares refinement on reflections measured in three sets of 20 exposures, collected in three different ω regions, and eventually refined against all data. A full sphere of reciprocal space was scanned by 0.3° ω steps. The software SMART[1] was used for collecting frames of data, indexing reflections and determination of lattice parameters. The collected frames were then processed for integration by the SAINT program,[1] and an empirical absorption correction was applied using SADABS.[2] The structures were solved by direct methods (SIR 2014)[3] and subsequent Fourier syntheses and refined by full-matrix least-squares on F^2 (SHELXTL),[4] using anisotropic thermal parameters for all non-hydrogen atoms. The aromatic, methylene, methine and methyl hydrogen atoms were placed in calculated positions, refined with isotropic thermal parameters $U(H) = 1.2\ Ueq(C)$ or $U(H) = 1.5\ Ueq(C_{methyl})$ and allowed to ride on their carrier carbons. Molecular drawings were generated using Mercury.[5]

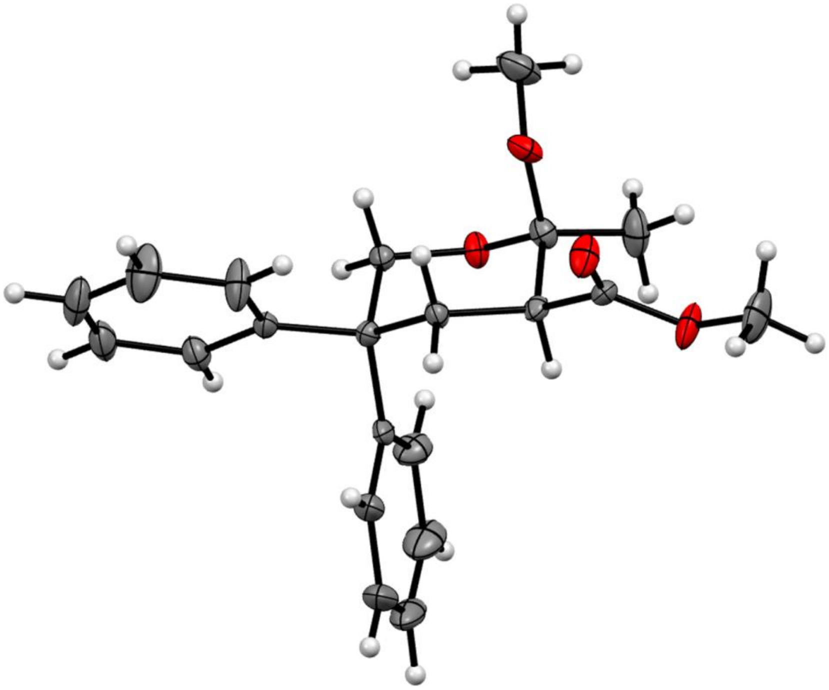

Figure S1. Determination of the relative stereochemistry of 2-methoxy tetrahydropyran **9b** through X-ray crystallographic analysis (thermal ellipsoids are drawn at 30% of the probability level).

Table S1. Crystal data and structure refinement for compound **9b**.

Compound	**9b**
Formula	$C_{21}H_{24}O_4$
Fw	340.40
T, K	296
λ, Å	0.71073
Crystal symmetry	Monoclinic
Space group	$P2_1/c$
a, Å	7.8520(7)
b, Å	19.4333(19)
c, Å	12.8156(13)
α	90
β	106.256(3)
γ	90
Cell volume, Å^3	1877.4(3)
Z	4
D_c, Mg m^{-3}	1.204
μ(Mo-K_α), mm^{-1}	0.082
F(000)	728
Crystal size/ mm	0.20 x 0.15 x 0.10
θ limits, °	1.959 - 24.999
Reflections collected	16176
Unique obs. Reflections [$F_o > 4\sigma(F_o)$]	3143 [R(int) = 0.1289]
Goodness-of-fit-on F^2	1.072
R_1 (F)[a], wR_2 (F^2)[b] [$I > 2\sigma(I)$]	0.0978, 0.2296
Largest diff. peak and hole, e. Å^{-3}	0.287 and -0.378

[a] $R_1 = \Sigma||F_o|-|F_c||/\Sigma|F_o|$.

[b] $wR_2 = [\Sigma w(F_o^2-F_c^2)^2/\Sigma w(F_o^2)^2]^{1/2}$ where $w = 1/[\sigma^2(F_o^2) + (aP)^2+ bP]$ where $P = (F_o^2 + F_c^2)/3$.

Cis-stereopreference in tetrahydropyrans 9

The favored *cis* relative stereochemistry established by X-ray crystallographic analysis for product **9b** was extended to the other tetrahydropyrans **9a** and **9c** obtained using the same stereoselective synthetic approach (Scheme S1).

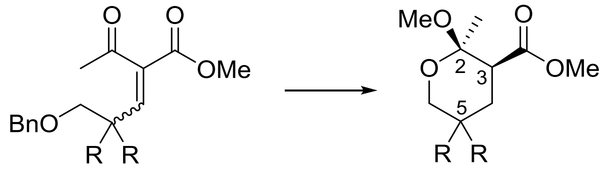

Scheme S1. Reagents and conditions: Pd/C (20% w/w), H_2 (filled balloon), MeOH, rt, 12-16 h.

In Scheme S2 we present a hypothesis which could explain the *cis*-stereopreference in the construction of tetrahydropyrans **9**.

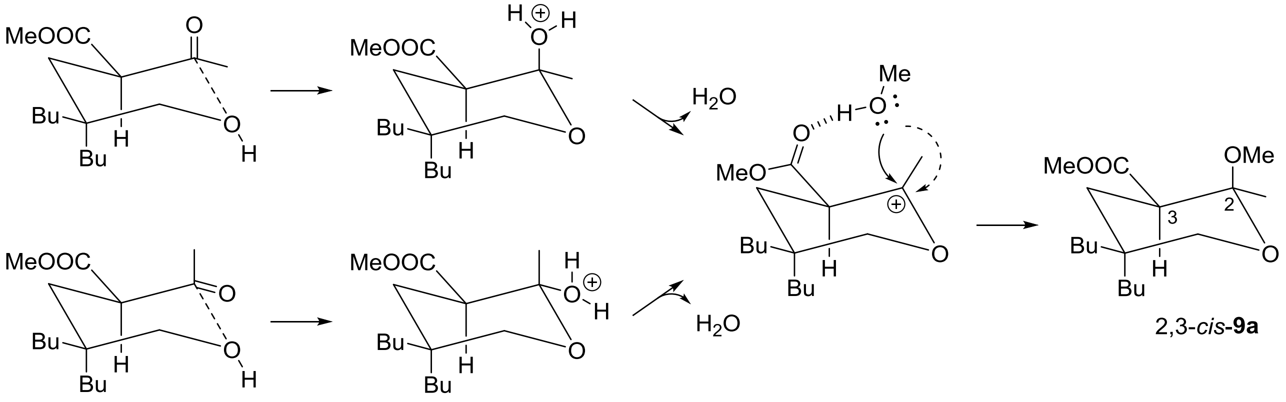

Scheme S2. Hypothesis justifying the *cis*-stereoselective synthesis of tetrahydropyran **9a**.

Susceptibility test on *Leishmania* promastigotes with 10% FBS in the HOMEM assay medium

As the 20% FBS that is employed in the assay medium is a very nutrient- and antioxidant-rich environment, which could potentially prevent a stronger activation of endoperoxides **2** in comparison to the non-peroxidic analogs **3**, we performed additional susceptibility tests for selected compounds by employing *L. donovani* cultures in HOMEM 10% FBS and we compared the results with those obtained by employing 20% FBS.

We observed a slight reduction of the IC_{50} of all the tested compounds with 10% FBS in the assay medium as compared to 20% FBS. However, the bioactivities of endoperoxides **2** and tetrahydropyrans **3** remained comparable, suggesting a not significant pharmacophoric role of the O-O bond also under these bioassay-conditions. The antioxidant-rich environment (ie 20% FBS) does not appear to play a significant role in blocking the activation of endoperoxides. We can speculate that the increased antileishmanial activity of both peroxides and tetrahydropyrans in the presence of 10% FBS can be caused by a decreased parasite vitality with lower FBS concentration.

Table S2. Inhibitory activity of tetrahydropyrans **3** and endoperoxides **2** against promastigotes of *L. donovani* in the presence of 20% FBS and 10% FBS, respectively, in the medium.

Compound	IC_{50} 20% FBS	IC_{50} 10% FBS
**3a**	3.4	1.9
**2a**	7.5	2.7
**3b**	5.8	3.2
**2b**	6.3	1.2

Cytotoxicity in THP-1 cell line

We performed additional cytotoxicity tests employing THP-1 cell line. We also calculated the corresponding selectivity indexes, as the ratio between CC_{50} of THP-1 cell line and IC_{50} on amastigotes of *L. donovani*.

We found that three compounds **3a**, **3d** and **2d** showed a slightly higher cytotoxic effect on THP-1 cells than on VERO cells. On the other hand, compounds **2a**, **3b** and **2b** showed a lower cytotoxic effect on THP-1 cells than on VERO cells; thus showing a not significant variance of selectivity index.

Table S3. Inhibitory activity of tetrahydropyrans **3** and endoperoxides **2** against amastigotes of *L. donovani*, cytotoxicity in human acute monocytic leukemia cell line (THP-1) and selectivity index (SI).

**Tetrahydropyran 3[a]**	**IC_{50} (μM)[b]**	**CC_{50} (μM)[c]**	**SI[d]**	**Endoperoxide 2[a]**	**IC_{50} (μM)[b]**	**CC_{50} (μM)[c]**	**SI[d]**
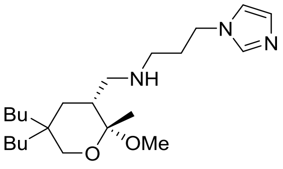 **3a**	3.4 ±0.6	26.0	7.6	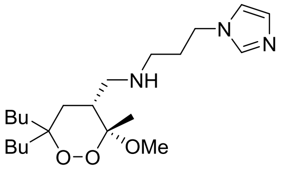 **2a**	12.2 ±1.2	86.0	7.0
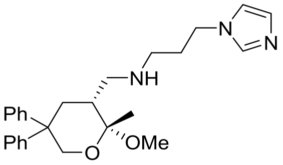 **3b**	3.2 ±0.9	50.0	15.6	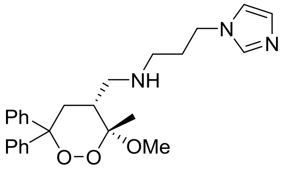 **2b**	5.0 ±1.0	52.5	10.5
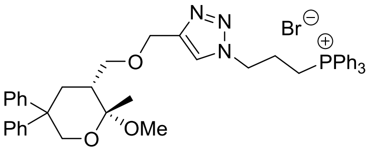 **3d**	2.8 ±0.7	64.1	22.9	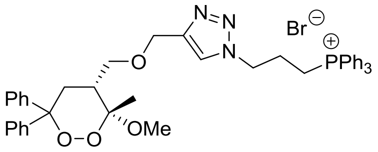 **2d**	16.5 ±1.5	142.5	8.6

[a] Compounds tested as racemates. [b] IC_{50} represents the concentration of a compound that causes 50% growth inhibition. Results represent the mean (± standard deviation, SD) of three independent experiments performed in duplicate. [c] CC_{50} represents 50% cytotoxic concentration on THP-1 cells. [d] Selectivity index (SI) = CC_{50}/IC_{50}.

Effect of iron chelator DFO on bioactivity of 2b and 3b

As for compounds **2a** and **3a,** we evaluated the bioactivity of tetrahydropyran **3b** and endoperoxide **2b** against promastigotes of *L. donovani* in the presence of the iron chelator DFO to investigate whether the IC_{50} values of these compounds were affected by the presence of DFO. The investigation was performed by co-incubating **2b** and **3b** with DFO at different concentrations (100 μM, 50 μM, 25 μM, 15 μM). Table S4 shows how the different doses of DFO don't adduct visible variation of IC_{50} of the compounds, thus confirming a scarce influence of the iron on the compound bioactivity, confirming the same behavior observed for **2a** and **3a**.

Table S4. Inhibitory activity of tetrahydropyran **3b** and endoperoxide **2b** against promastigotes of *L. donovani* in the presence or absence of the iron chelator DFO.

Compound[a]	DFO (μM)	IC_{50} (μM)[b]
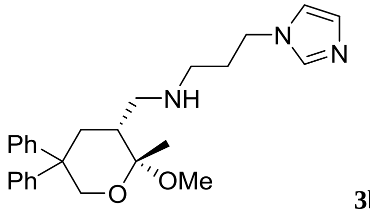 **3b**	-	6.6
	100	4.9
	50	6.6
	25	7.5
	15	7.5
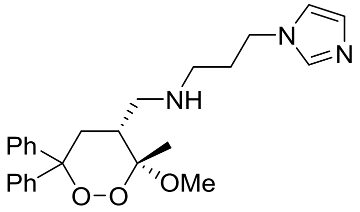 **2b**	-	7.5
	100	5.1
	50	5.5
	25	5.3
	15	6.0

[a] Compounds tested as racemates. [b] IC_{50} represents the concentration of a compound that causes 50% growth inhibition. DFO = desferrioxamine.

The isobole technique depicts synergistic (FIC values < 0.5) or antagonistic (FIC values > 4.0) interactions between DFO and the two tested compounds. Has shown in Figure S2, combination of DFO with **2b** or **3b** resulted in additive effects, since no antagonism or synergism was observed. The same trend of compounds **2a** and **3a** was confirmed.

A)

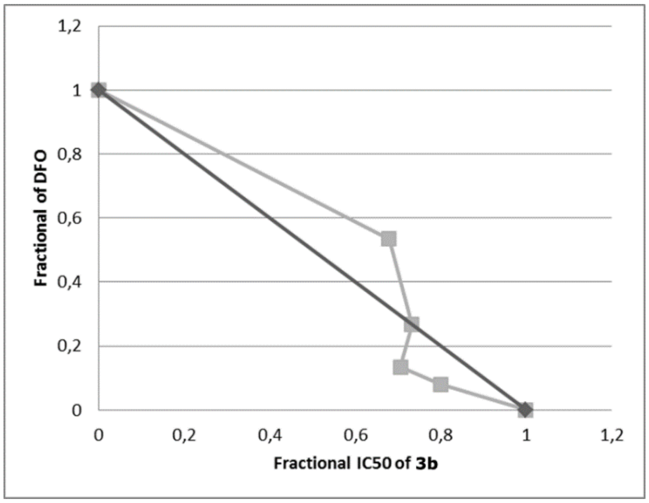

B)

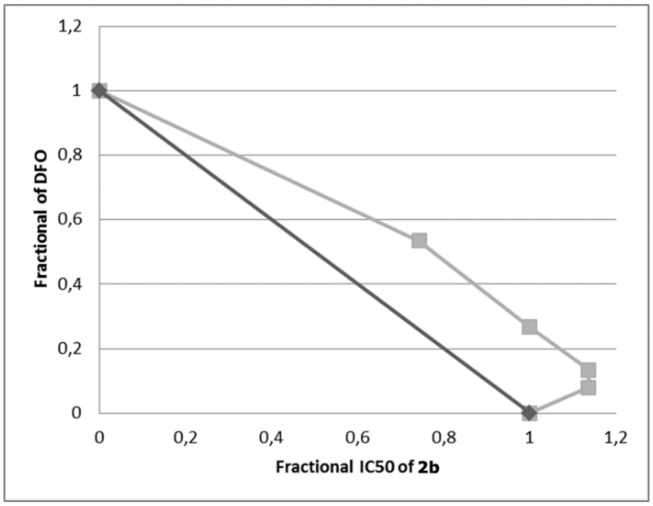

Figure S2. Isobolograms depicting the interaction of: **A**) tetrahydropyran **3b** with iron chelator DFO; **B**) endoperoxide **2b** with iron chelator DFO. Dark grey line is line of additivity. X axes depict the fractional inhibitory concentration (FIC; FIC = IC_{50} of the drug in the combination/IC_{50} of the drug when tested alone). Y axes depict the fractional of DFO. Square in the figure indicates Σ FIC values from each drug combination.

Effect of the iron chelator DFP on bioactivity of 2a and 3a

To investigate the role of iron in the activation of compounds **2a** and **3a**, we also tested these compounds against promastigotes of *L. donovani* in the presence of the iron chelator Deferiprone (DFP), which is a more lipophilic iron-chelator than DFO.
IC_{50} (DFP) = 219 μM; CC_{50} (DFP) = 143 μM.

Table S5. Inhibitory activity of tetrahydropyran **3a** and endoperoxide **2a** against promastigotes of *L. donovani* in the presence or absence of the iron chelator DFP.

Compound[a]	DFP (μM)	IC_{50} (μM)[b]
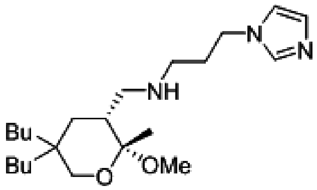 **3a**	-	3.4
	200	4
	100	6.2
	50	6.5
	25	6.1
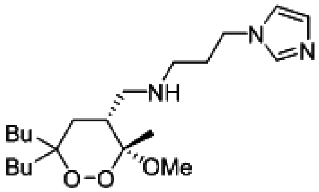 **2a**	-	7.5
	200	7.1
	100	15
	50	13.2
	25	13

[a] Compounds tested as racemates. [b] IC_{50} represents the concentration of a compound that causes 50% growth inhibition. DFP = deferiprone.

Also in this case, we observed a not significant variation of the IC_{50} values when the concentration of the iron-chelator was modified. At the highest concentration of the iron-chelator, the lowest IC_{50} was recorded, confirming the activity of these compounds even in the absence of low molecular weight iron-species.

As shown in isobolograms depicted in Figure S3, we observed a not significant variation of the IC_{50} values when the concentration of the iron-chelator increased. FIC values for both compounds are > 0.5 and < 4, therefore no antagonism or synergism was observed.

A) **B)**

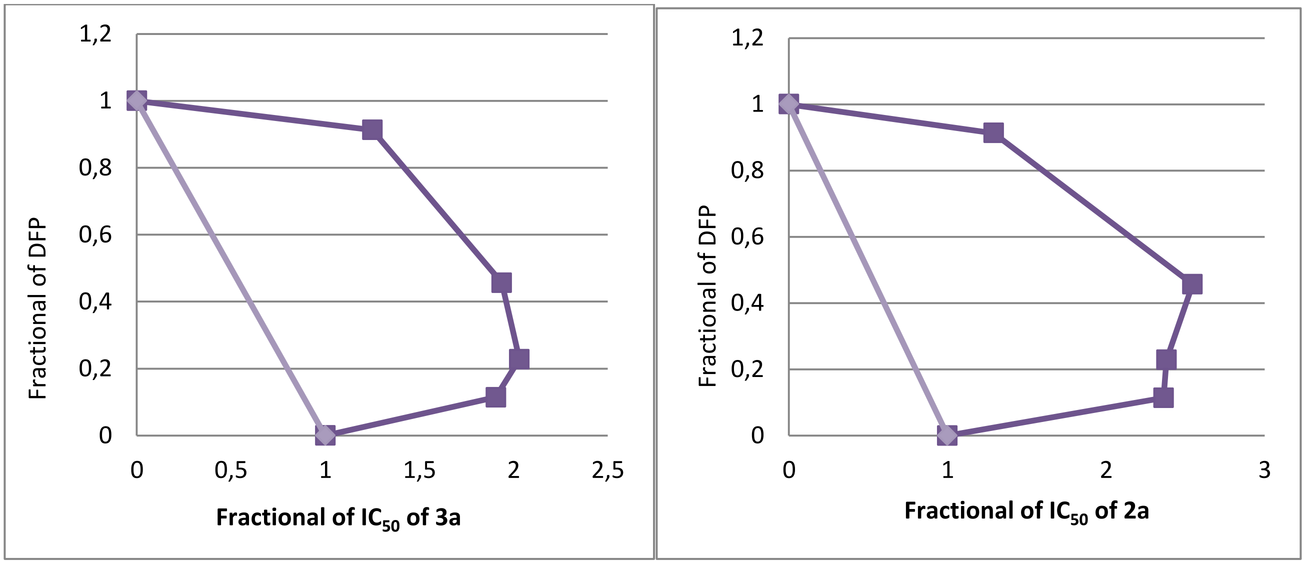

Figure S3 Isobolograms depicting the interaction of: **A**) tetrahydropyran **3a** with iron chelator DFP; **B**) endoperoxide **2a** with iron chelator DFP. Light purple line is line of additivity. X axes depict the fractional inhibitory concentration (FIC; FIC = IC_{50} of the drug in the combination/IC_{50} of the drug when tested alone). Y axes depict the fractional of DFP. Square in the figure indicates Σ FIC values from each drug combination.

Calculated LogP

The LogP values of the compounds tested on *L. donovani* promastigotes were calculated using the software ChemDraw Professional 15.0.

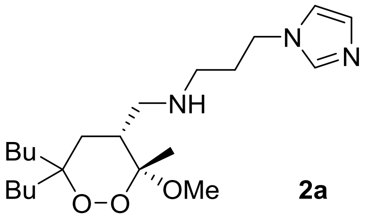

2a

tPSA: 55.32

CLogP: 4.2224

CMR: 11.033

LogS: -4.823

pKa: 8.677

```
*************************************************************************
 Estimation of logarithm of Partition Coefficient [n-Octanol/Water] Log(p)
*************************************************************************

Log(p)........:     3.48
St..deviation.:     0.47
by Crippen's fragmentation: J.Chem.Inf.Comput.Sci.,27,21(1987).

Log(p)........:     3.38
St..deviation.:     0.49
by Viswanadhan's fragmentation: J.Chem.Inf.Comput.Sci.,29,163(1989).
```

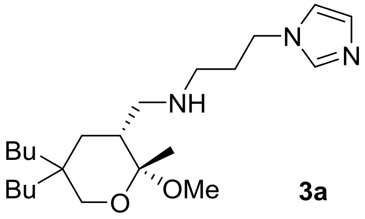

3a

tPSA: 46.09

CLogP: 4.5434

CMR: 11.2167

LogS: -5.023

pKa: 9.165

```
*************************************************************************
 Estimation of logarithm of Partition Coefficient [n-Octanol/Water] Log(p)
*************************************************************************

Log(p)........:     3.81
St..deviation.:     0.47
by Crippen's fragmentation: J.Chem.Inf.Comput.Sci.,27,21(1987).

Log(p)........:     3.68
St..deviation.:     0.49
by Viswanadhan's fragmentation: J.Chem.Inf.Comput.Sci.,29,163(1989).
```

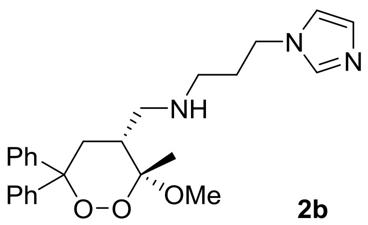

2b

tPSA: 55.32

CLogP: 3.1264

CMR: 12.345

LogS: -5.604

pKa: 8.584

```
**************************************************************************
 Estimation of logarithm of Partition Coefficient [n-Octanol/Water] Log(p)
**************************************************************************

Log(p)........:     3.63
St..deviation.:     0.47
by Crippen's fragmentation: J.Chem.Inf.Comput.Sci.,27,21(1987).

Log(p)........:     3.73
St..deviation.:     0.49
by Viswanadhan's fragmentation: J.Chem.Inf.Comput.Sci.,29,163(1989).
```

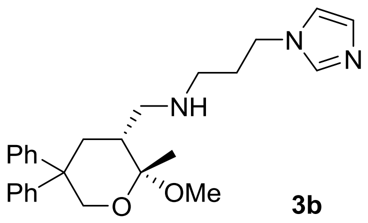

3b

tPSA: 46.09

CLogP: 3.4474

CMR: 12.5287

LogS: -5.546

pKa: 9.079

```
**************************************************************************
 Estimation of logarithm of Partition Coefficient [n-Octanol/Water] Log(p)
**************************************************************************

Log(p)........:     3.69
St..deviation.:     0.47
by Crippen's fragmentation: J.Chem.Inf.Comput.Sci.,27,21(1987).

Log(p)........:     3.74
St..deviation.:     0.49
by Viswanadhan's fragmentation: J.Chem.Inf.Comput.Sci.,29,163(1989).
```

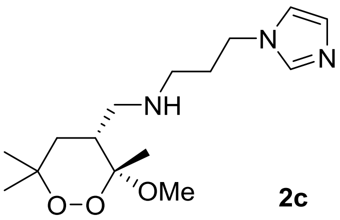

2c

tPSA: 55.32

CLogP: 1.0484

CMR: 8.2502

LogS: -2.624

pKa: 8.669

```
************************************************************************
 Estimation of logarithm of Partition Coefficient [n-Octanol/Water] Log(p)
************************************************************************

Log(p)........:     0.84
St..deviation.:     0.47
by Crippen's fragmentation: J.Chem.Inf.Comput.Sci.,27,21(1987).

Log(p)........:     0.86
St..deviation.:     0.49
by Viswanadhan's fragmentation: J.Chem.Inf.Comput.Sci.,29,163(1989).
```

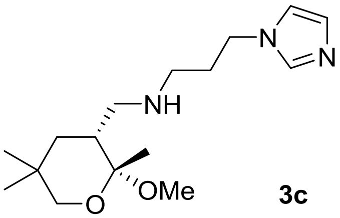

3c

tPSA: 46.09

CLogP: 1.3694

CMR: 8.4339

LogS: -2.521

pKa: 9.157

```
************************************************************************
 Estimation of logarithm of Partition Coefficient [n-Octanol/Water] Log(p)
************************************************************************

Log(p)........:     1.31
St..deviation.:     0.47
by Crippen's fragmentation: J.Chem.Inf.Comput.Sci.,27,21(1987).

Log(p)........:     1.30
St..deviation.:     0.49
by Viswanadhan's fragmentation: J.Chem.Inf.Comput.Sci.,29,163(1989).
```

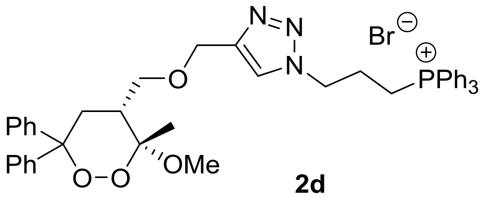

2d

tPSA: 64.88

CLogP: 8.7696

CMR: 20.95

LogS: -11.27

pKa: N/A

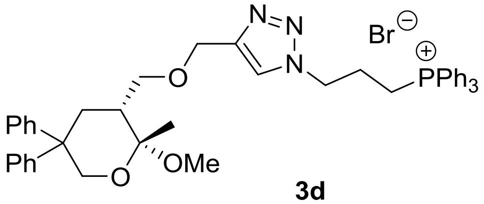

3d

tPSA: 55.65

CLogP: 9.0906

CMR: 21.1337

LogS: -11.2

pKa: N/A

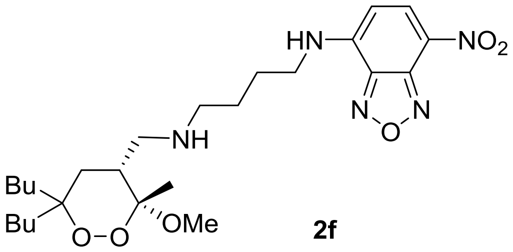

2f

tPSA: 137.51

CLogP: 6.3691

CMR: 13.7383

LogS: -7.214

pKa: 9.039

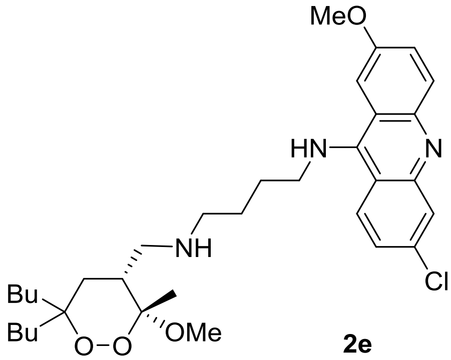

2e

tPSA: 73.34

CLogP: 9.25217

CMR: 16.9203

LogS: -9.049

pKa: 9.029

```
*************************************************************************
 Estimation of logarithm of Partition Coefficient [n-Octanol/Water] Log(p)
*************************************************************************

Log(p)........:     7.83
St..deviation.:     0.47
by Crippen's fragmentation: J.Chem.Inf.Comput.Sci.,27,21(1987).

Log(p)........:     7.68
St..deviation.:     0.49
by Viswanadhan's fragmentation: J.Chem.Inf.Comput.Sci.,29,163(1989).
```

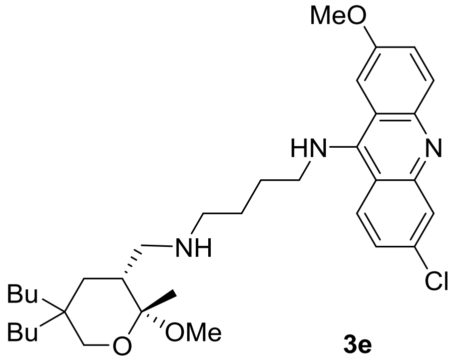

3e

tPSA: 64.11

CLogP: 9.57317

CMR: 17.104

LogS: -9.212

pKa: 9.518

```
*************************************************************************
 Estimation of logarithm of Partition Coefficient [n-Octanol/Water] Log(p)
*************************************************************************

Log(p)........:     8.16
St..deviation.:     0.47
by Crippen's fragmentation: J.Chem.Inf.Comput.Sci.,27,21(1987).

Log(p)........:     7.97
St..deviation.:     0.49
by Viswanadhan's fragmentation: J.Chem.Inf.Comput.Sci.,29,163(1989).
```

NMR spectra

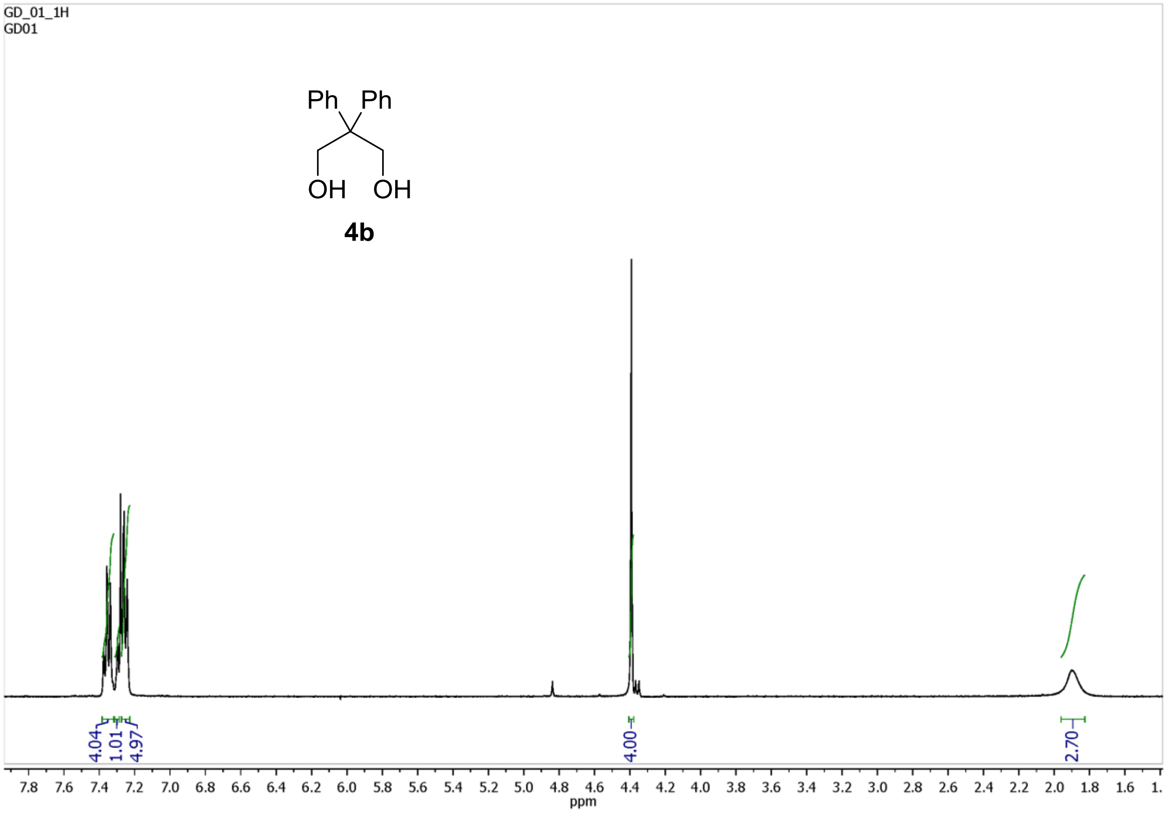

GD_01_1H
GD01
Ph Ph
OH OH
4b
4.04
1.01
4.97
4.00
2.70
ppm

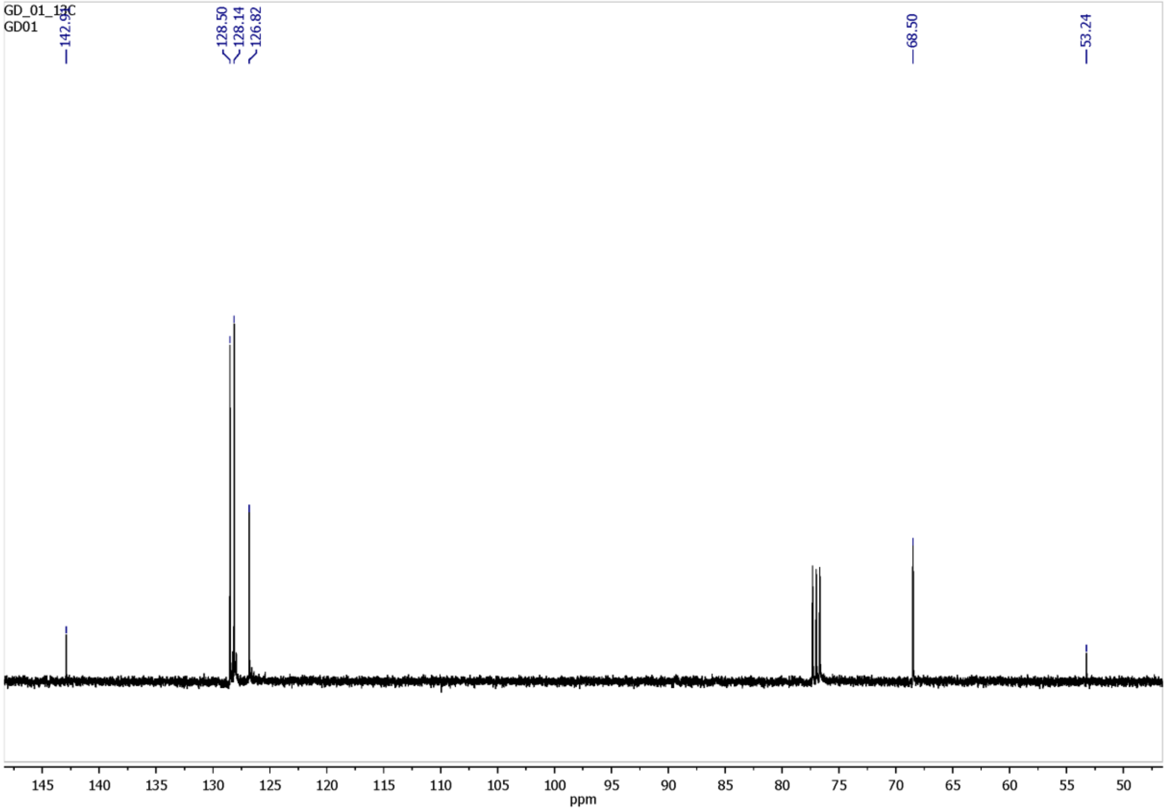

GD_01_13C
GD01
142.9
128.50
128.14
126.82
68.50
53.24
ppm

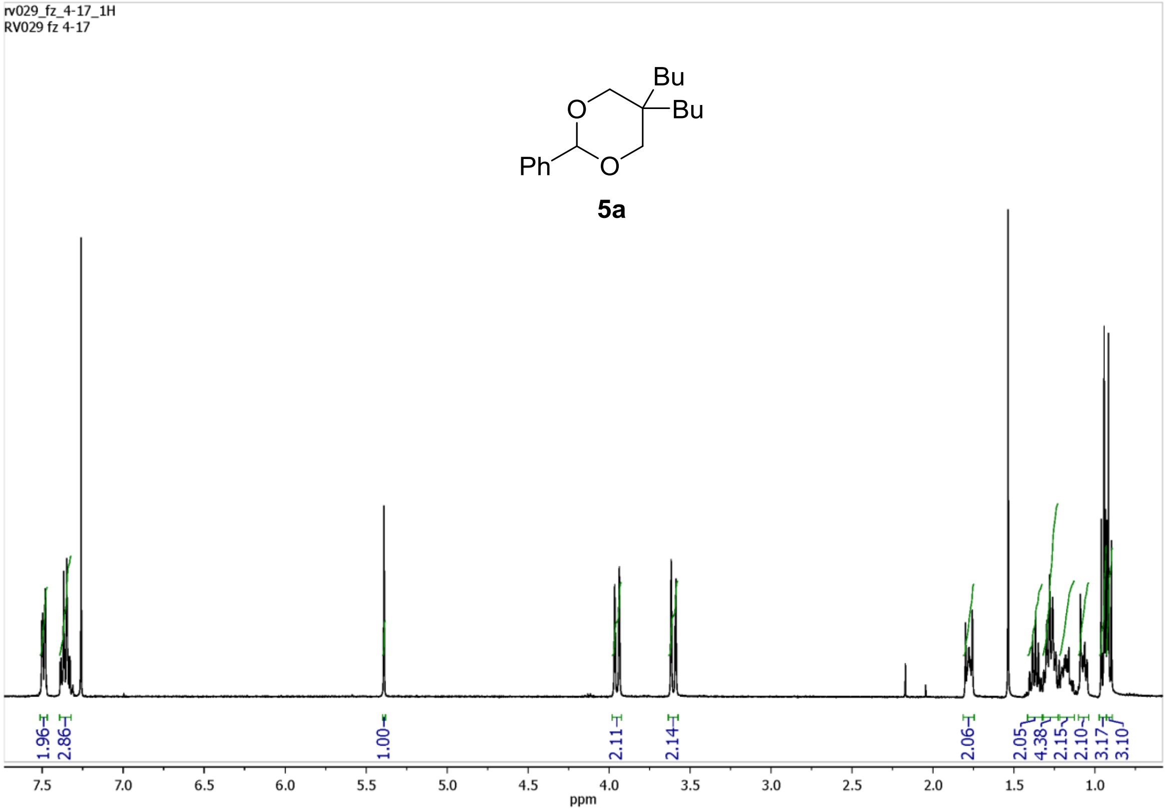
rv029_fz_4-17_1H
RV029 fz 4-17
Bu
Bu
O
O
Ph
5a
1.96
2.86
1.00
2.11
2.14
2.06
2.05
4.38
2.15
2.10
3.17
3.10
ppm

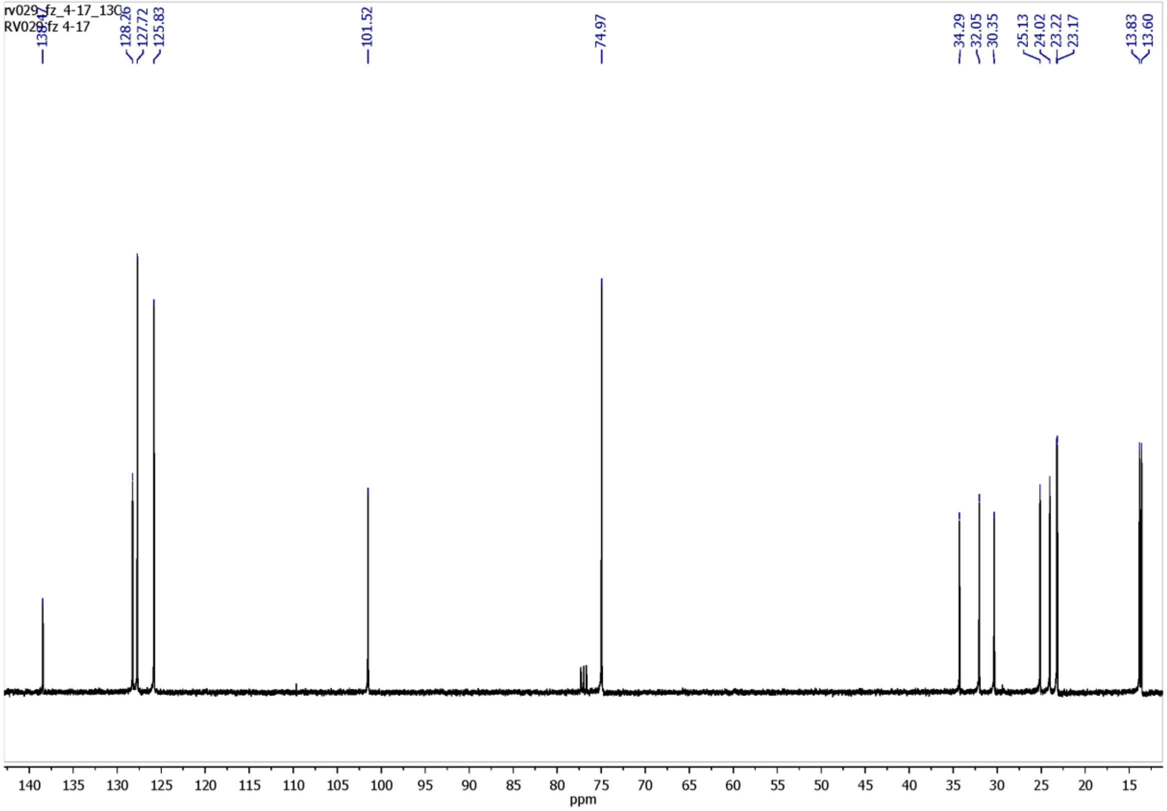
rv029_fz_4-17_13C
RV029 fz 4-17
138.47
128.26
127.72
125.83
101.52
74.97
34.29
32.05
30.35
25.13
24.02
23.22
23.17
13.83
13.60
ppm

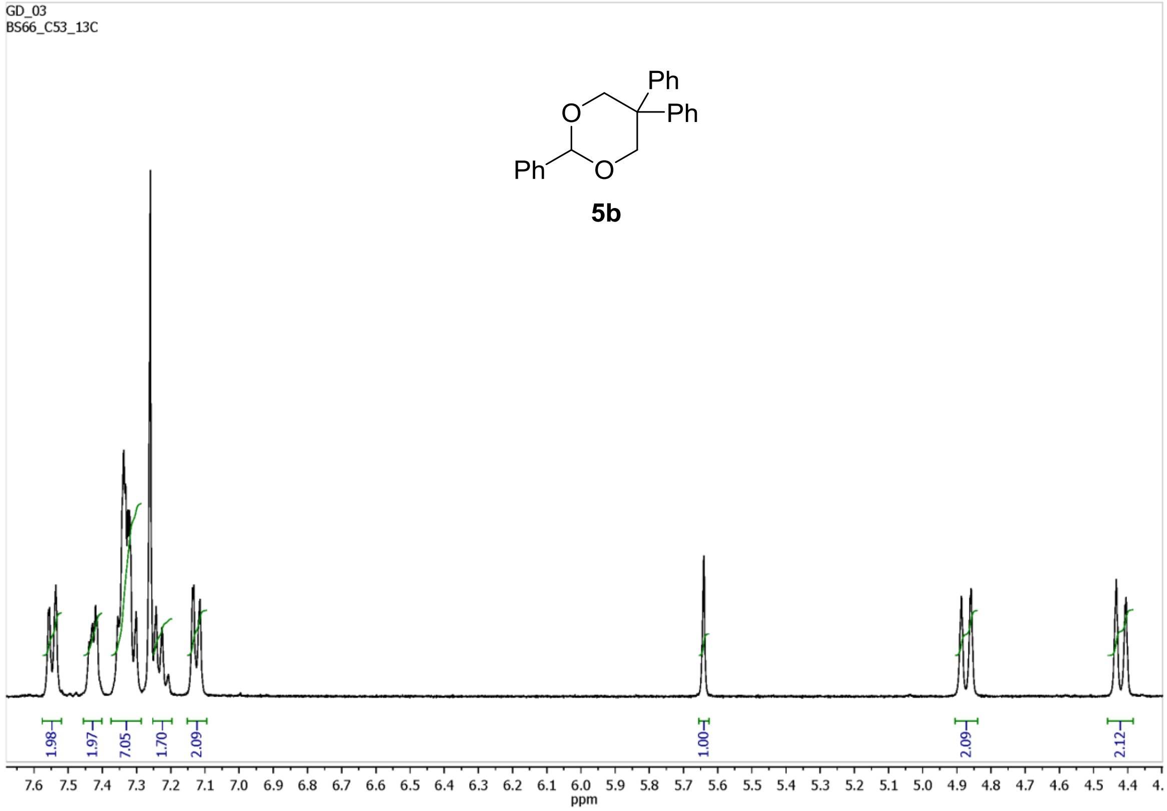
GD_03
BS66_C53_13C
Ph
O
Ph
Ph
O
5b
1.98
1.97
7.05
1.70
2.09
1.00
2.09
2.12
ppm

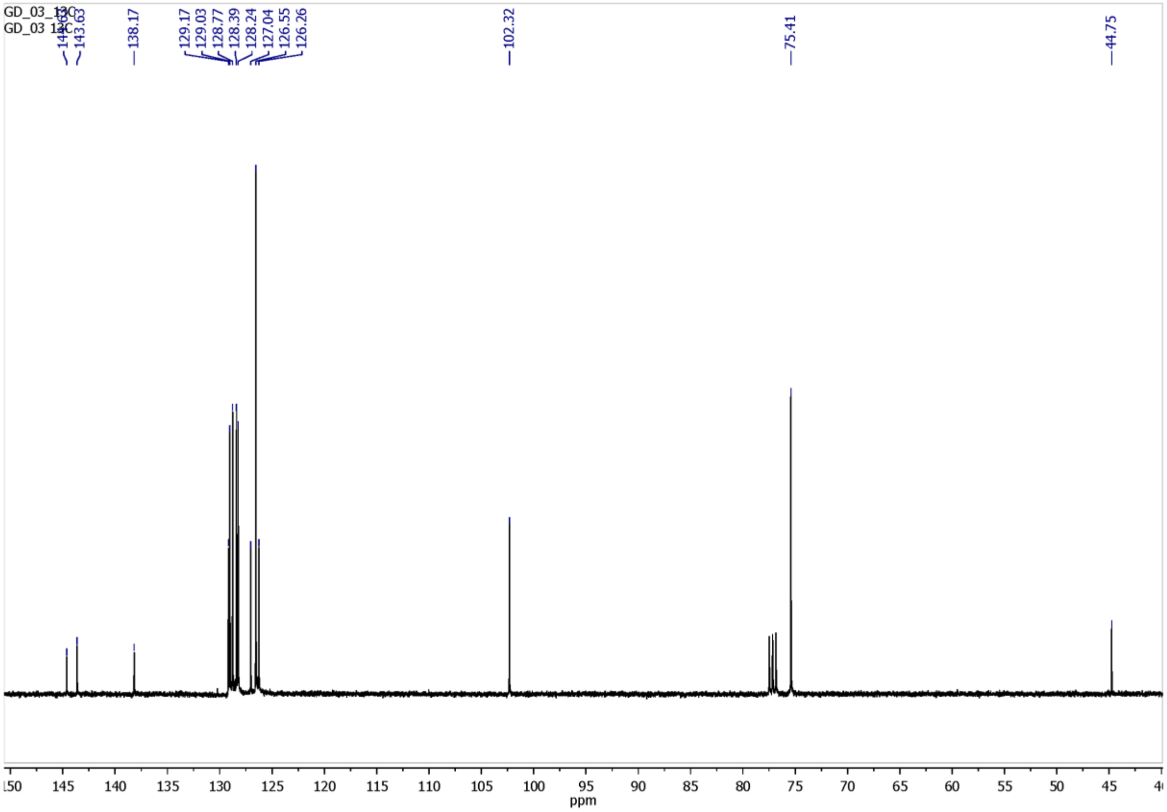
GD_03_13C
GD_03 13C
143.63
138.17
129.17
129.03
128.77
128.39
128.24
127.04
126.55
126.26
102.32
75.41
44.75
ppm

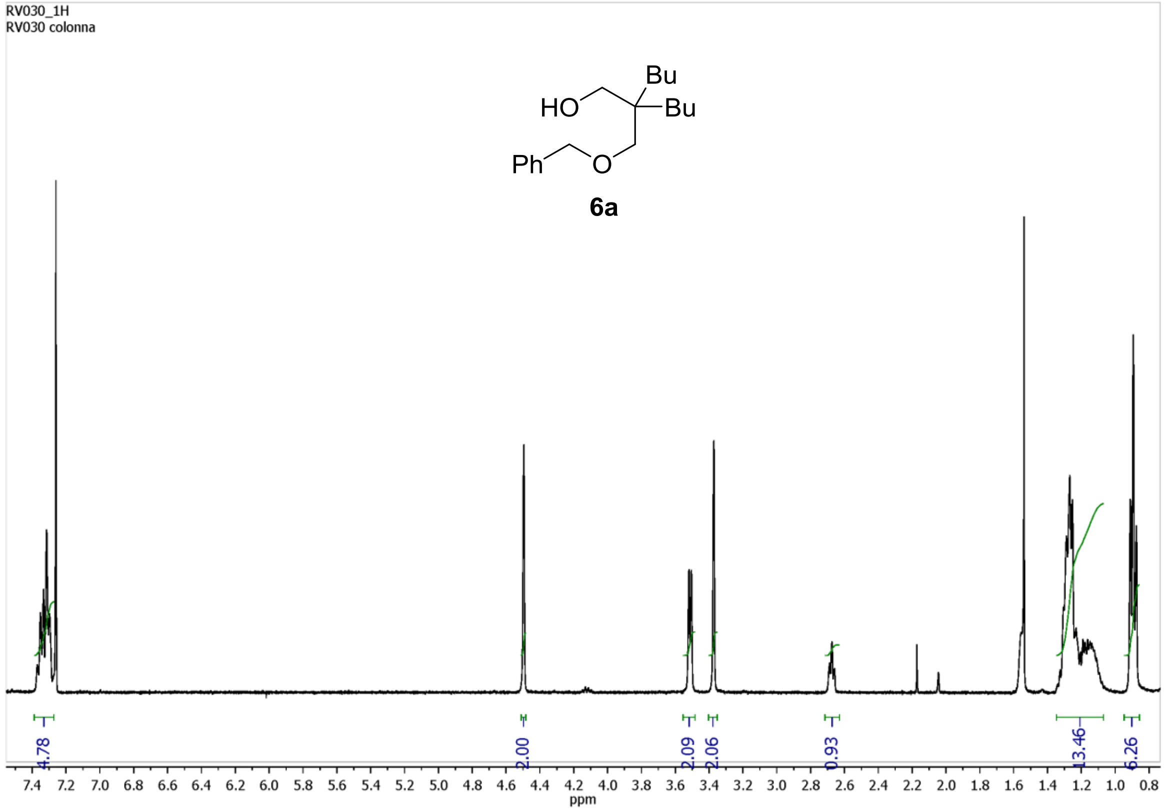
RV030_1H
RV030 colonna
HO
Bu
Bu
Ph
O
6a
4.78
2.00
2.09
2.06
0.93
13.46
6.26
ppm

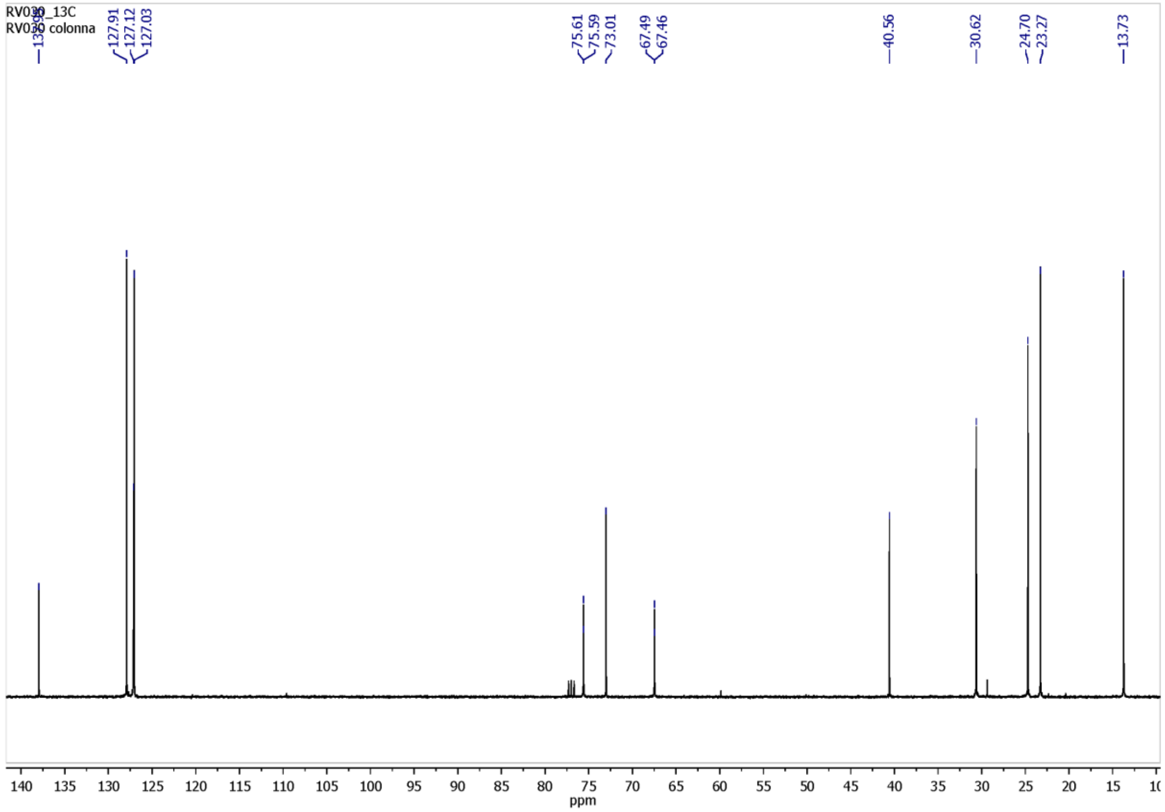
RV030_13C
RV030 colonna
127.91
127.12
127.03
75.61
75.59
73.01
67.49
67.46
40.56
30.62
24.70
23.27
13.73
ppm

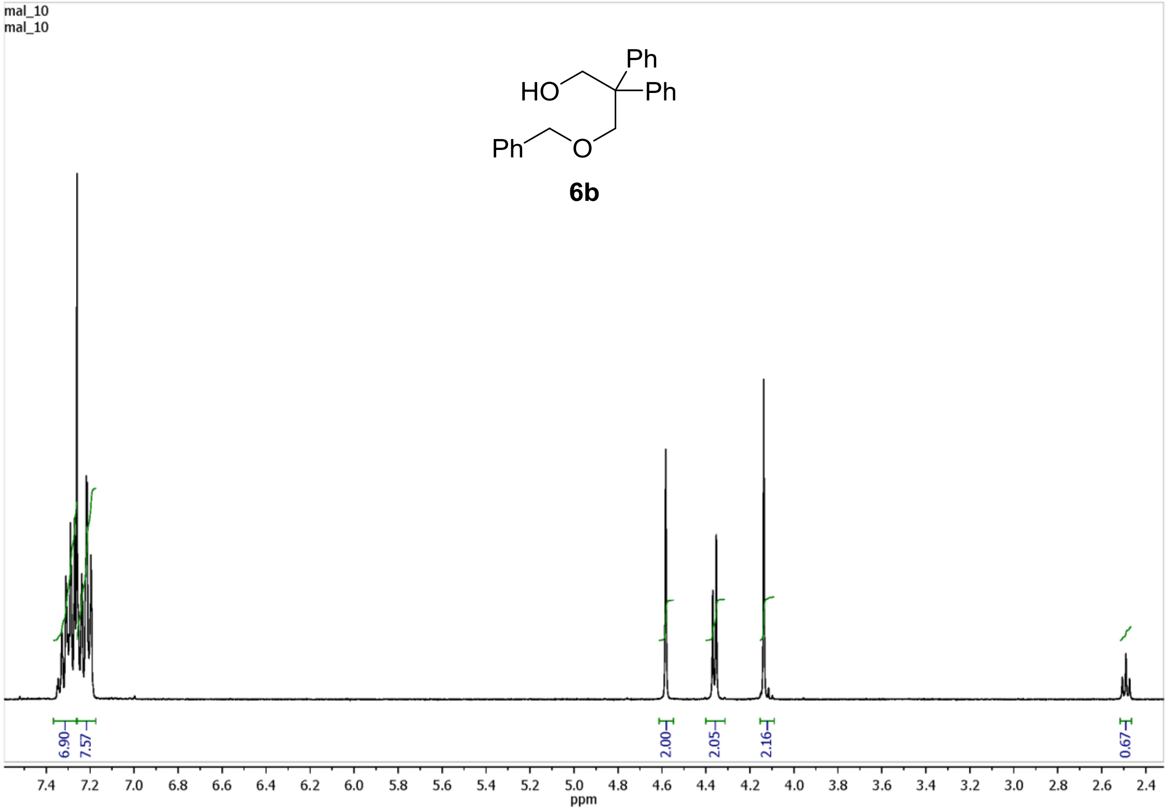
mal_10
mal_10
HO
Ph
Ph
Ph
O
6b
6.90
7.57
2.00
2.05
2.16
0.67
ppm

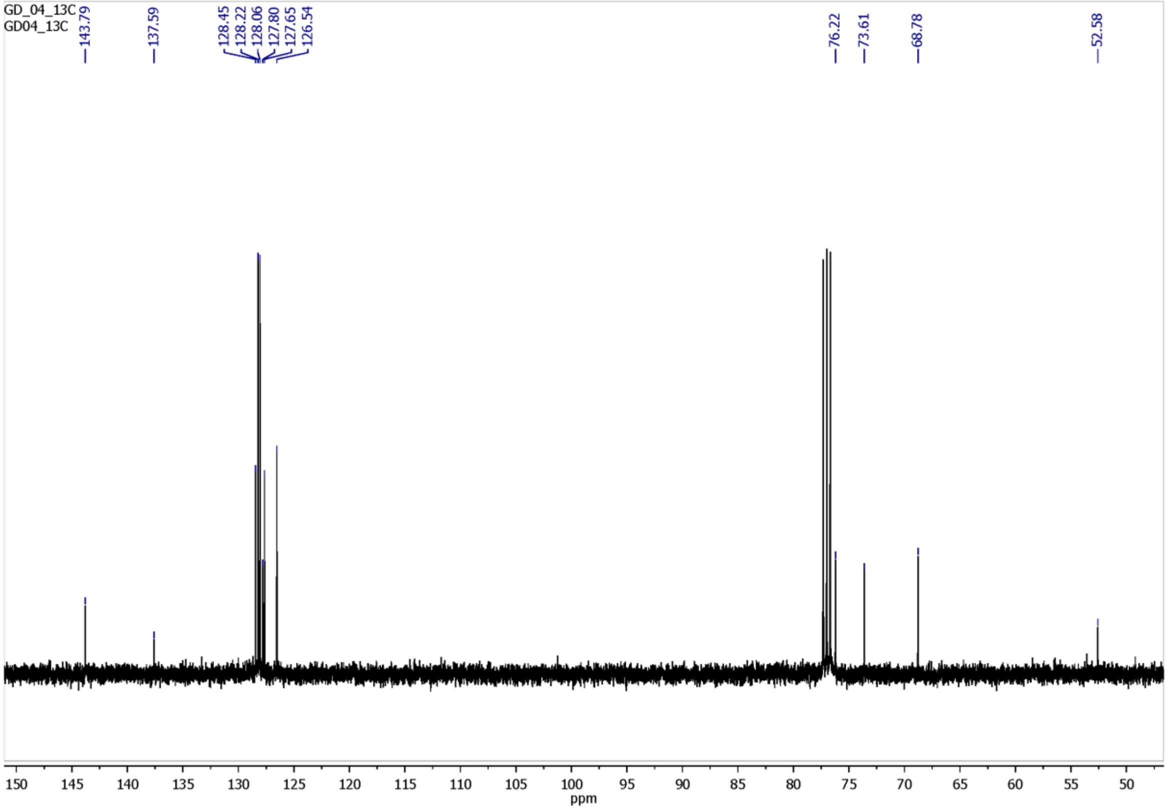
GD_04_13C
GD04_13C
143.79
137.59
128.45
128.22
128.06
127.80
127.65
126.54
76.22
73.61
68.78
52.58
ppm

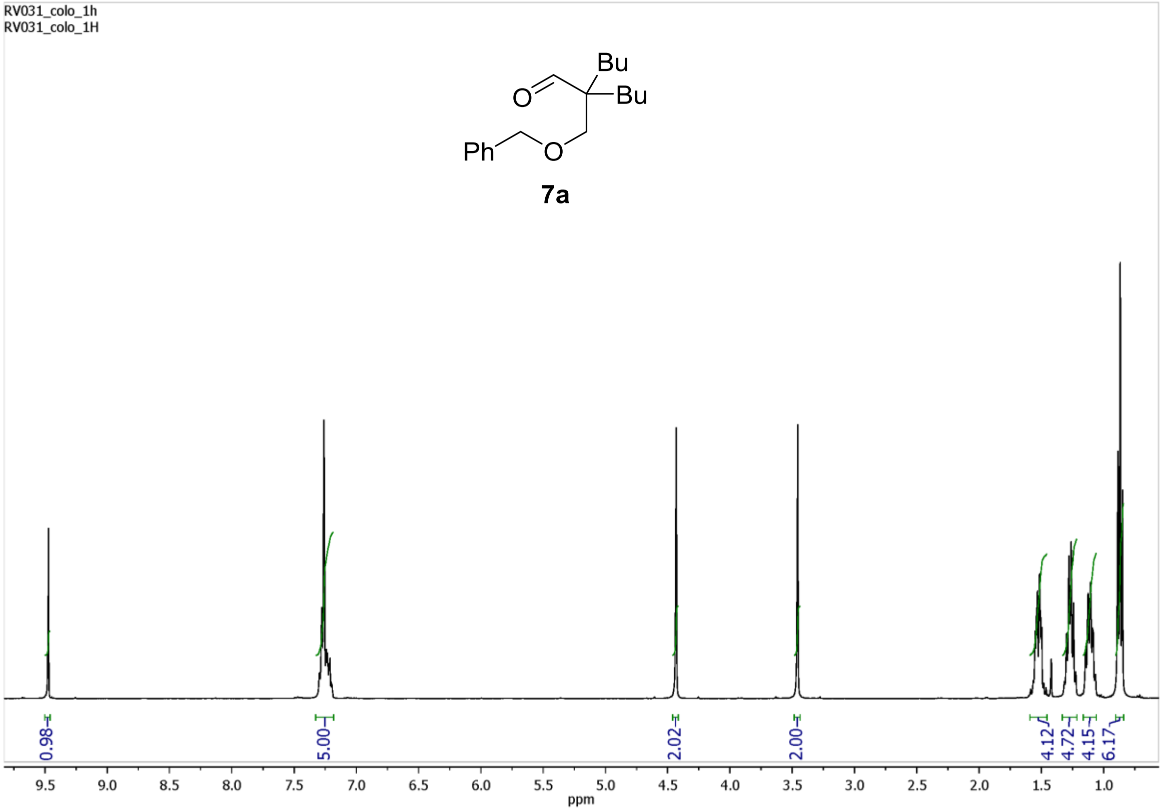
RV031_colo_1h
RV031_colo_1H
Bu
Bu
O
Ph
O
7a
0.98
5.00
2.02
2.00
4.12
4.72
4.15
6.17
ppm

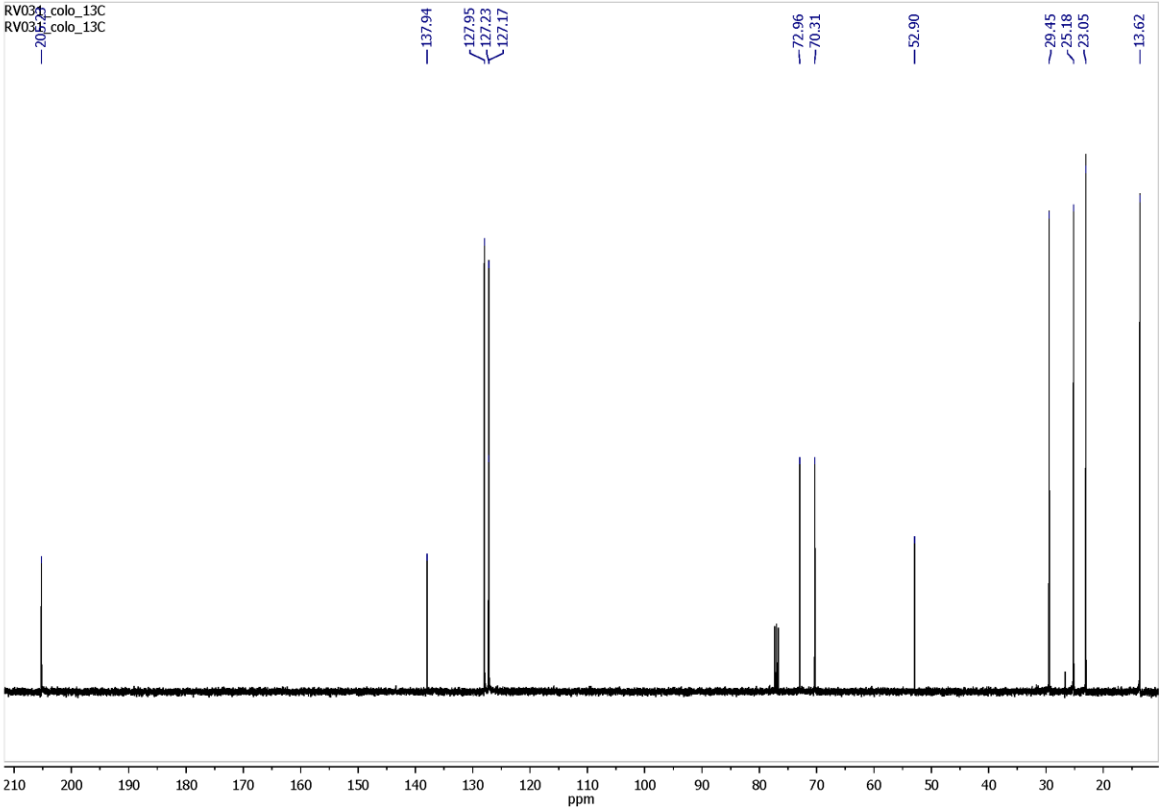
RV031_colo_13C
RV031_colo_13C
137.94
127.95
127.23
127.17
72.96
70.31
52.90
29.45
25.18
23.05
13.62
ppm

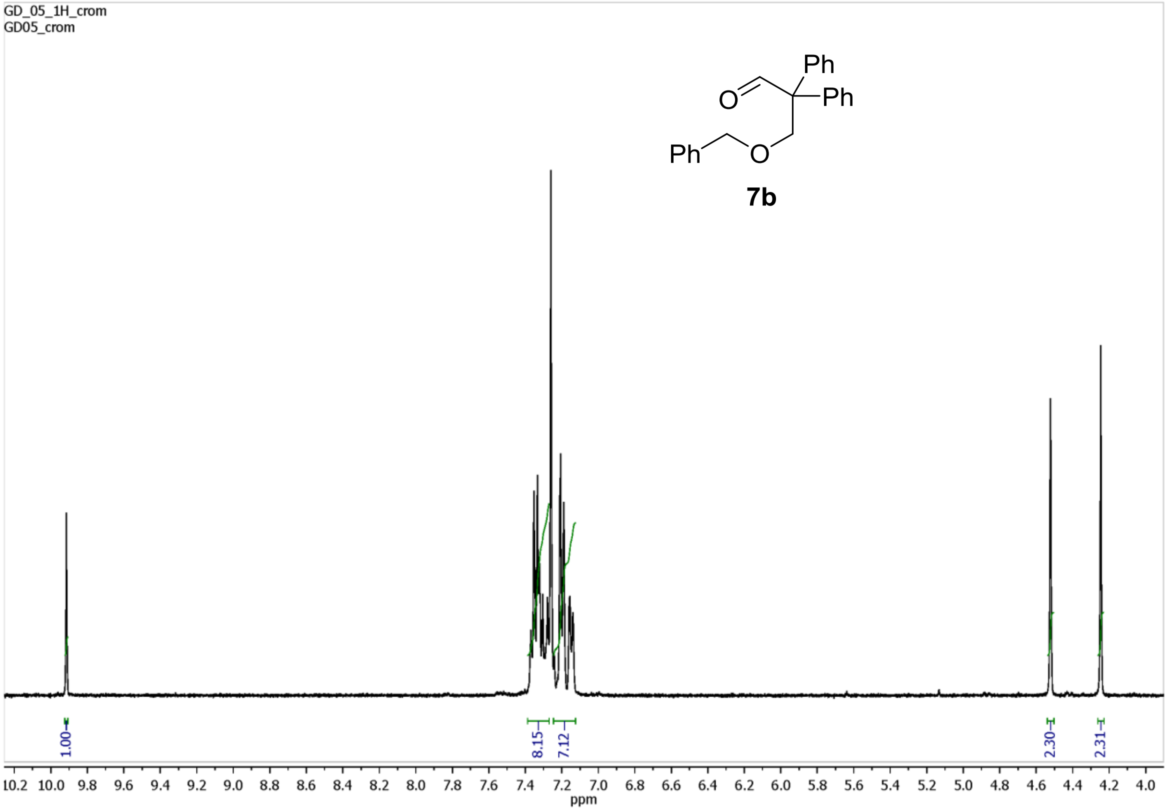
GD_05_1H_crom
GD05_crom
Ph
Ph
O
Ph
O
7b
1.00
8.15
7.12
2.30
2.31
ppm

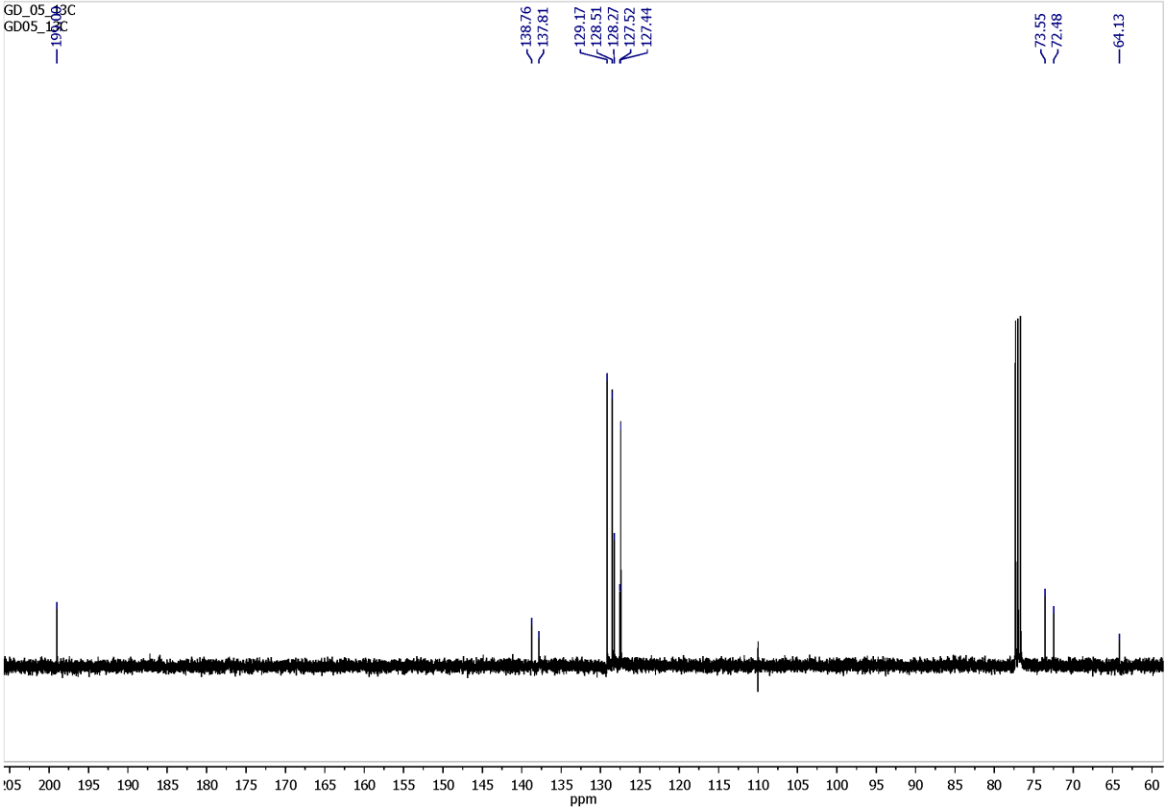
GD_05_13C
GD05_13C
138.76
137.81
129.17
128.51
128.27
127.52
127.44
73.55
72.48
64.13
ppm

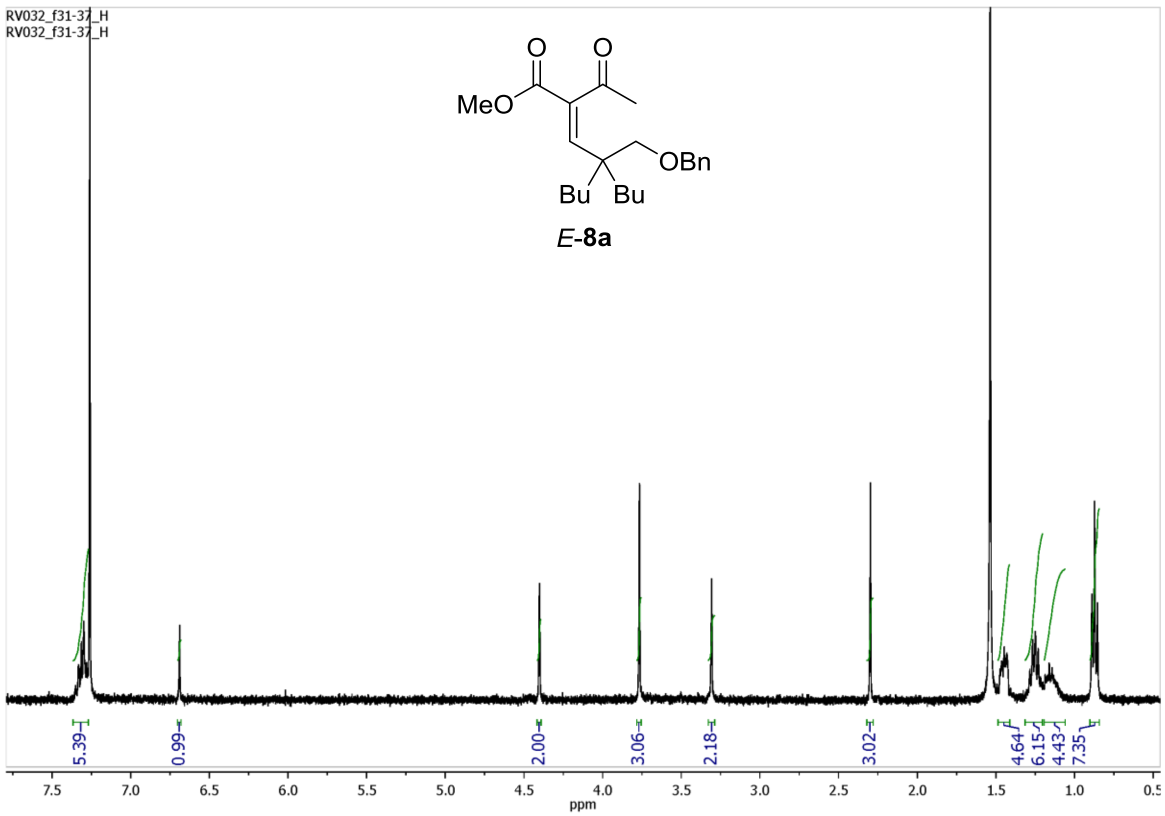
RV032_f31-37_H
RV032_f31-37_H
O
O
MeO
OBn
Bu
Bu
E-8a
5.39
0.99
2.00
3.06
2.18
3.02
4.64
6.15
4.43
7.35
7.5
7.0
6.5
6.0
5.5
5.0
4.5
4.0
3.5
3.0
2.5
2.0
1.5
1.0
ppm

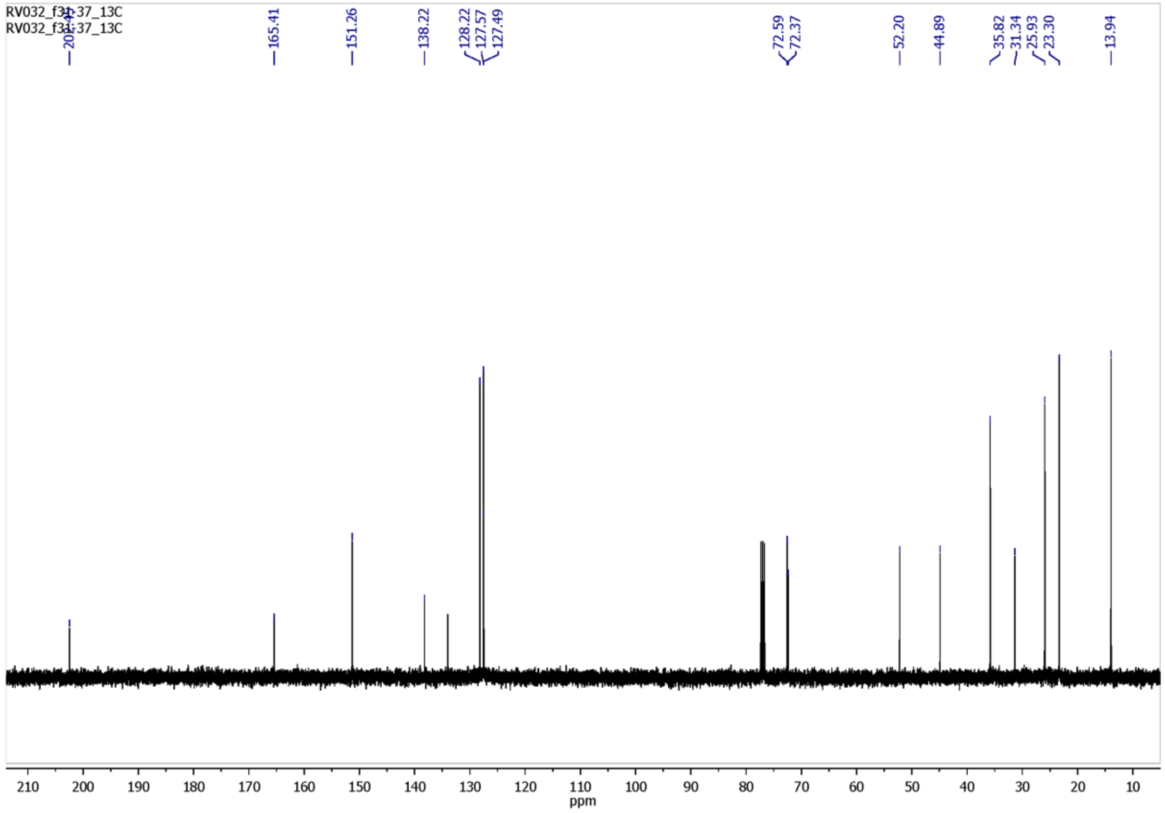
RV032_f31-37_13C
RV032_f31-37_13C
165.41
151.26
138.22
128.22
127.57
127.49
72.59
72.37
52.20
44.89
35.82
31.34
25.93
23.30
13.94
210
200
190
180
170
160
150
140
130
120
110
100
90
80
70
60
50
40
30
20
10
ppm

^{1}H NOEDIFF NMR analysis.

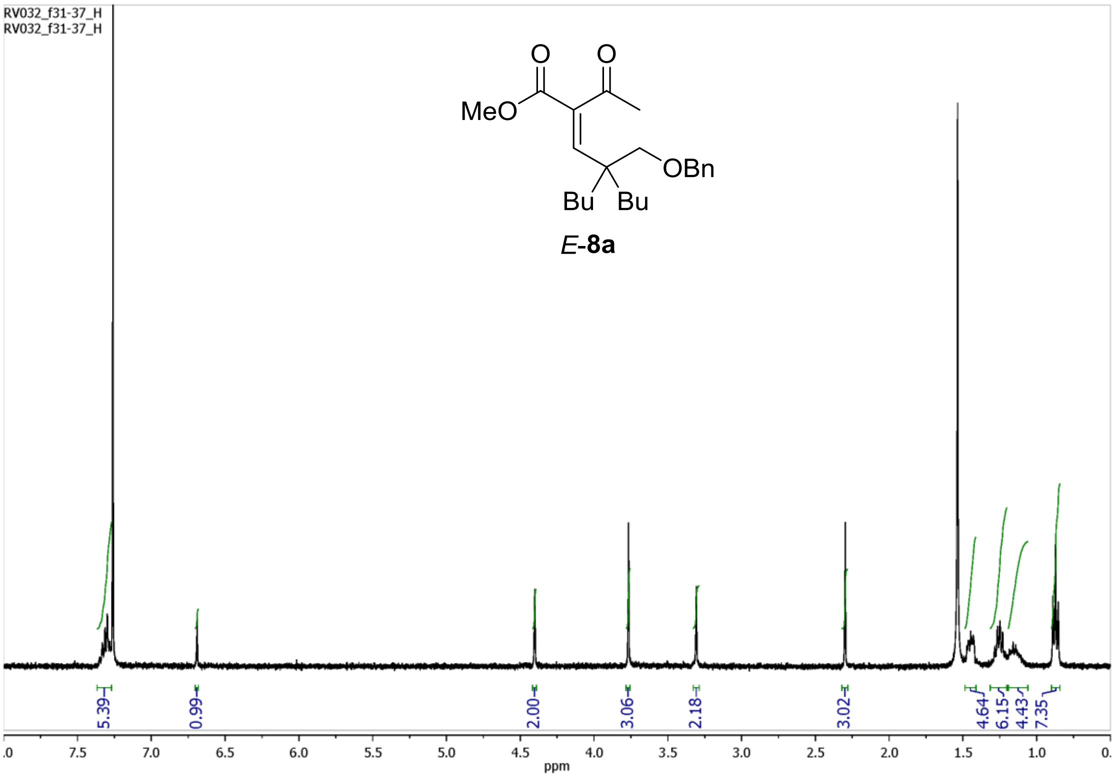

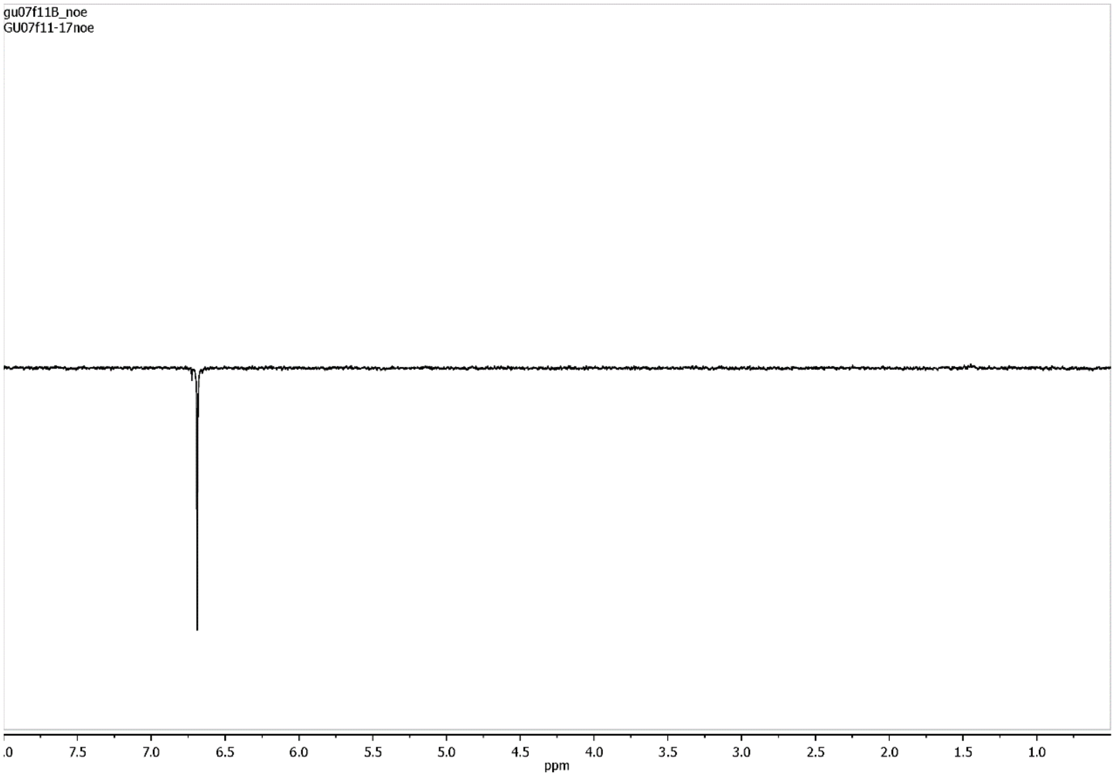

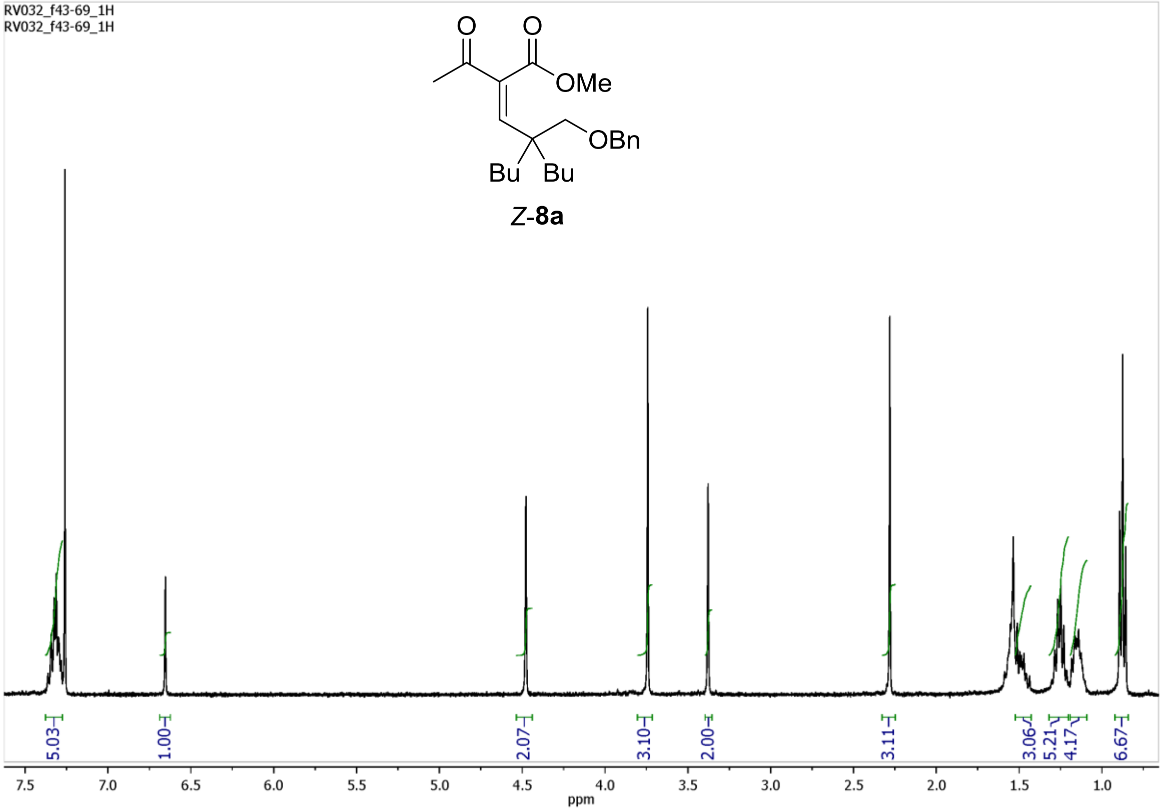
RV032_f43-69_1H
RV032_f43-69_1H
OMe
OBn
Bu
Bu
Z-8a
5.03
1.00
2.07
3.10
2.00
3.11
3.06
5.21
4.17
6.67
ppm

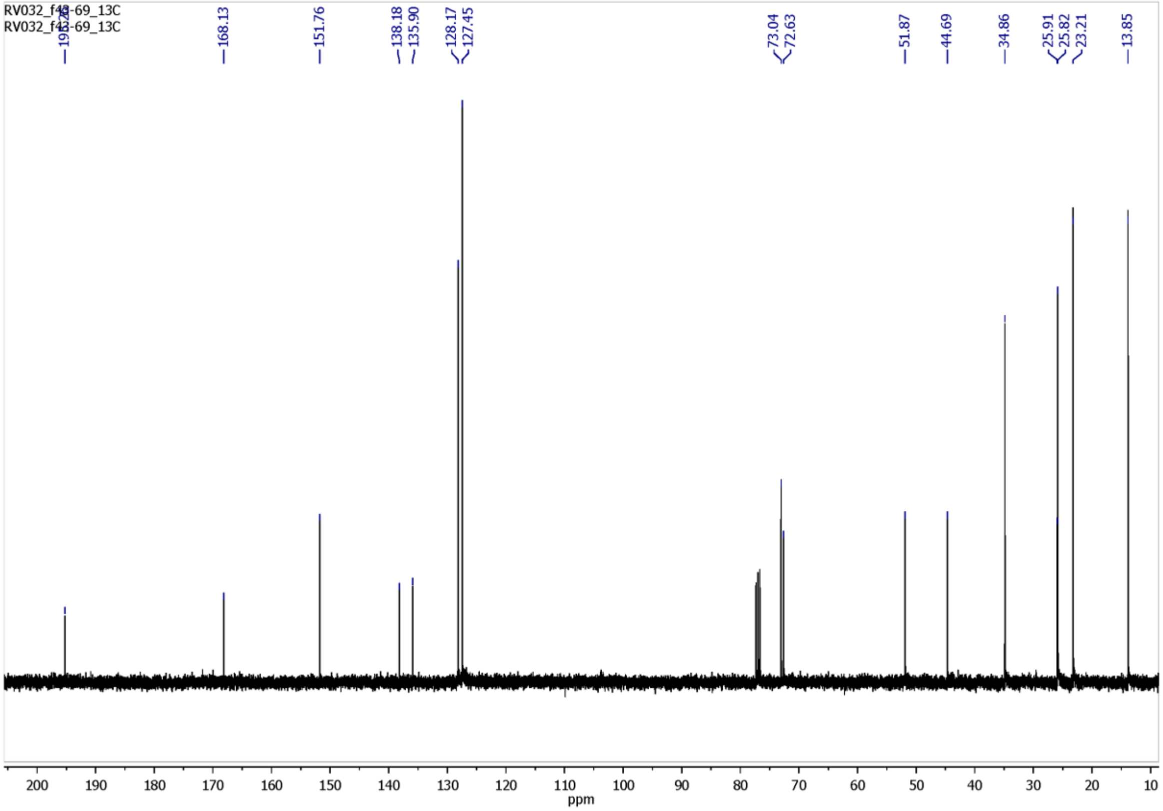
RV032_f43-69_13C
RV032_f43-69_13C
168.13
151.76
138.18
135.90
128.17
127.45
73.04
72.63
51.87
44.69
34.86
25.91
25.82
23.21
13.85
ppm

^{1}H NOEDIFF NMR analysis.

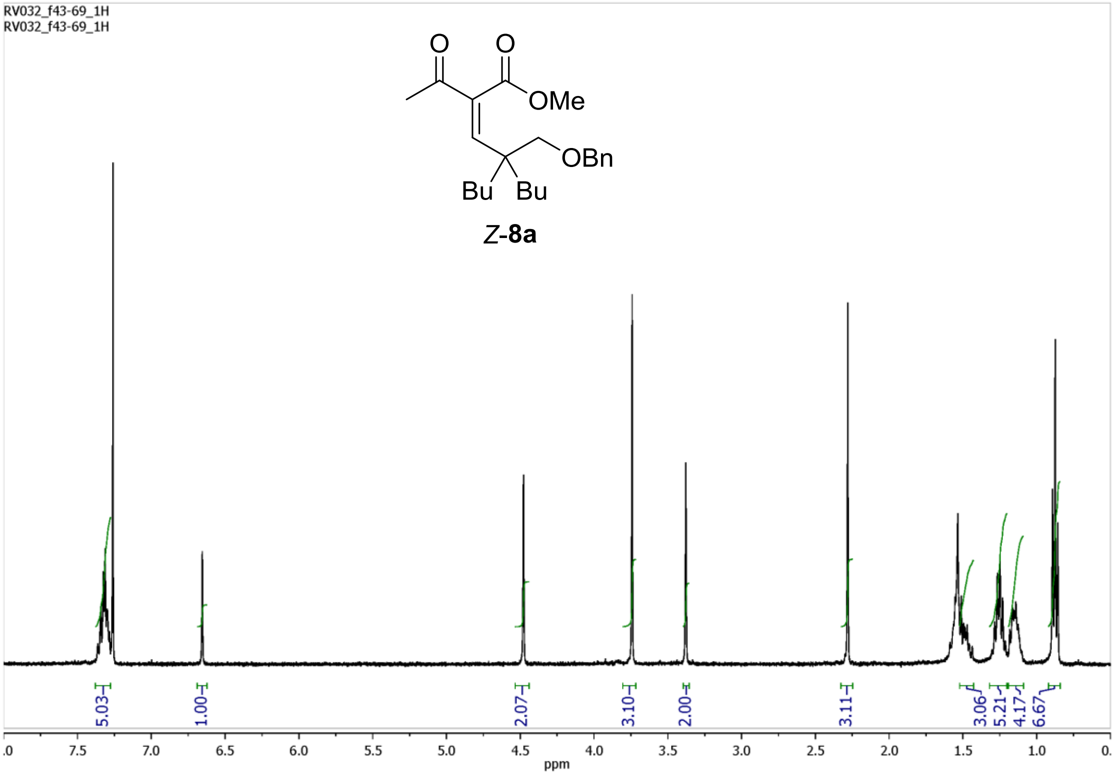

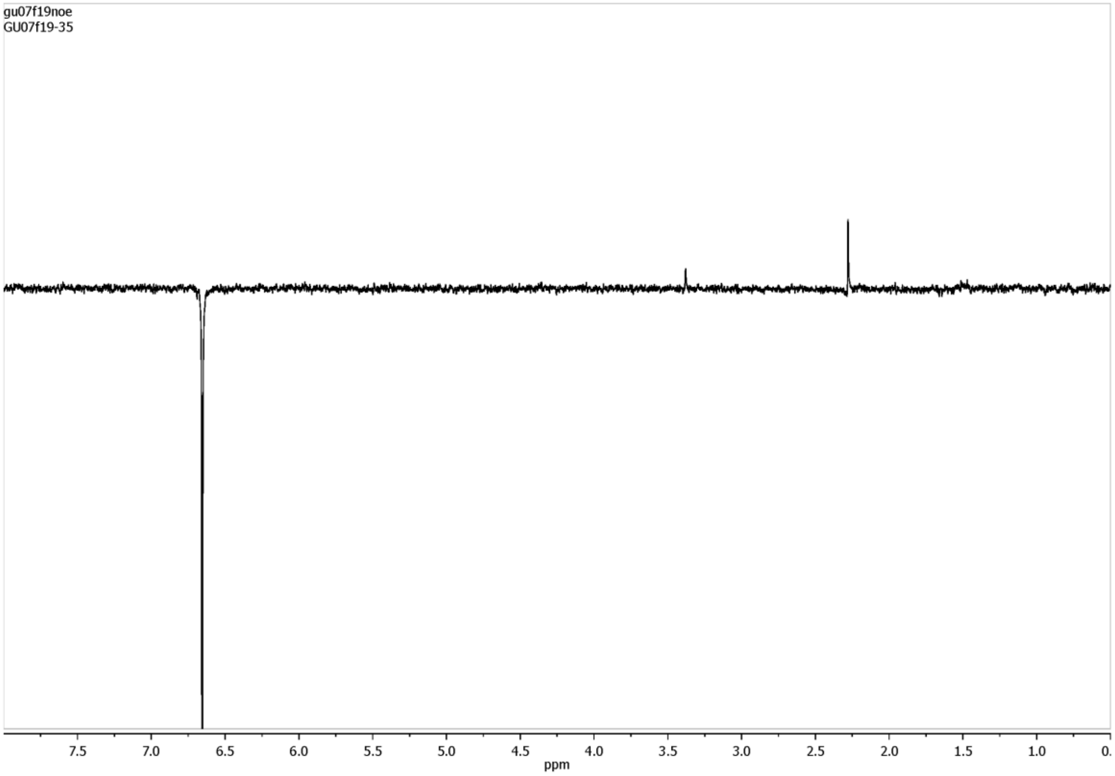

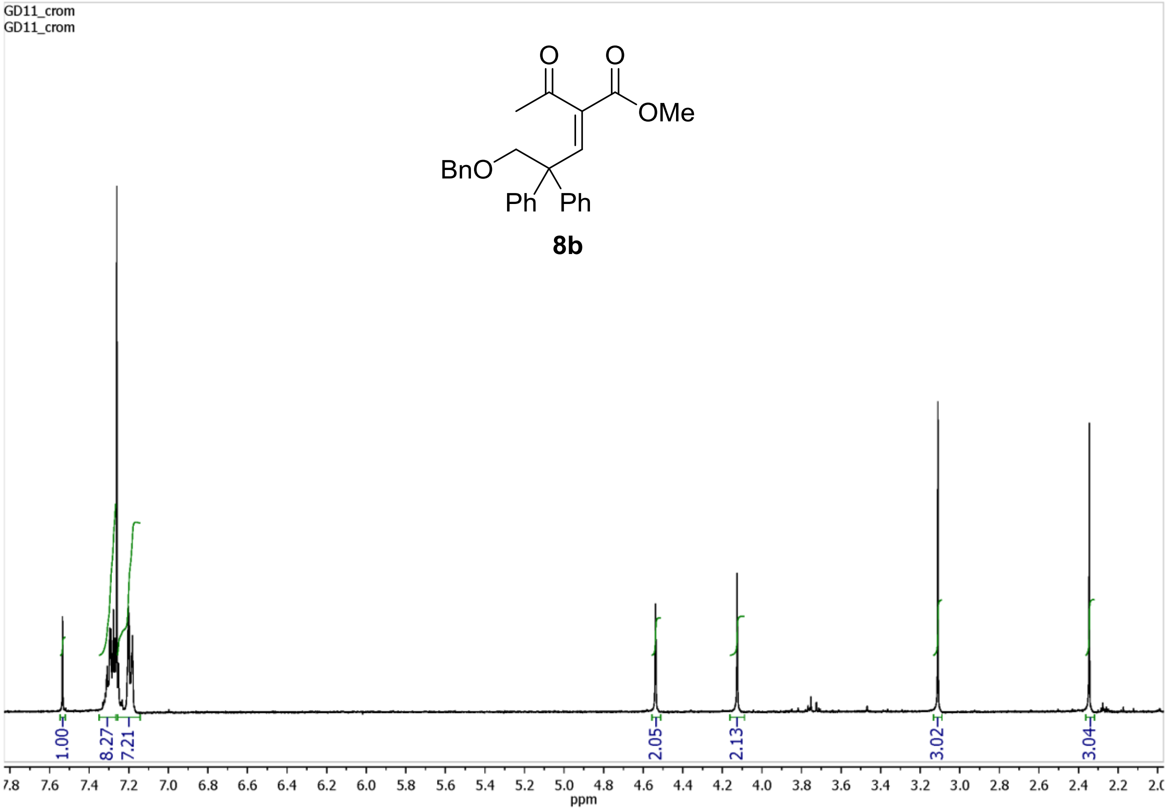
GD11_crom
GD11_crom
O
O
OMe
BnO
Ph
Ph
8b
1.00
8.27
7.21
2.05
2.13
3.02
3.04
ppm

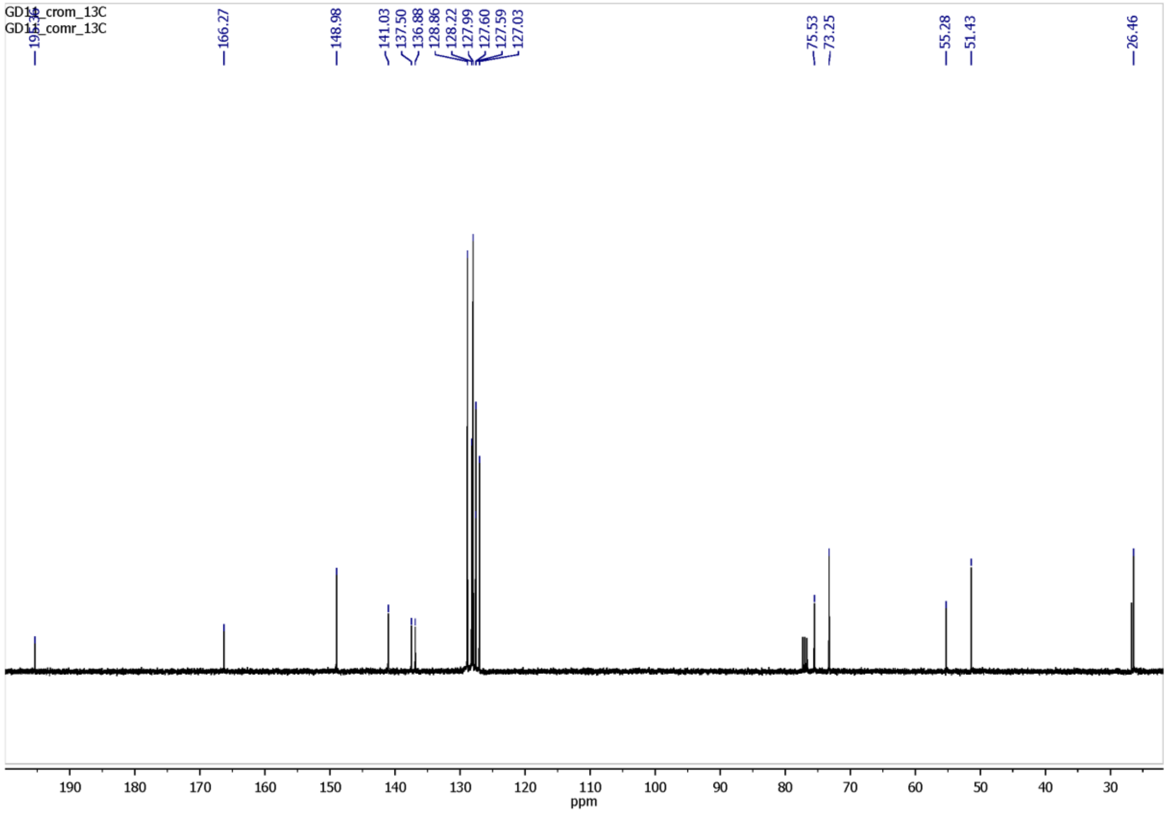
166.27
148.98
141.03
137.50
136.88
128.86
128.22
127.99
127.60
127.59
127.03
75.53
73.25
55.28
51.43
26.46
ppm

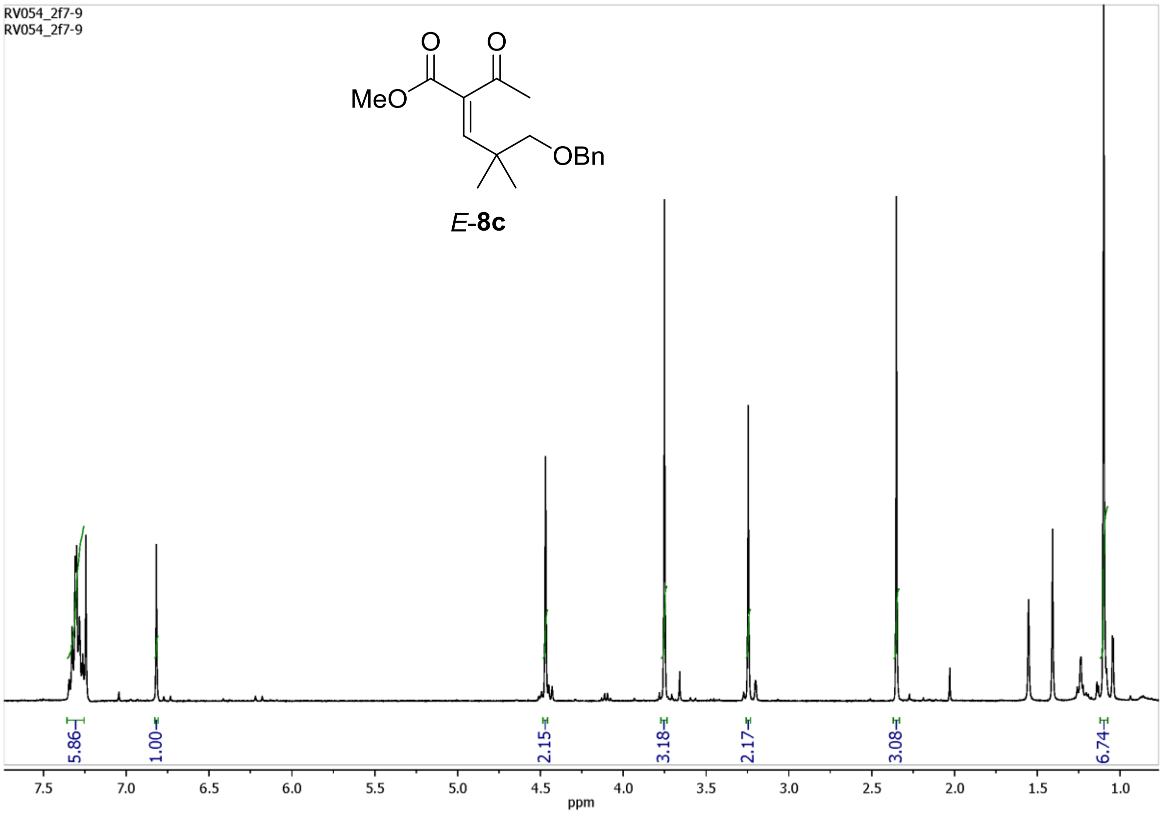
RV054_2f7-9
RV054_2f7-9
MeO
O
O
OBn
E-8c
5.86
1.00
2.15
3.18
2.17
3.08
6.74
7.5
7.0
6.5
6.0
5.5
5.0
4.5
4.0
3.5
3.0
2.5
2.0
1.5
1.0
ppm

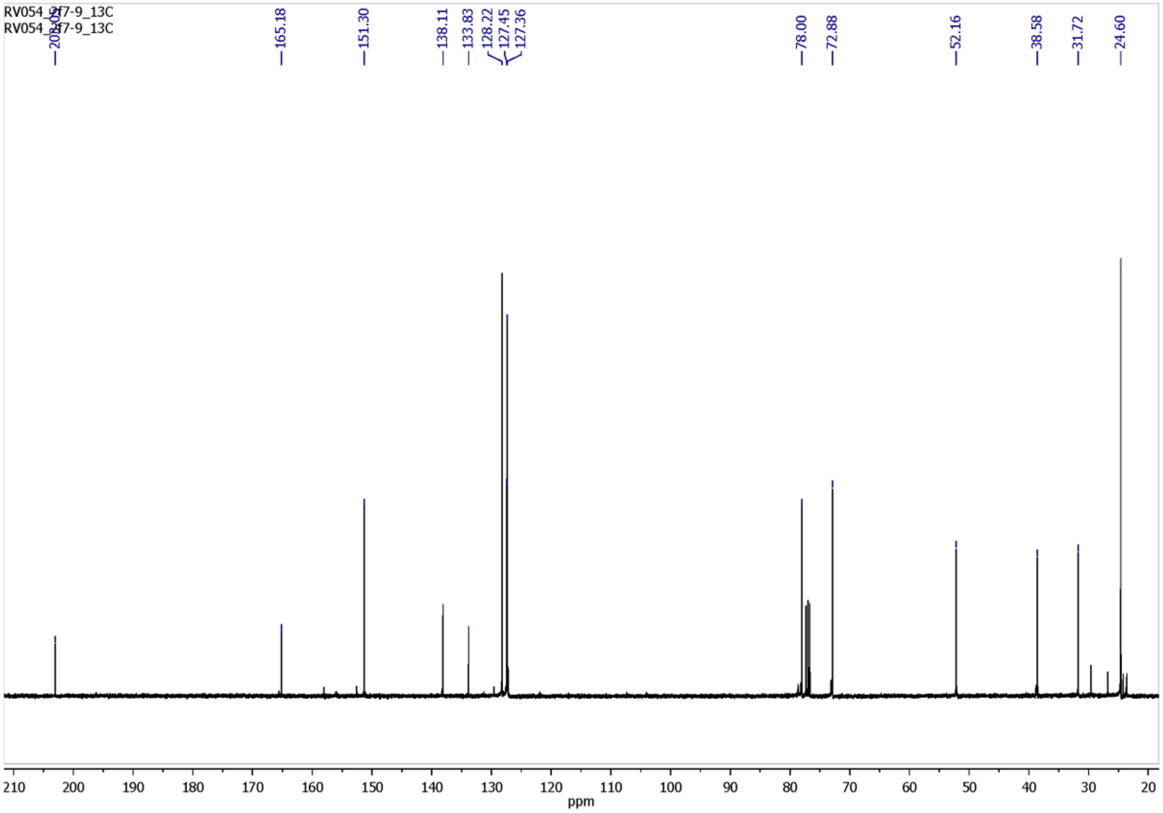
RV054_2f7-9_13C
RV054_2f7-9_13C
165.18
151.30
138.11
133.83
128.22
127.45
127.36
78.00
72.88
52.16
38.58
31.72
24.60
210
200
190
180
170
160
150
140
130
120
110
100
90
80
70
60
50
40
30
20
ppm

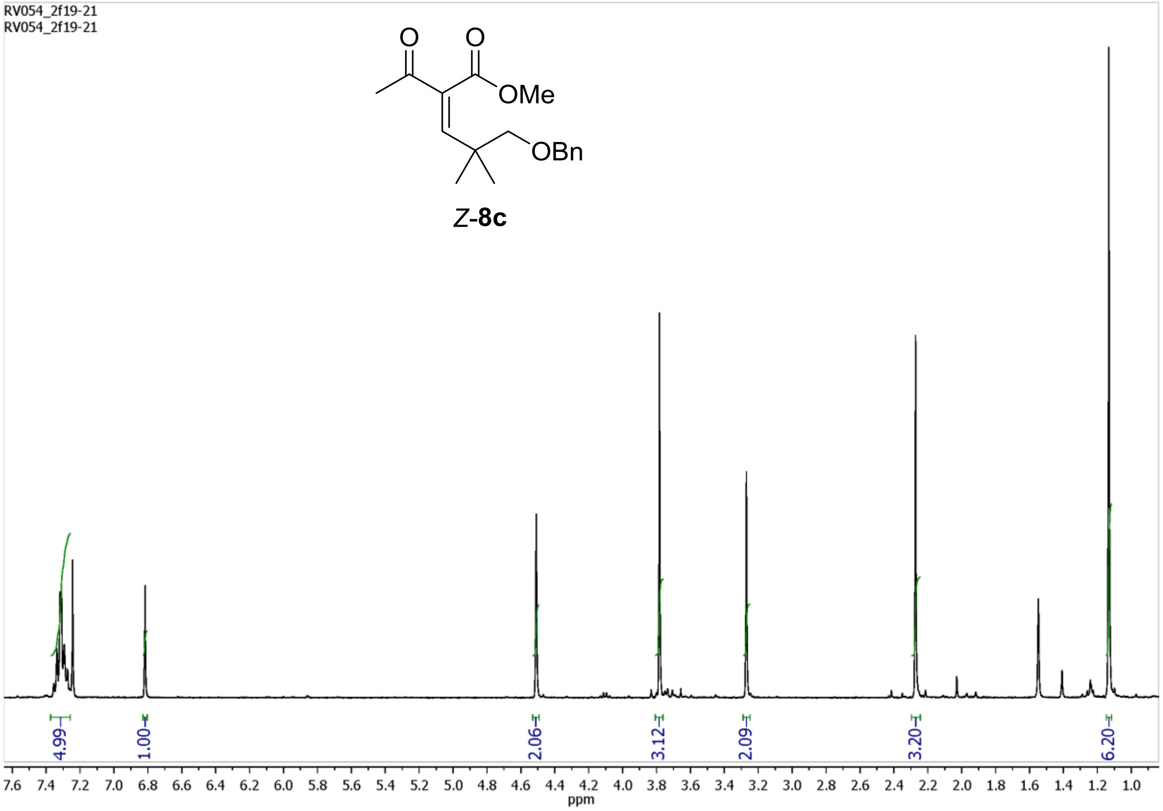

RV054_2f19-21
RV054_2f19-21
O
O
OMe
OBn
Z-8c
4.99
1.00
2.06
3.12
2.09
3.20
6.20
7.6 7.4 7.2 7.0 6.8 6.6 6.4 6.2 6.0 5.8 5.6 5.4 5.2 5.0 4.8 4.6 4.4 4.2 4.0 3.8 3.6 3.4 3.2 3.0 2.8 2.6 2.4 2.2 2.0 1.8 1.6 1.4 1.2 1.0
ppm

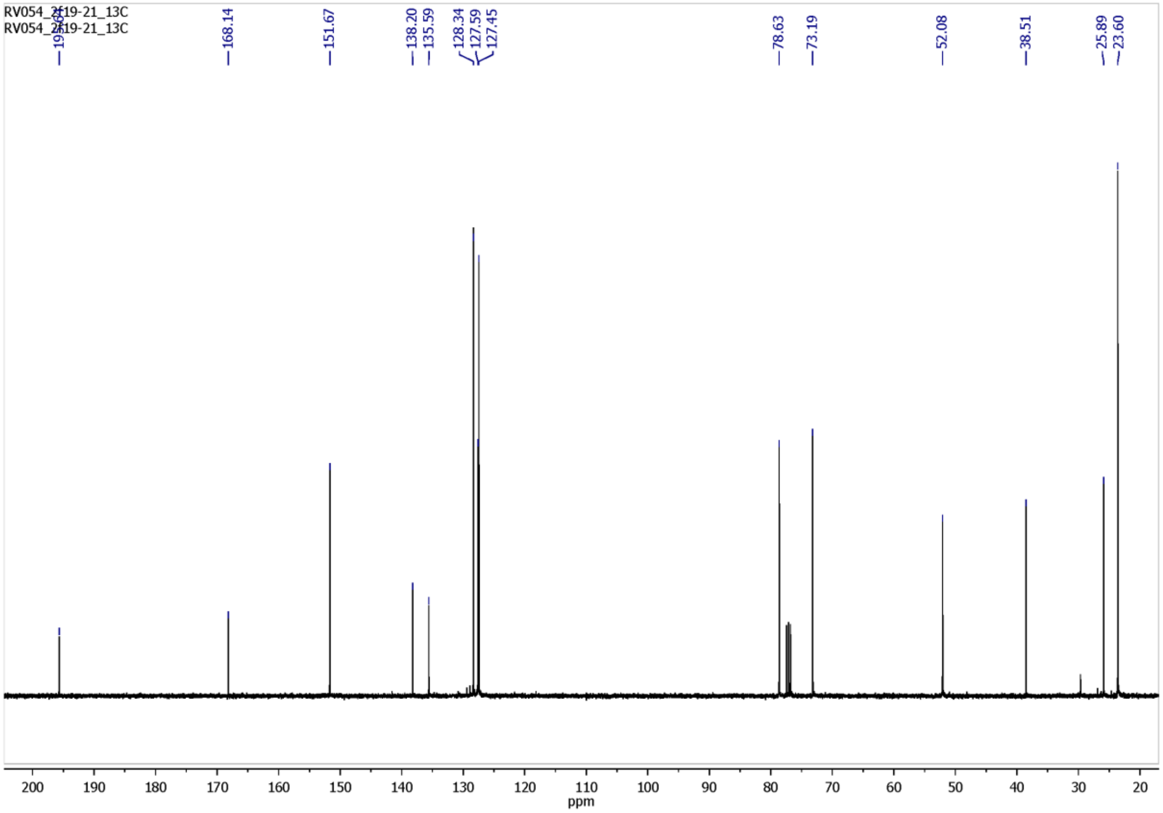

RV054_2f19-21_13C
RV054_2f19-21_13C
168.14
151.67
138.20
135.59
128.34
127.59
127.45
78.63
73.19
52.08
38.51
25.89
23.60
200 190 180 170 160 150 140 130 120 110 100 90 80 70 60 50 40 30 20
ppm

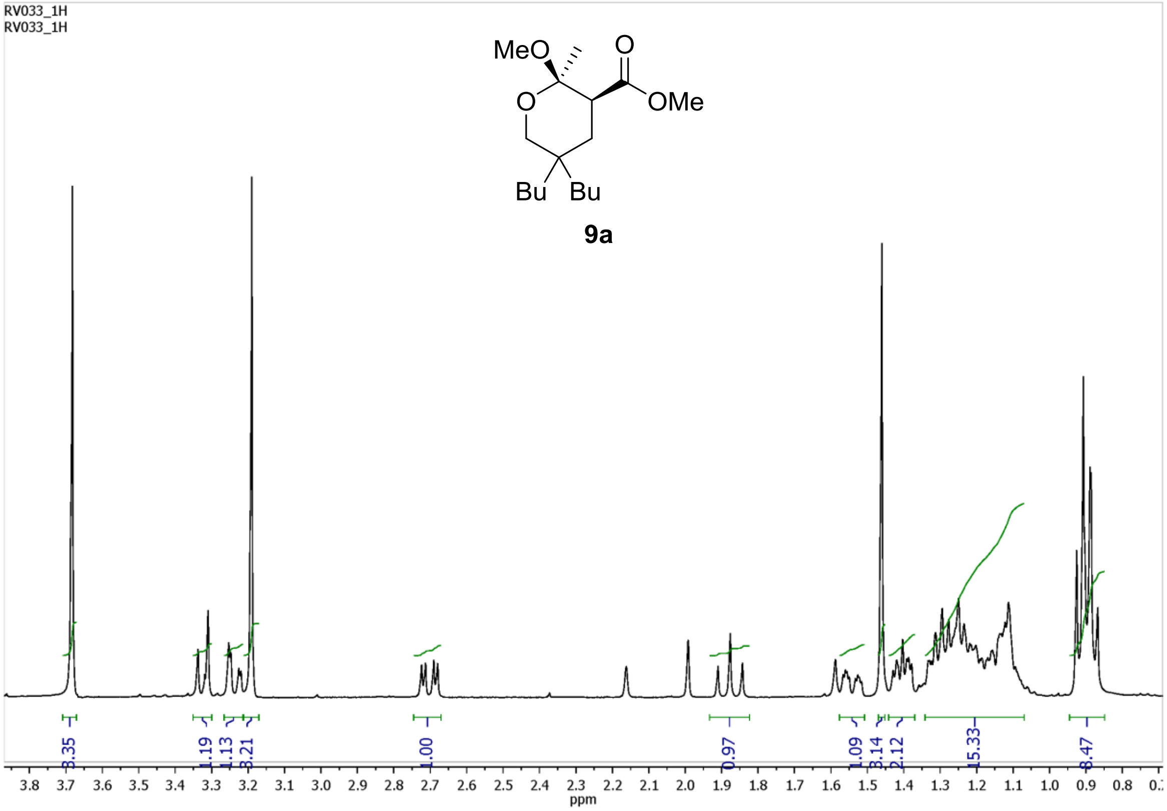
RV033_1H
RV033_1H
MeO
O
OMe
O
Bu
Bu
9a
3.35
1.19
1.13
3.21
1.00
0.97
1.09
3.14
2.12
15.33
8.47
ppm

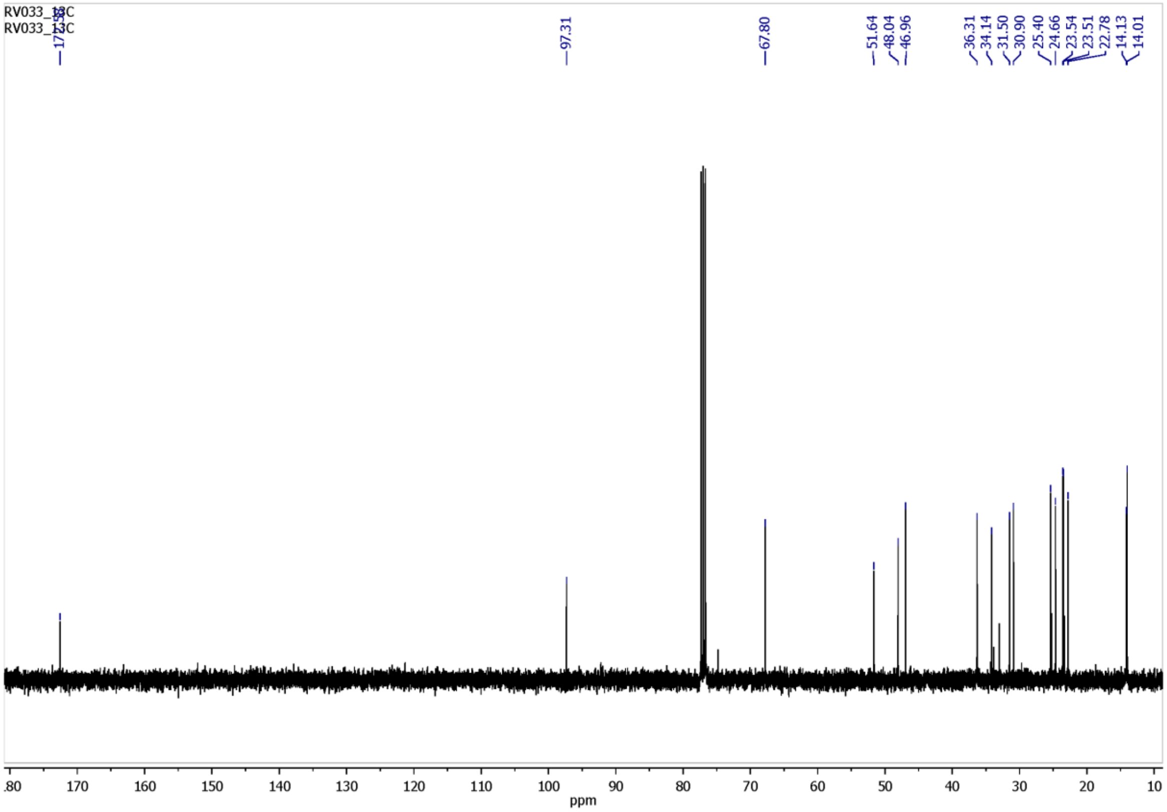
RV033_13C
RV033_13C
97.31
67.80
51.64
48.04
46.96
36.31
34.14
31.50
30.90
25.40
24.66
23.54
23.51
22.78
14.13
14.01
ppm

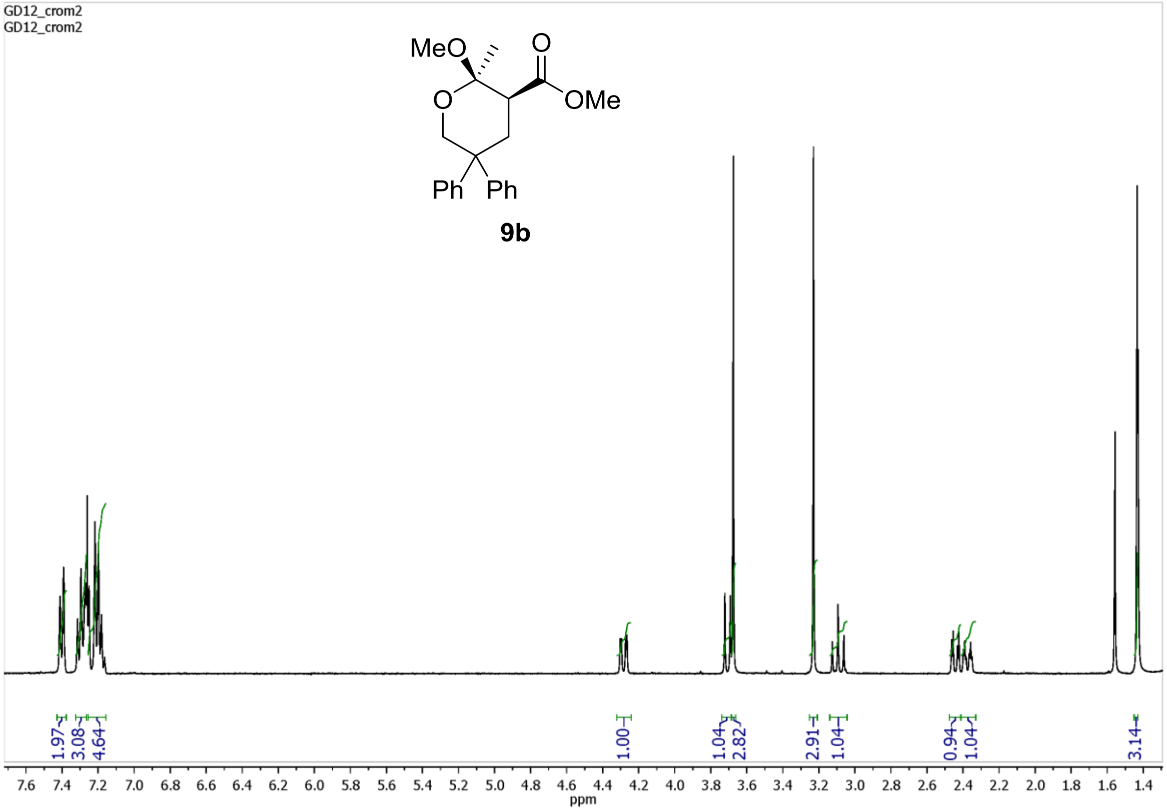
GD12_crom2
GD12_crom2
MeO
O
OMe
O
Ph
Ph
9b
1.97
3.08
4.64
1.00
1.04
2.82
2.91
1.04
0.94
1.04
3.14
7.6 7.4 7.2 7.0 6.8 6.6 6.4 6.2 6.0 5.8 5.6 5.4 5.2 5.0 4.8 4.6 4.4 4.2 4.0 3.8 3.6 3.4 3.2 3.0 2.8 2.6 2.4 2.2 2.0 1.8 1.6 1.4
ppm

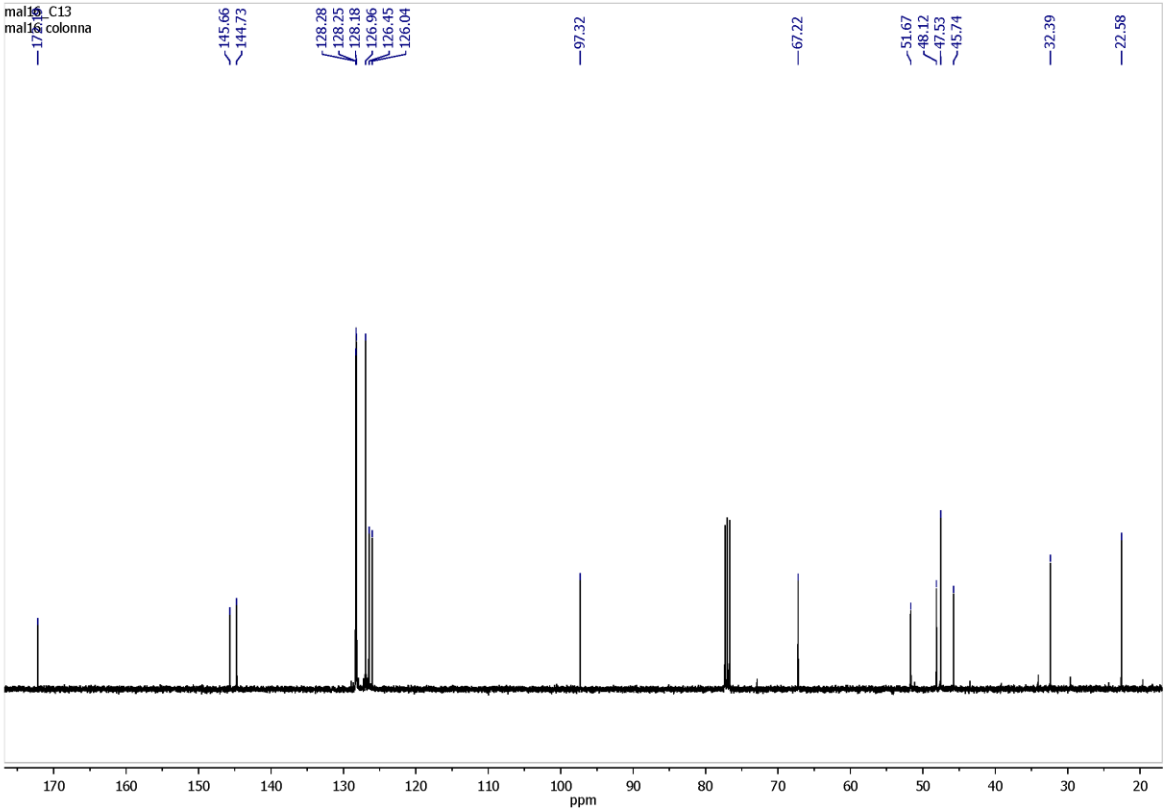
mal16_C13
mal16 colonna
145.66
144.73
128.28
128.25
128.18
126.96
126.45
126.04
97.32
67.22
51.67
48.12
47.53
45.74
32.39
22.58
170 160 150 140 130 120 110 100 90 80 70 60 50 40 30 20
ppm

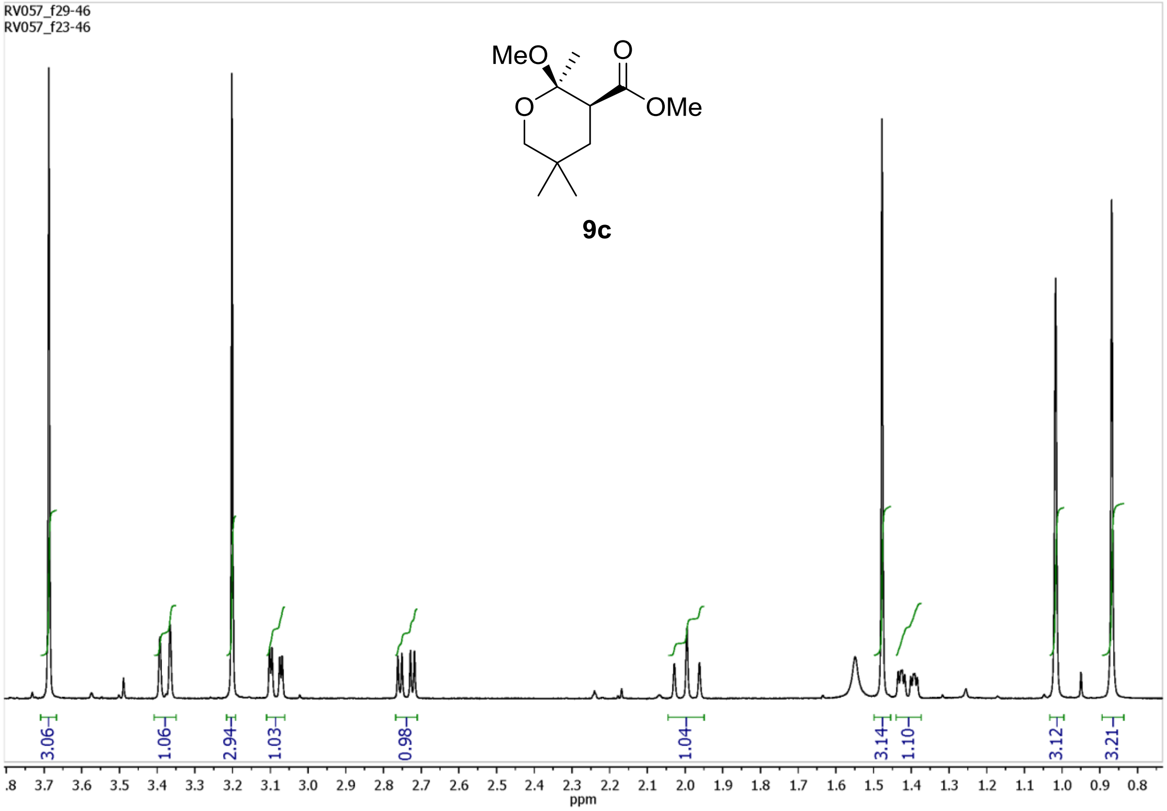
RV057_f29-46
RV057_f23-46
MeO
O
O
OMe
9c
3.06
1.06
2.94
1.03
0.98
1.04
3.14
1.10
3.12
3.21
ppm

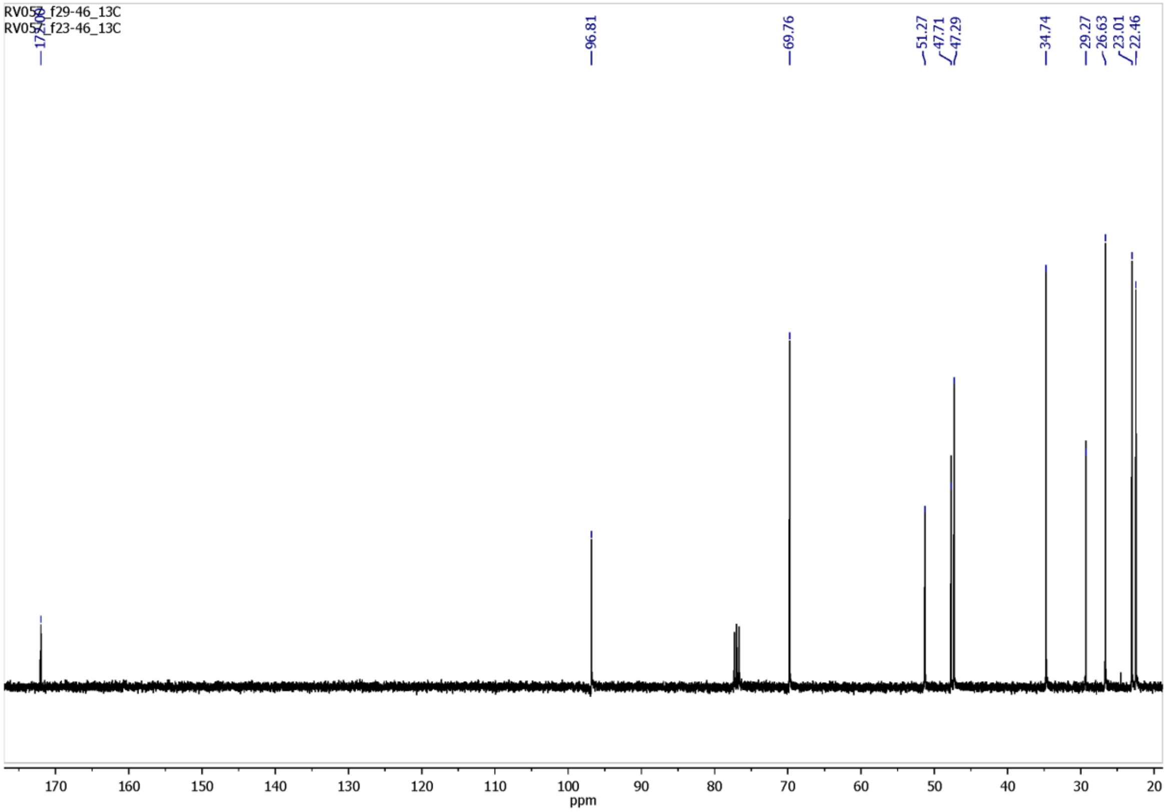
RV057_f29-46_13C
RV057_f23-46_13C
96.81
69.76
51.27
47.71
47.29
34.74
29.27
26.63
23.01
22.46
ppm

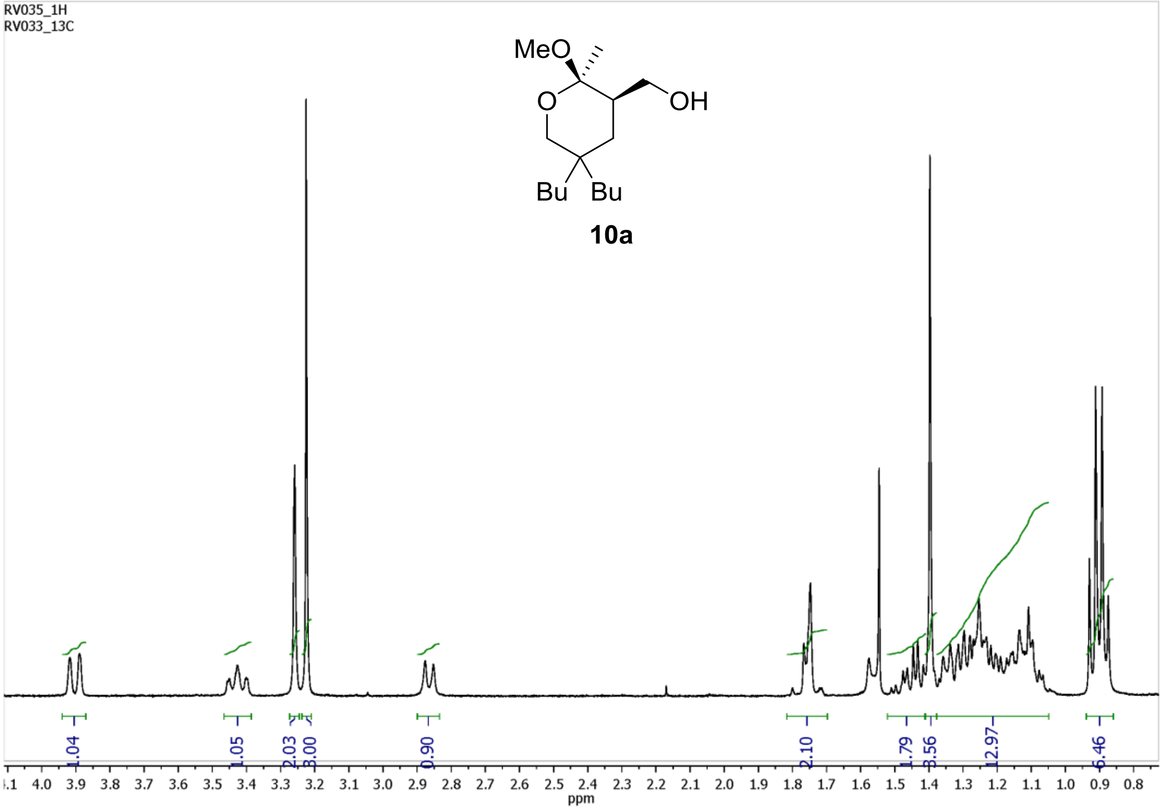
RV035_1H
RV033_13C
MeO
OH
O
Bu
Bu
10a
1.04
1.05
2.03
3.00
0.90
2.10
1.79
3.56
12.97
6.46
ppm

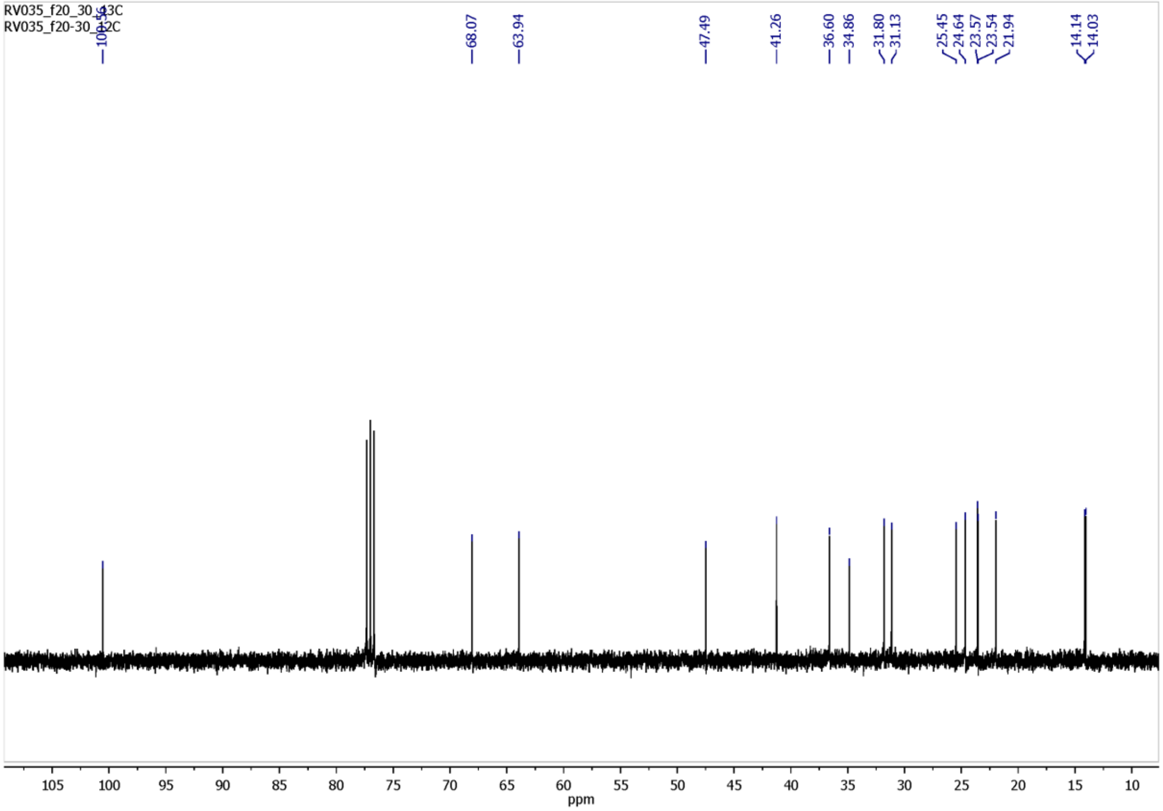
RV035_f20_30_13C
RV035_f20-30_12C
100.56
68.07
63.94
47.49
41.26
36.60
34.86
31.80
31.13
25.45
24.64
23.57
23.54
21.94
14.14
14.03
ppm

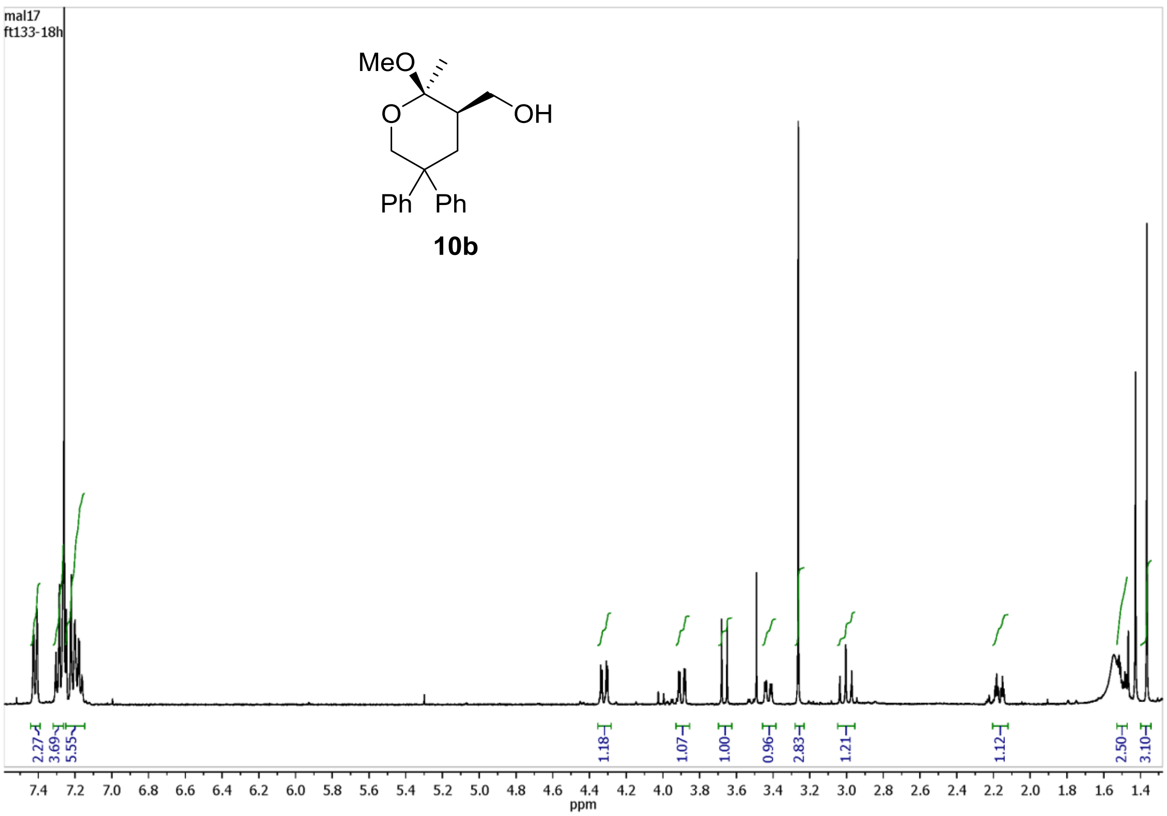
mal17
ft133-18h
MeO
O
OH
Ph
Ph
10b
2.27
3.69
5.55
1.18
1.07
1.00
0.96
2.83
1.21
1.12
2.50
3.10
ppm

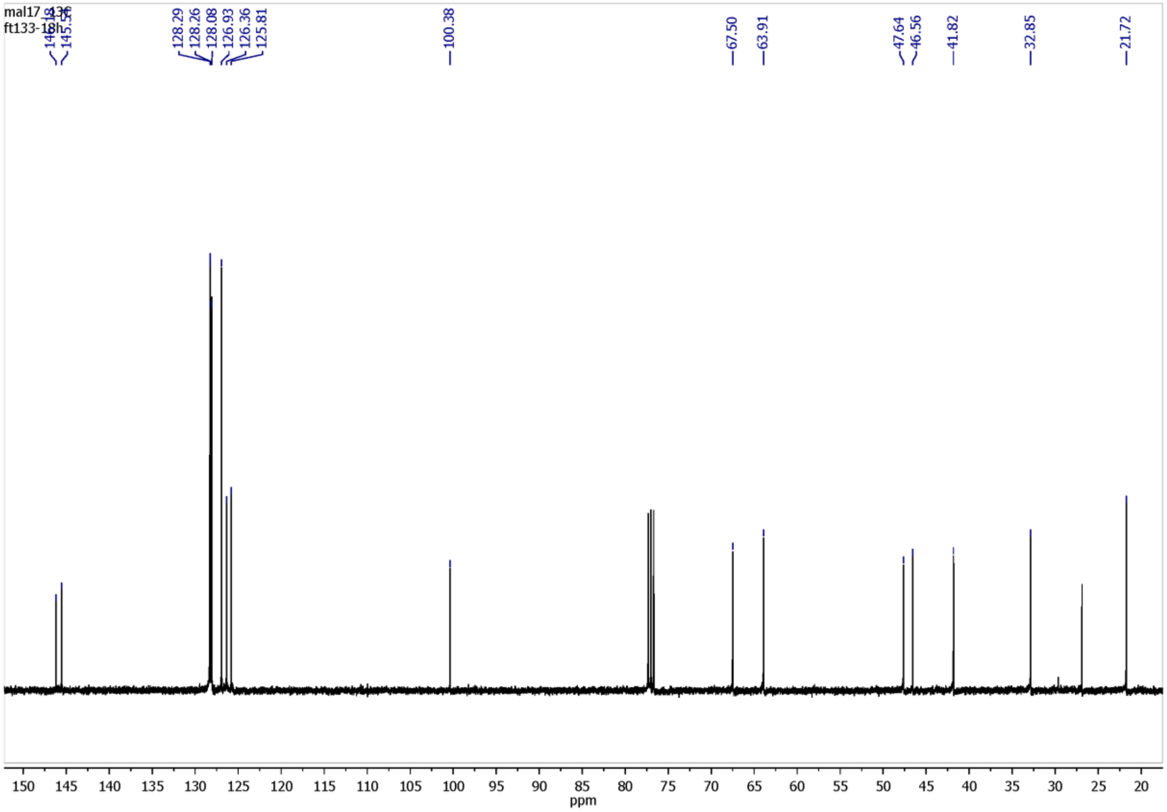
mal17-13C
ft133-18h
128.29
128.26
128.08
126.93
126.36
125.81
100.38
67.50
63.91
47.64
46.56
41.82
32.85
21.72
ppm

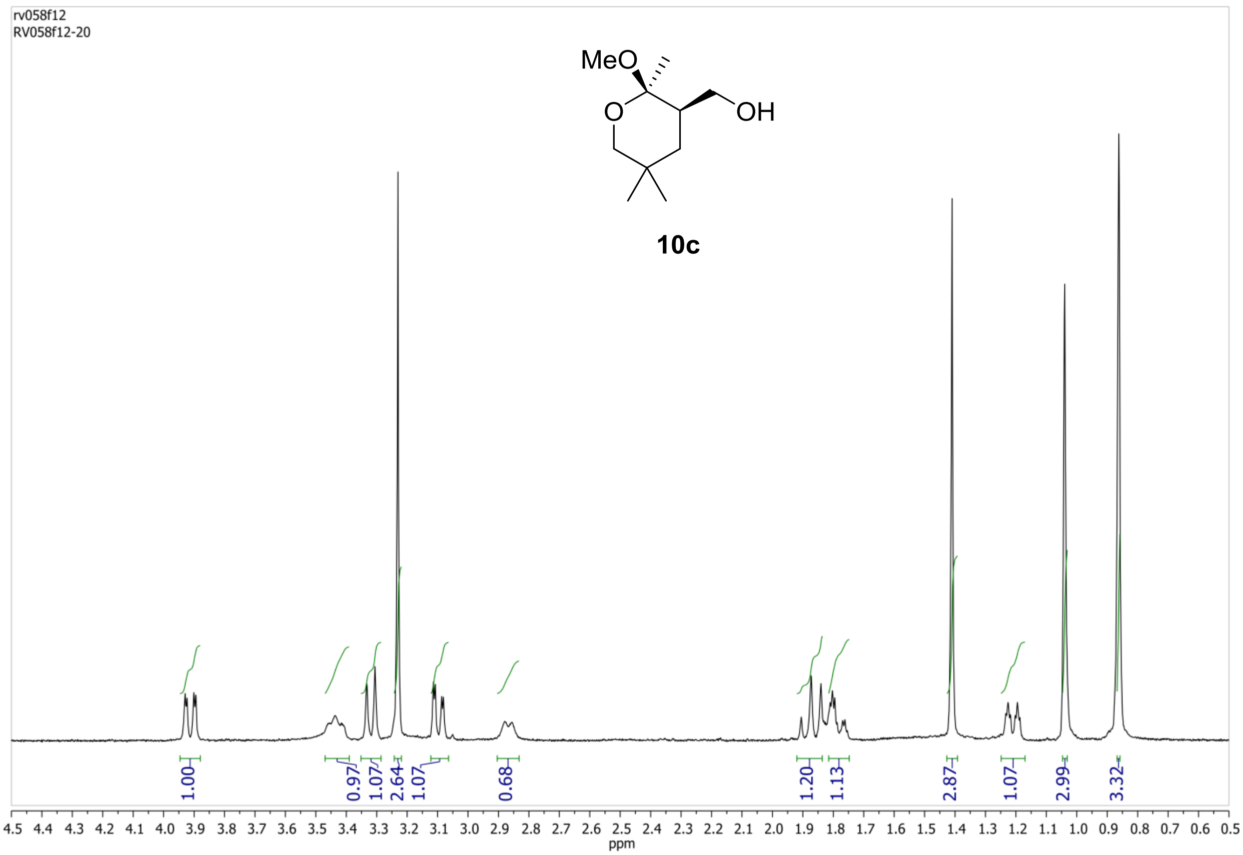
rv058f12
RV058f12-20
MeO
O
OH
10c
1.00
0.97
1.07
2.64
1.07
0.68
1.20
1.13
2.87
1.07
2.99
3.32
4.5 4.4 4.3 4.2 4.1 4.0 3.9 3.8 3.7 3.6 3.5 3.4 3.3 3.2 3.1 3.0 2.9 2.8 2.7 2.6 2.5 2.4 2.3 2.2 2.1 2.0 1.9 1.8 1.7 1.6 1.5 1.4 1.3 1.2 1.1 1.0 0.9 0.8 0.7 0.6 0.5
ppm

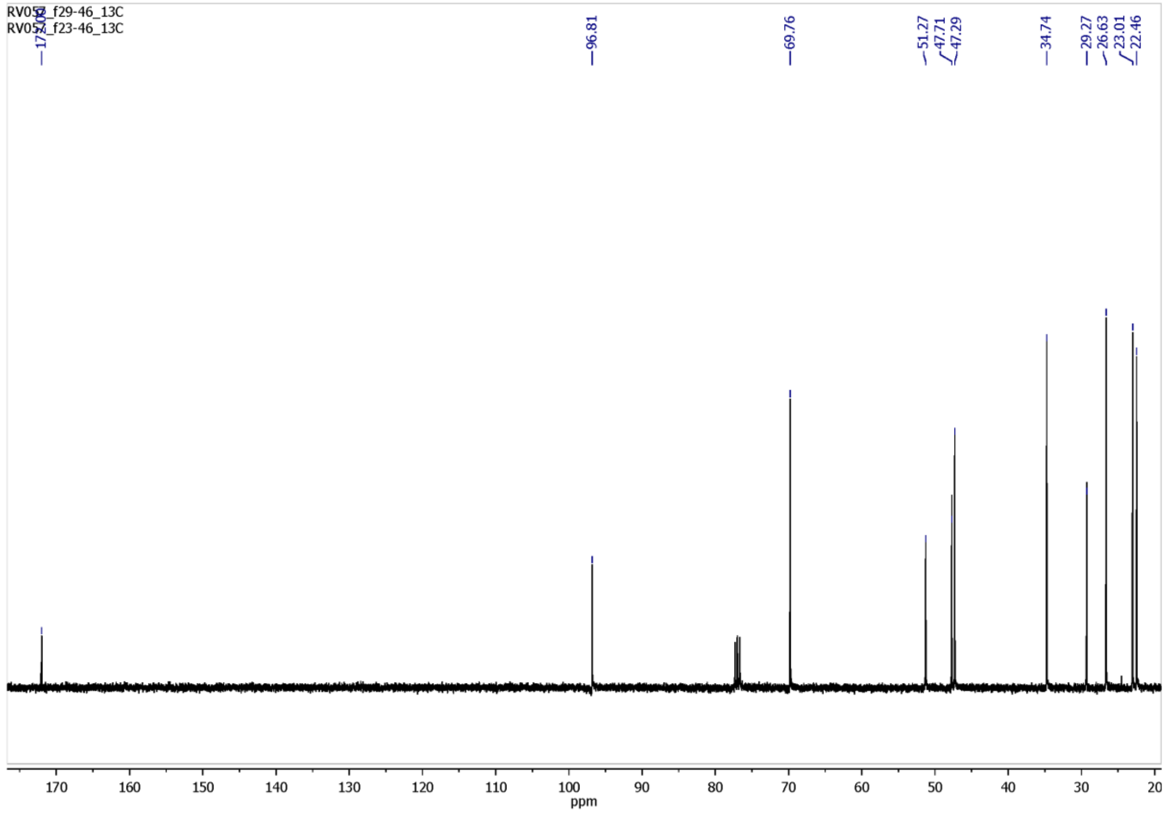
RV05_f29-46_13C
RV05_f23-46_13C
96.81
69.76
51.27
47.71
47.29
34.74
29.27
26.63
23.01
22.46
170 160 150 140 130 120 110 100 90 80 70 60 50 40 30 20
ppm

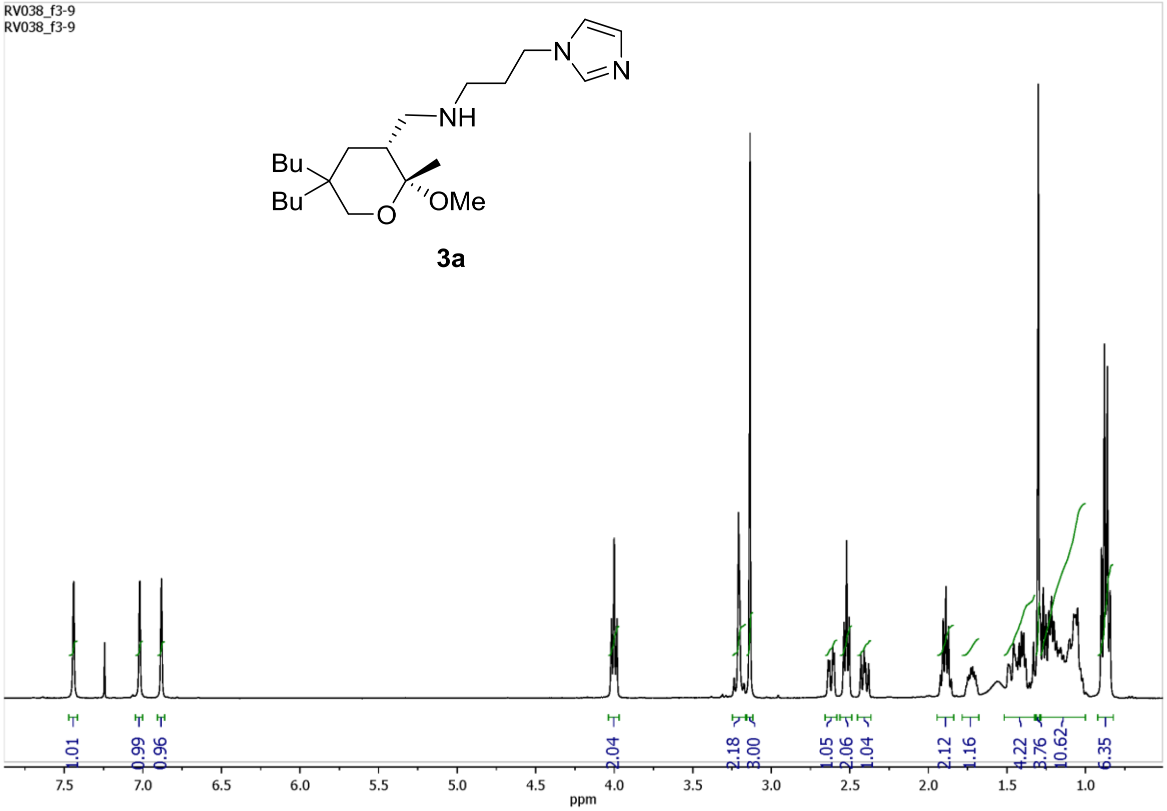
RV038_f3-9
RV038_f3-9
NH
N
N
Bu
Bu
O
OMe
3a
1.01
0.99
0.96
2.04
2.18
3.00
1.05
2.06
1.04
2.12
1.16
4.22
3.76
10.62
6.35
ppm

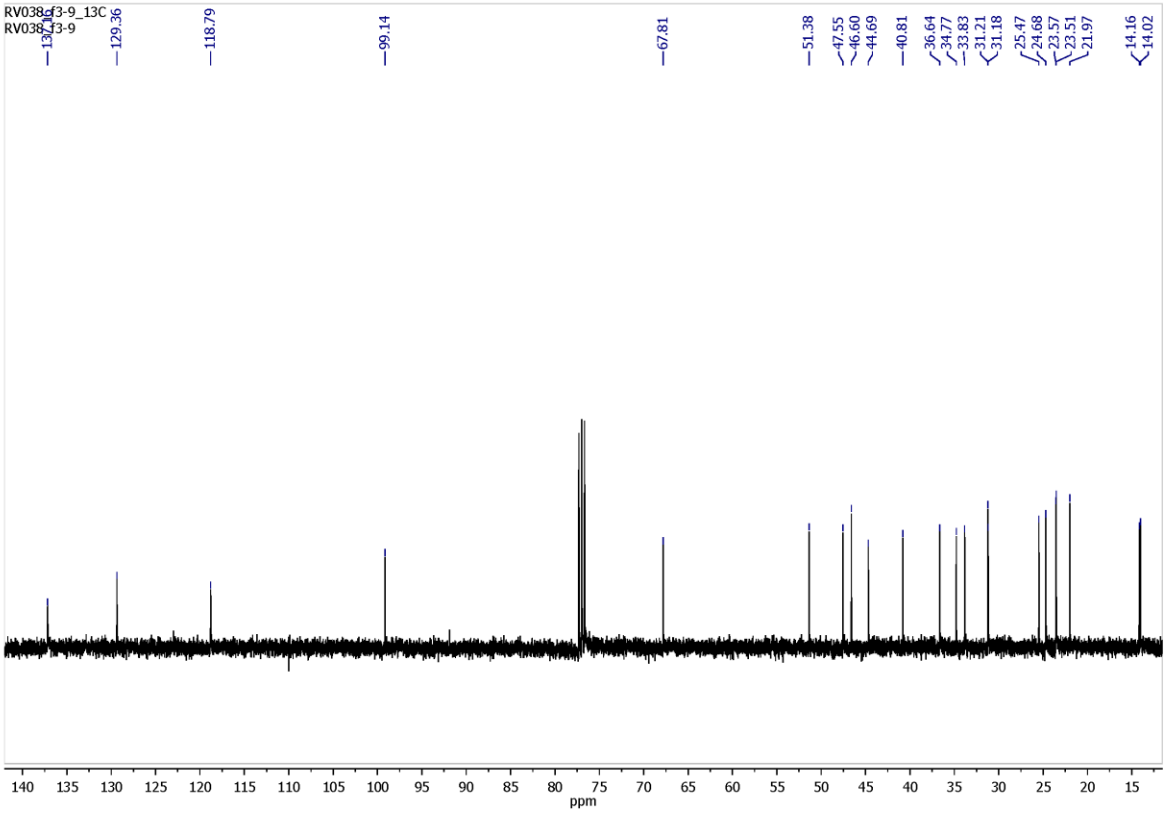
RV038_f3-9_13C
RV038_f3-9
129.36
118.79
99.14
67.81
51.38
47.55
46.60
44.69
40.81
36.64
34.77
33.83
31.21
31.18
25.47
24.68
23.57
23.51
21.97
14.16
14.02
ppm

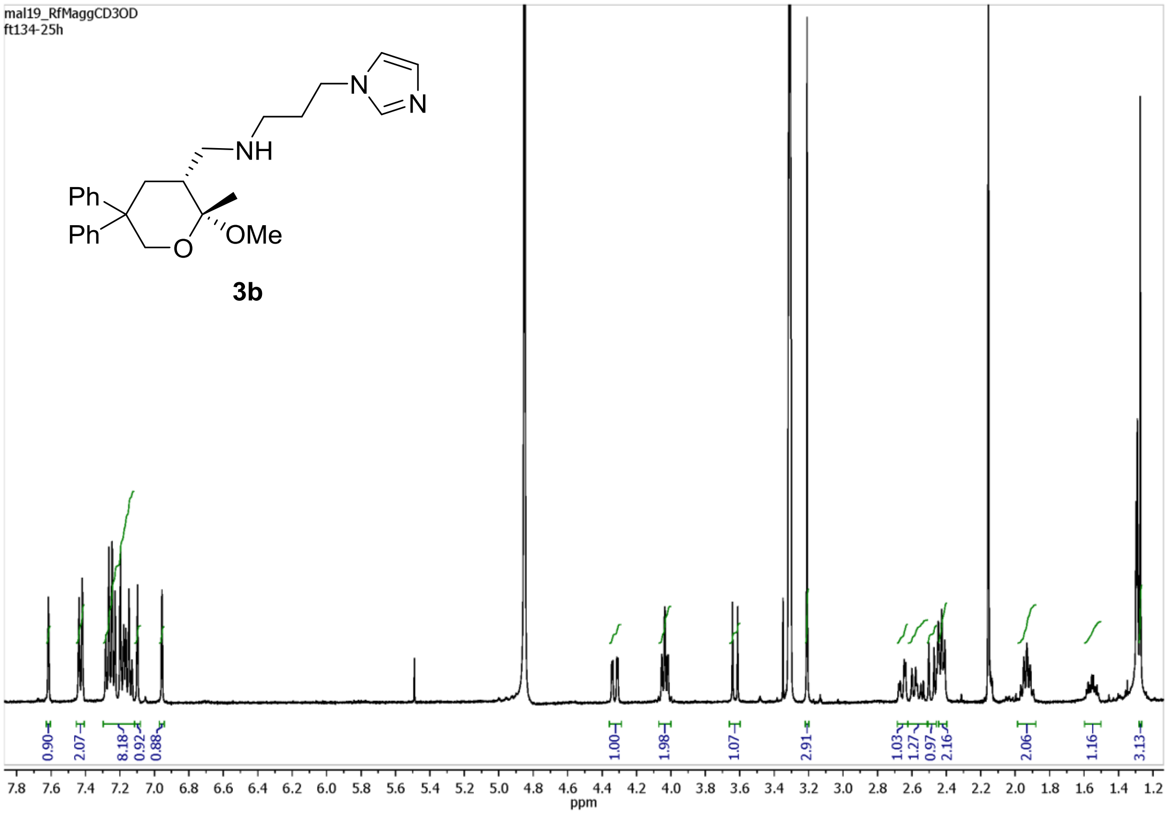
mal19_RfMaggCD3OD
ft134-25h
NH
N
N
Ph
Ph
O
OMe
3b
0.90
2.07
8.18
0.92
0.88
1.00
1.98
1.07
2.91
1.03
1.27
0.97
2.16
2.06
1.16
3.13
ppm

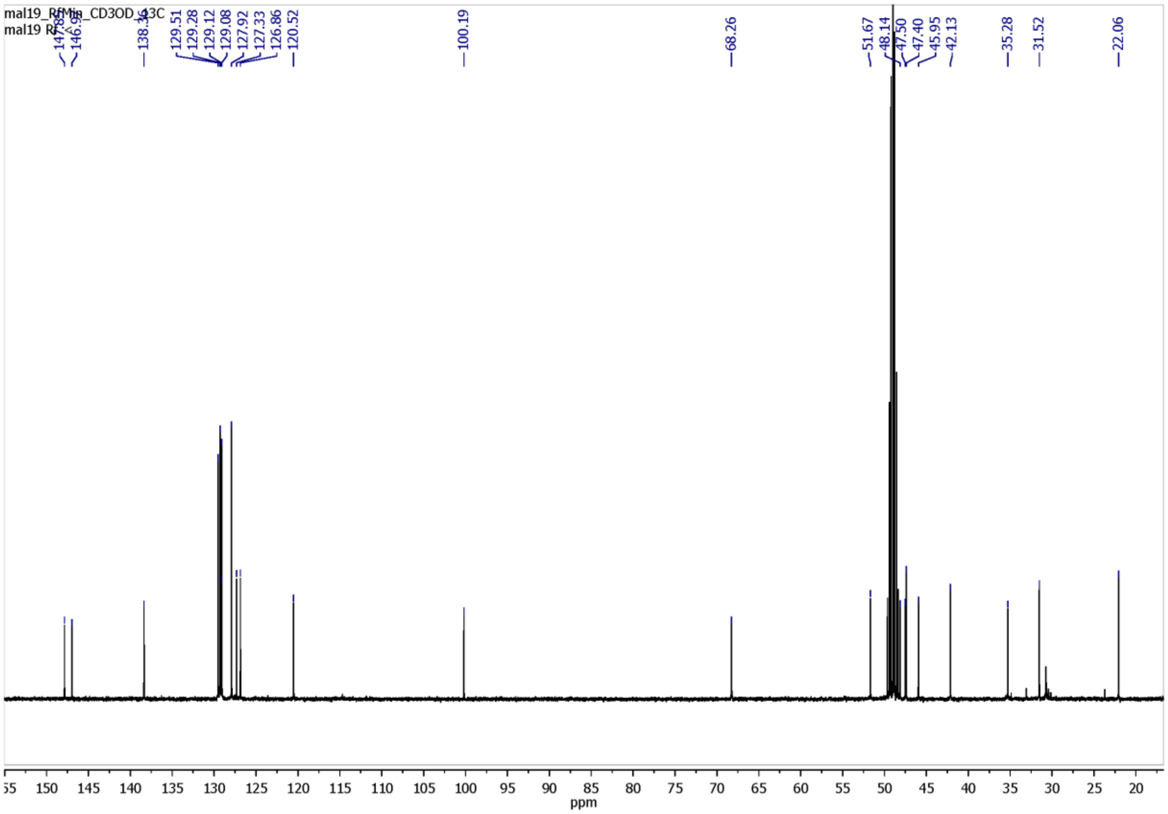
138.3
129.51
129.28
129.12
129.08
127.92
127.33
126.86
120.52
100.19
68.26
51.67
48.14
47.50
47.40
45.95
42.13
35.28
31.52
22.06
ppm

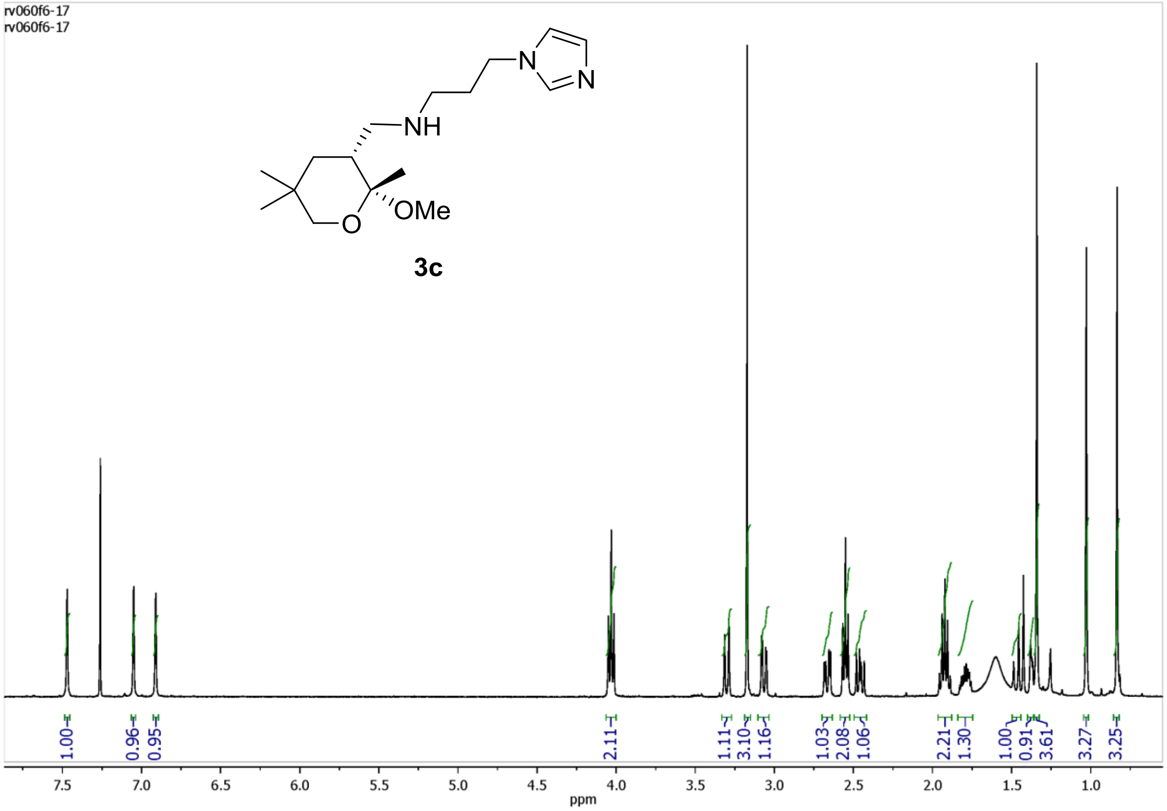
rv060f6-17
rv060f6-17
NH
N
N
OMe
O
3c
1.00
0.96
0.95
2.11
1.11
3.10
1.16
1.03
2.08
1.06
2.21
1.30
1.00
0.91
3.61
3.27
3.25
ppm

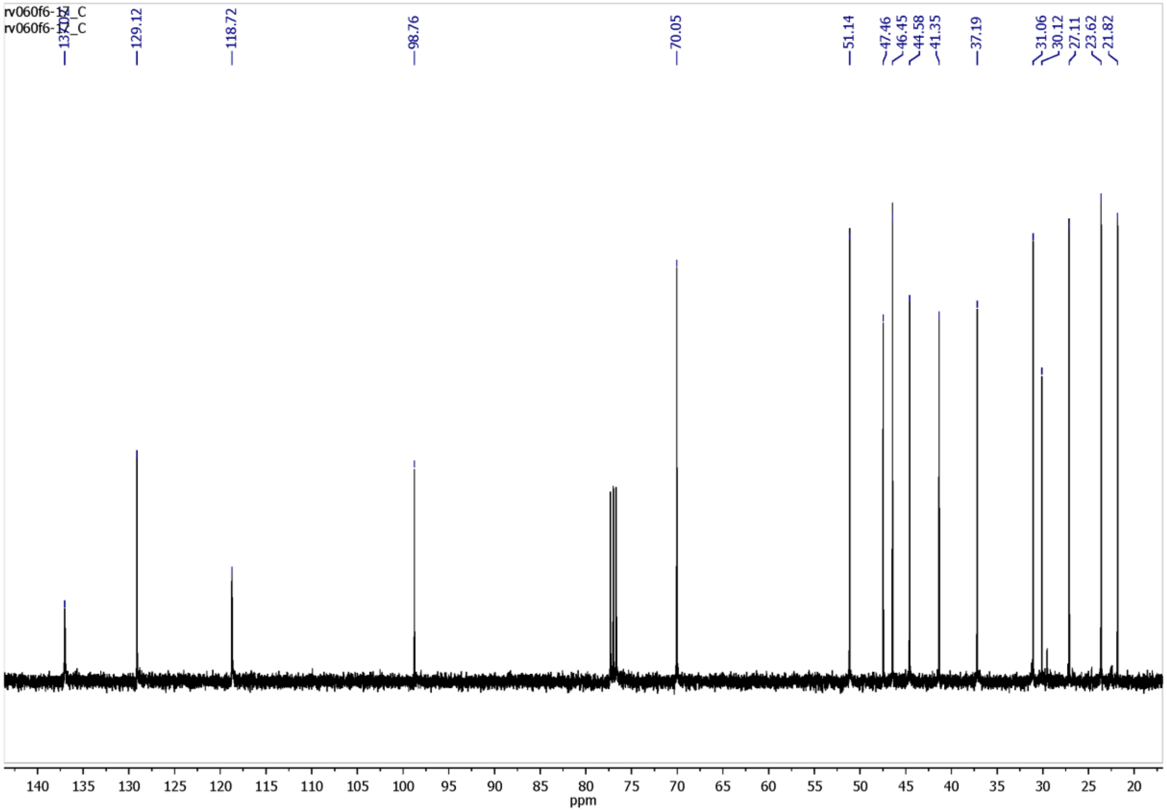
129.12
118.72
98.76
70.05
51.14
47.46
46.45
44.58
41.35
37.19
31.06
30.12
27.11
23.62
21.82
ppm

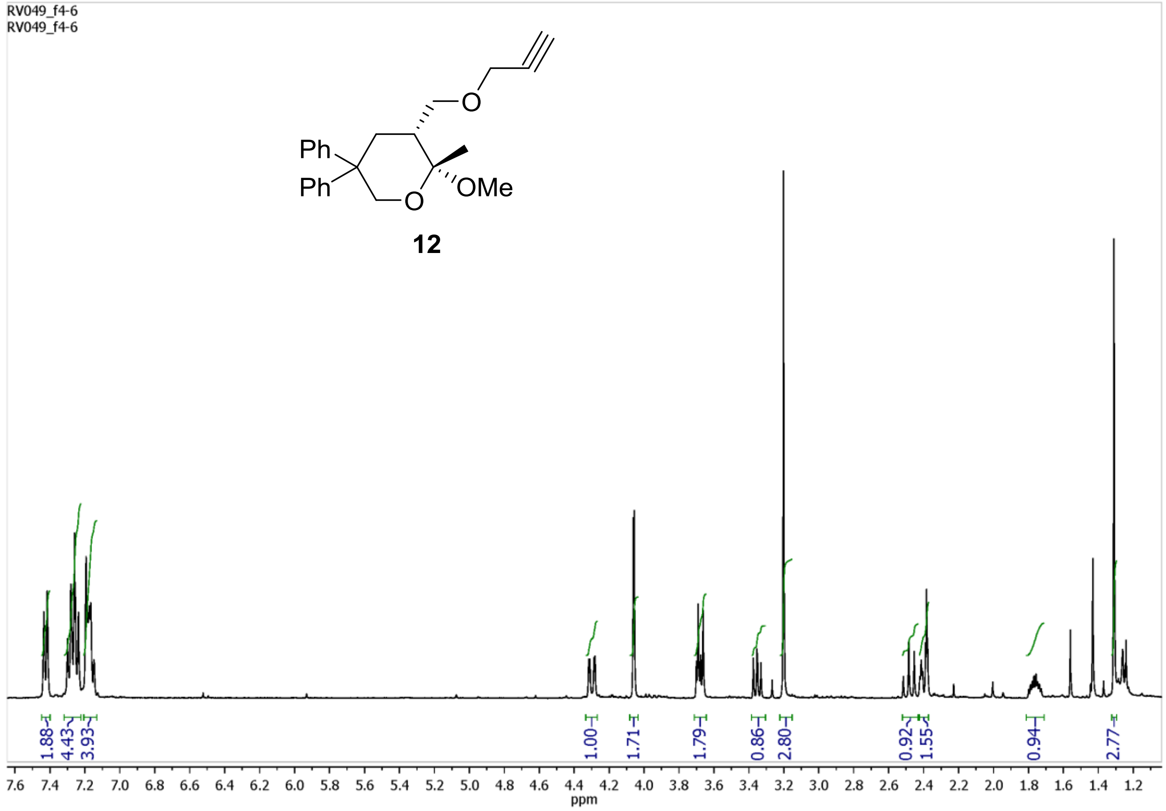
RV049_f4-6
RV049_f4-6
Ph
Ph
O
OMe
O
12
1.88
4.43
3.93
1.00
1.71
1.79
0.86
2.80
0.92
1.55
0.94
2.77
ppm

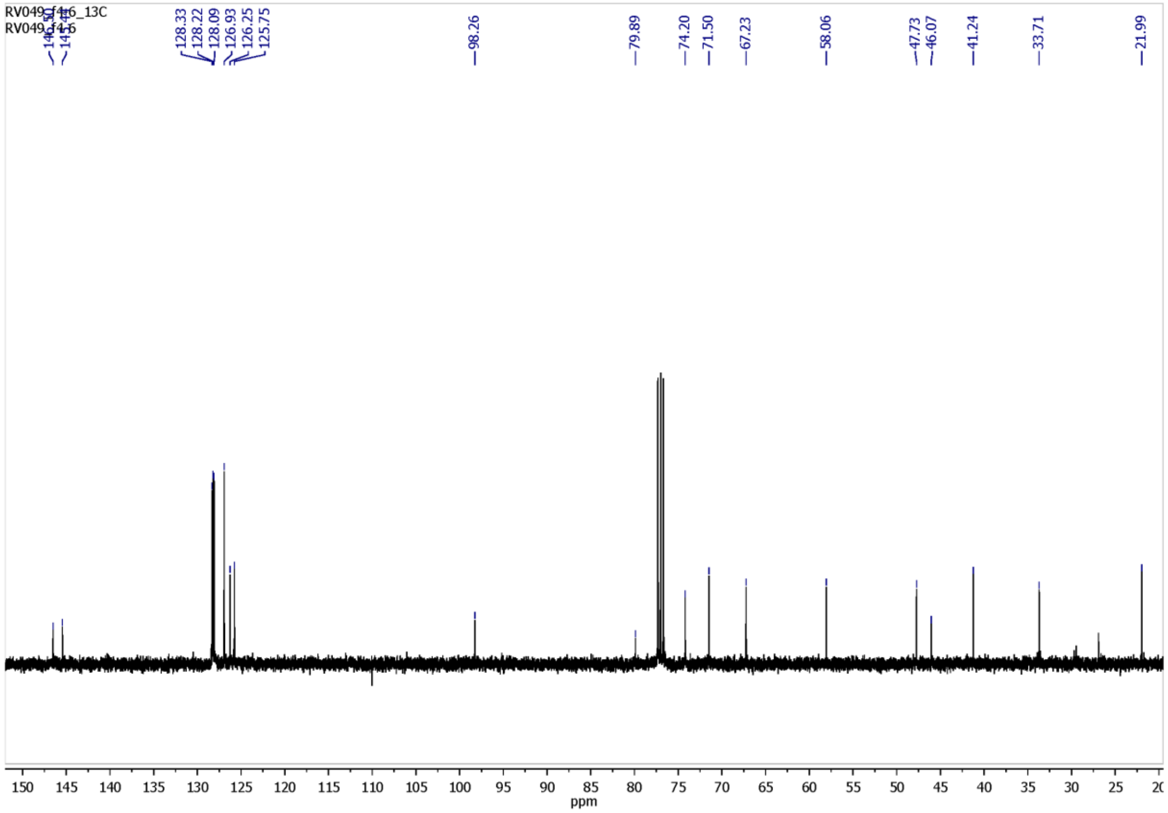
RV049_f4-6_13C
RV049_f4-6
128.33
128.22
128.09
126.93
126.25
125.75
98.26
79.89
74.20
71.50
67.23
58.06
47.73
46.07
41.24
33.71
21.99
ppm

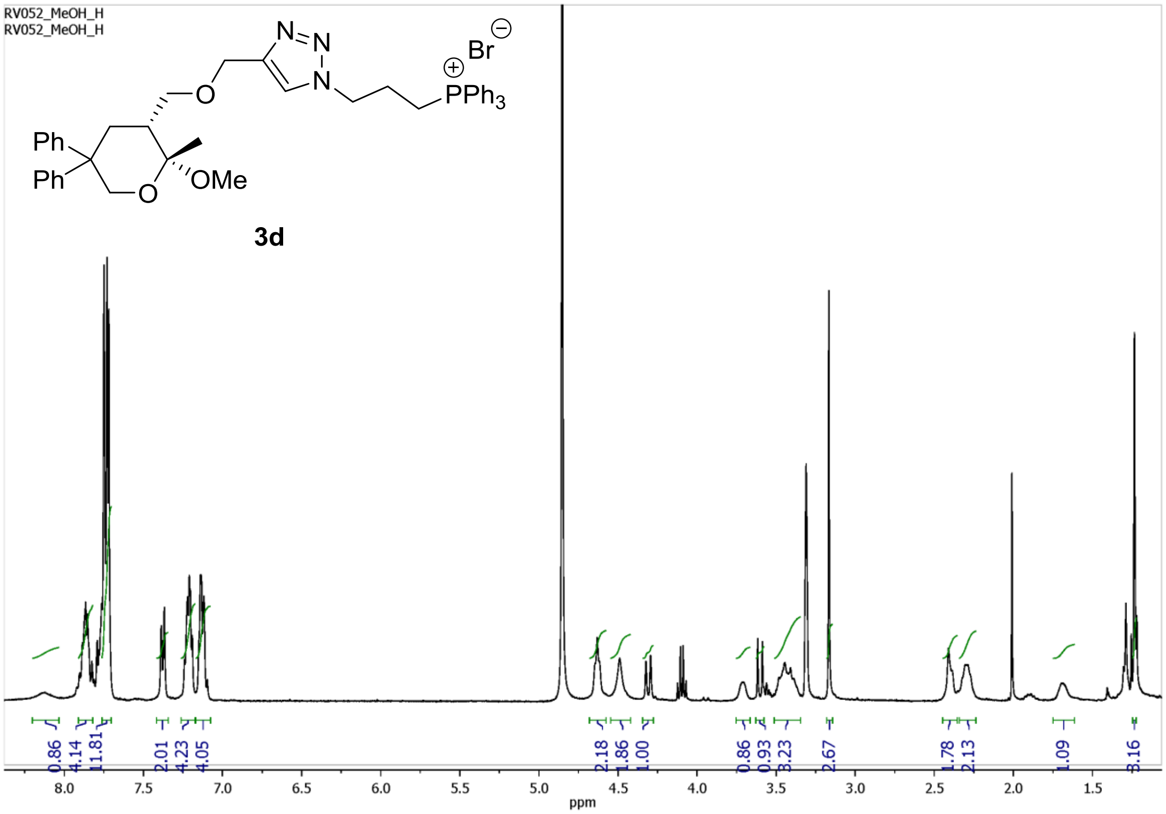
RV052_MeOH_H
3d
ppm

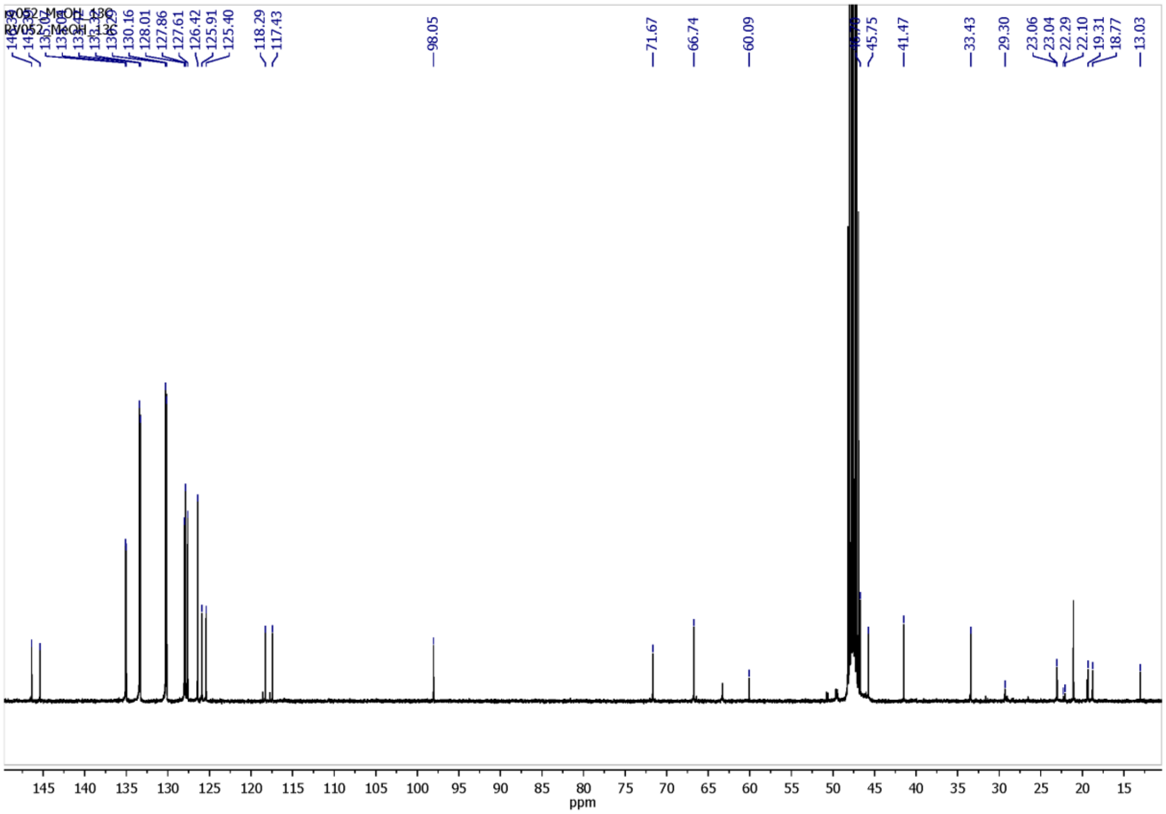
ppm

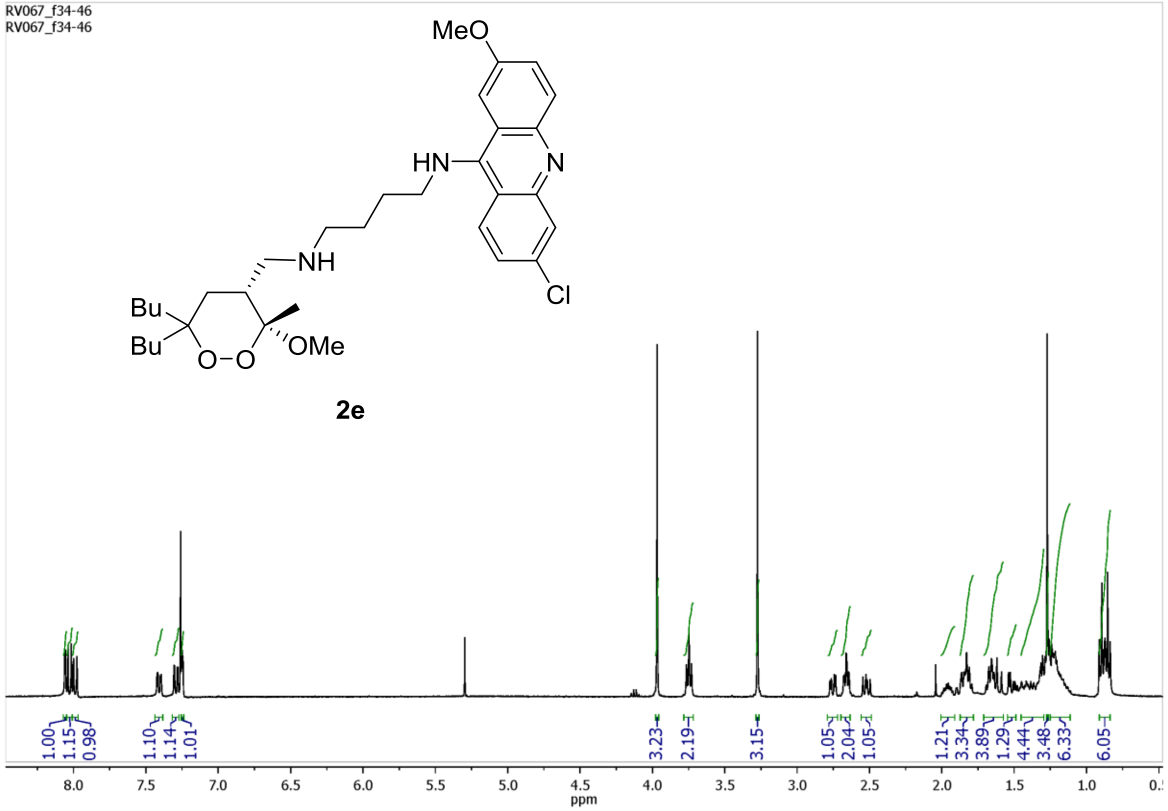
RV067_f34-46
RV067_f34-46
MeO
HN
N
NH
Cl
Bu
Bu
O–O
OMe
2e
1.00
1.15
0.98
1.10
1.14
1.01
3.23
2.19
3.15
1.05
2.04
1.05
1.21
3.34
3.89
1.29
4.44
3.48
6.33
6.05
ppm

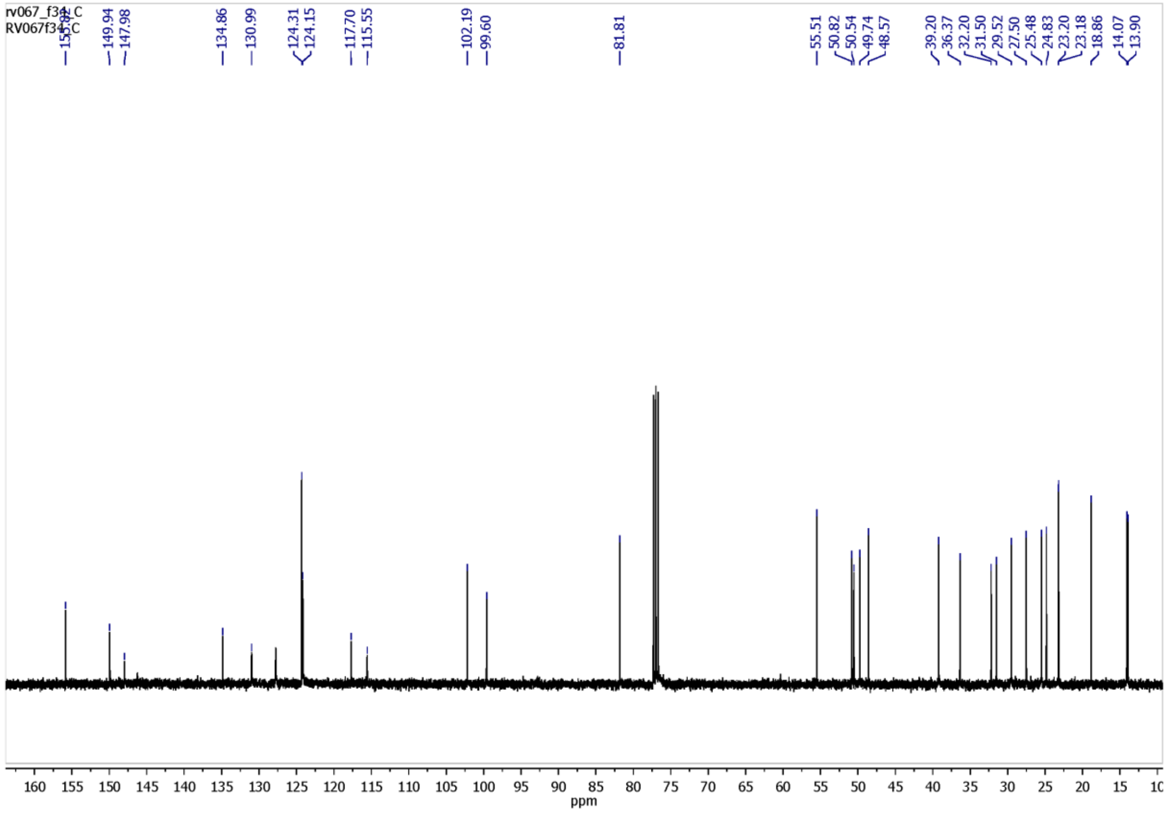
rv067_f34_C
RV067f34_C
149.94
147.98
134.86
130.99
124.31
124.15
117.70
115.55
102.19
99.60
81.81
55.51
50.82
50.54
49.74
48.57
39.20
36.37
32.20
31.50
29.52
27.50
25.48
24.83
23.20
23.18
18.86
14.07
13.90
ppm

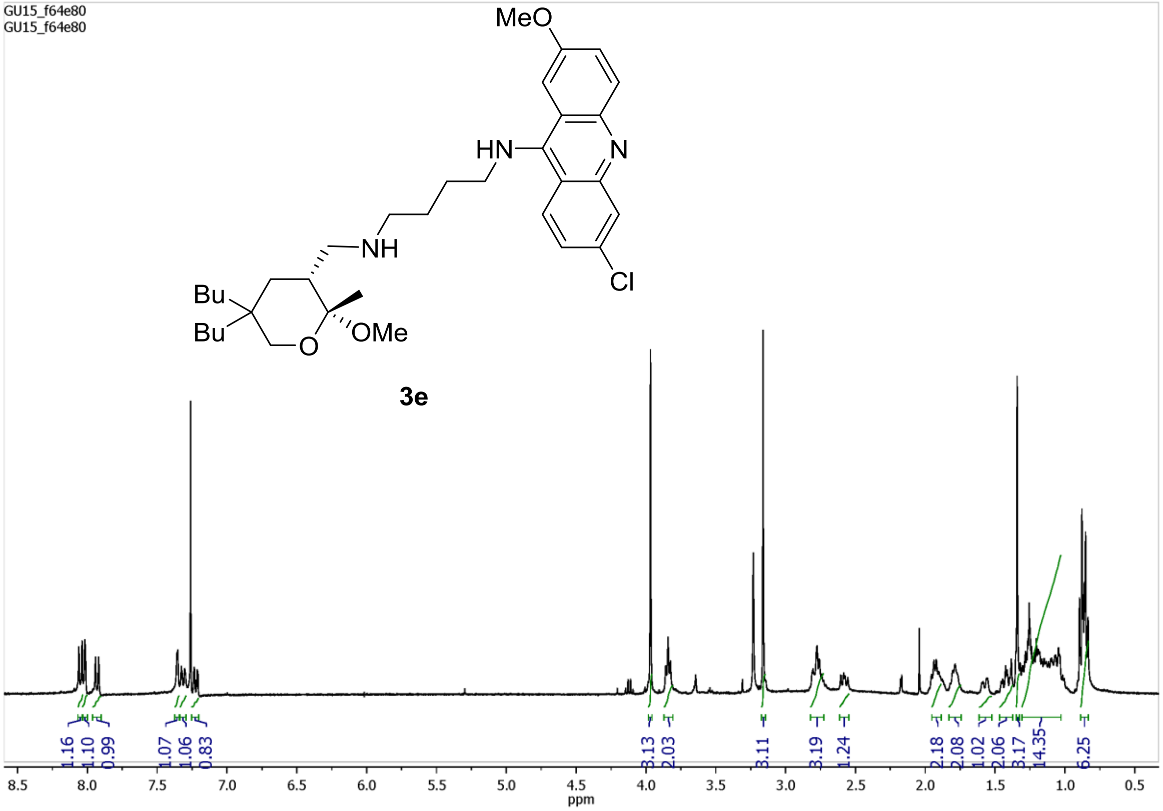
GU15_f64e80
GU15_f64e80
MeO
HN
N
NH
Cl
Bu
Bu
O
OMe
3e
1.16
1.10
0.99
1.07
1.06
0.83
3.13
2.03
3.11
3.19
1.24
2.18
2.08
1.02
2.06
3.17
14.35
6.25
8.5
8.0
7.5
7.0
6.5
6.0
5.5
5.0
4.5
4.0
3.5
3.0
2.5
2.0
1.5
1.0
0.5
ppm

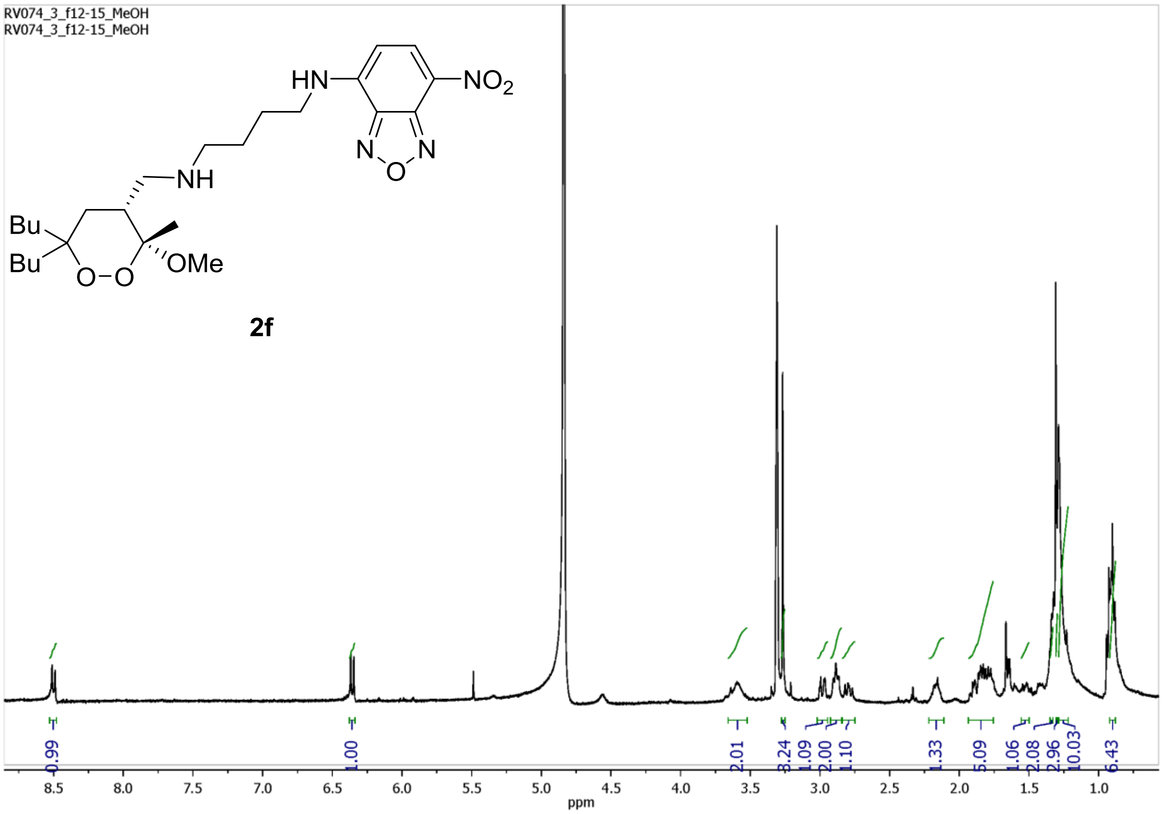
RV074_3_f12-15_MeOH
RV074_3_f12-15_MeOH
HN
NO2
N
O
N
NH
Bu
Bu
O–O
OMe
2f
0.99
1.00
2.01
3.24
1.09
2.00
1.10
1.33
5.09
1.06
2.08
2.96
10.03
6.43
8.5
8.0
7.5
7.0
6.5
6.0
5.5
5.0
4.5
4.0
3.5
3.0
2.5
2.0
1.5
1.0
ppm

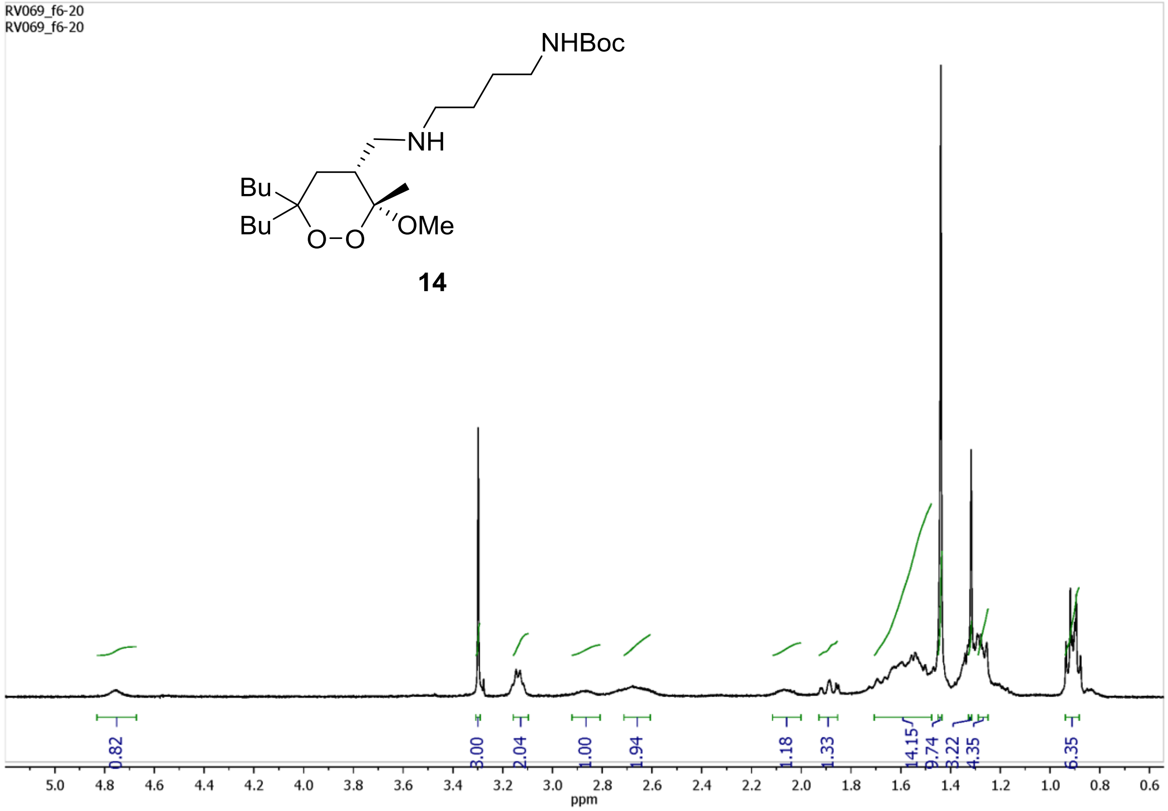
RV069_f6-20
RV069_f6-20
NHBoc
NH
Bu
Bu
O–O
OMe
14
0.82
3.00
2.04
1.00
1.94
1.18
1.33
14.15
9.74
3.22
4.35
6.35
ppm

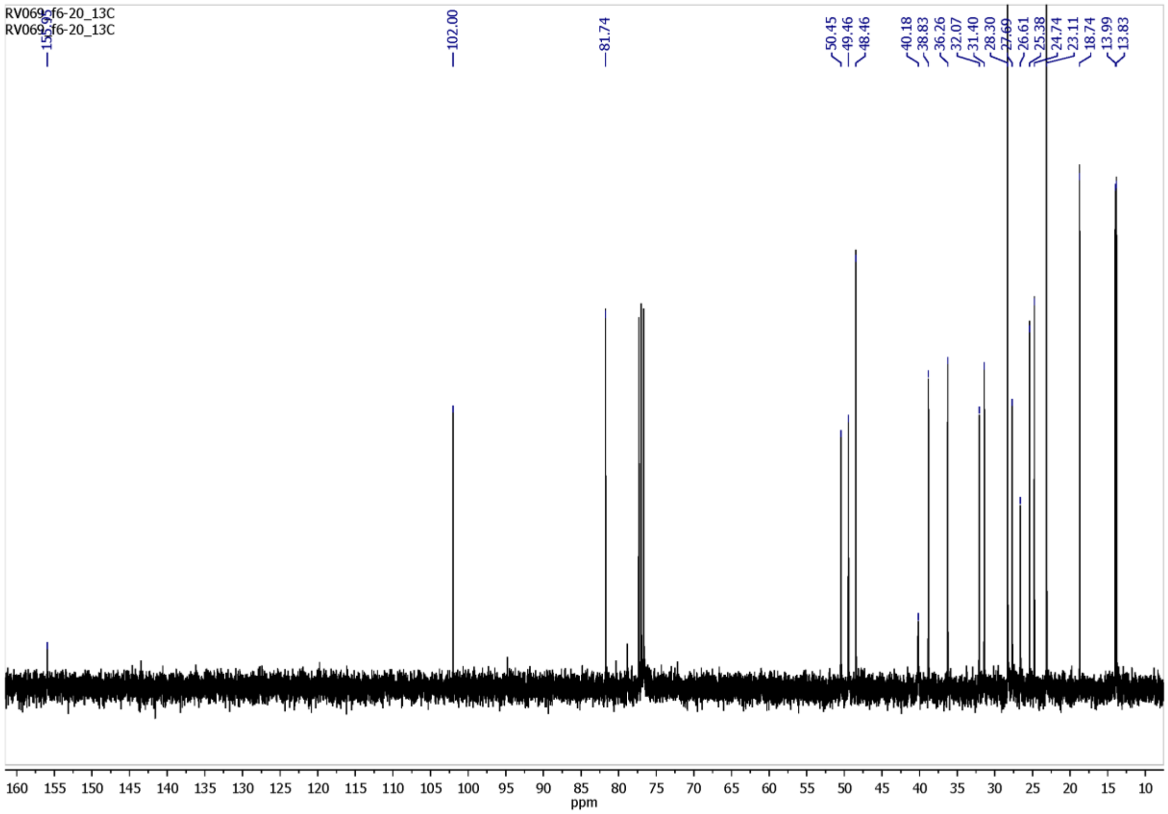
RV069_f6-20_13C
RV069_f6-20_13C
102.00
81.74
50.45
49.46
48.46
40.18
38.83
36.26
32.07
31.40
28.30
27.69
26.61
25.38
24.74
23.11
18.74
13.99
13.83
ppm

HPLC analyses

Compound **3a** (*method B*):

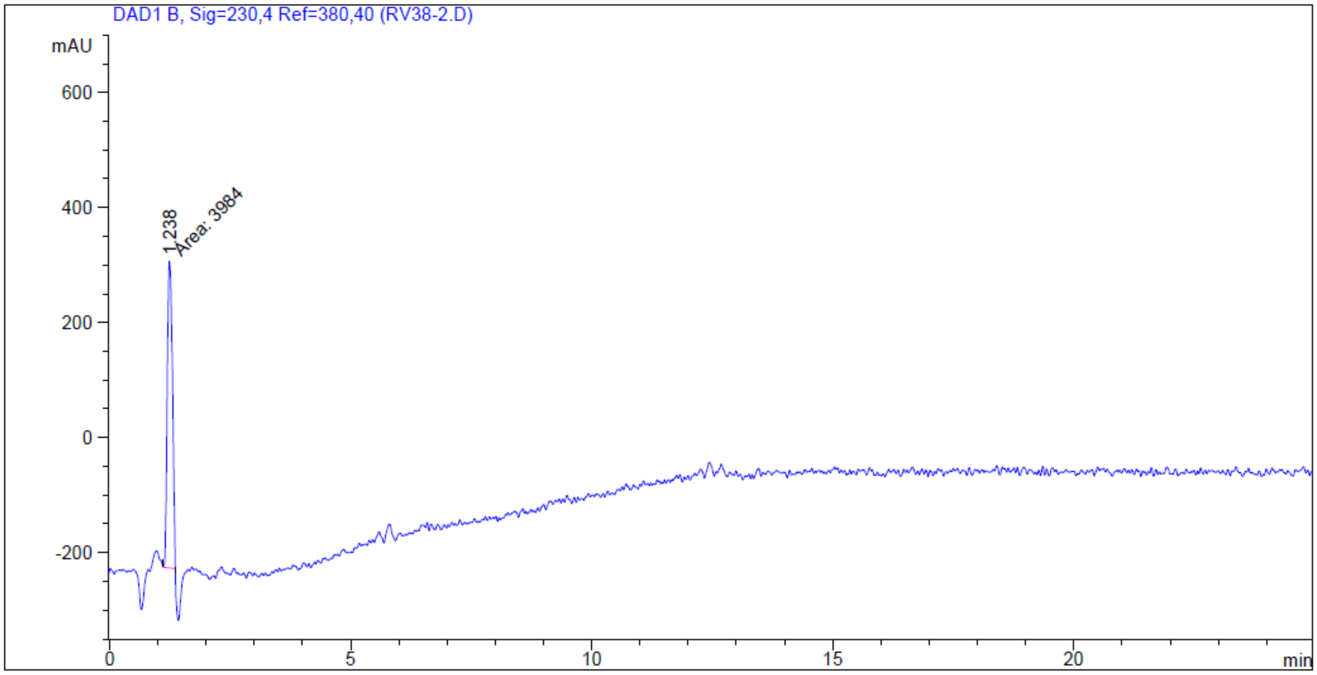


```
=====================================================================
                         Area Percent Report
=====================================================================

Sorted By             :      Signal
Multiplier            :      1.0000
Dilution              :      1.0000
Use Multiplier & Dilution Factor with ISTDs


Signal 1: DAD1 B, Sig=230,4 Ref=380,40

Peak RetTime Type  Width     Area      Height     Area
  #    [min]        [min]   [mAU*s]     [mAU]      %
----|-------|----|-------|----------|----------|--------|
   1   1.238 MM    0.1240 3983.99561  535.34558 100.0000
```

Compound **3b** (*method A*):

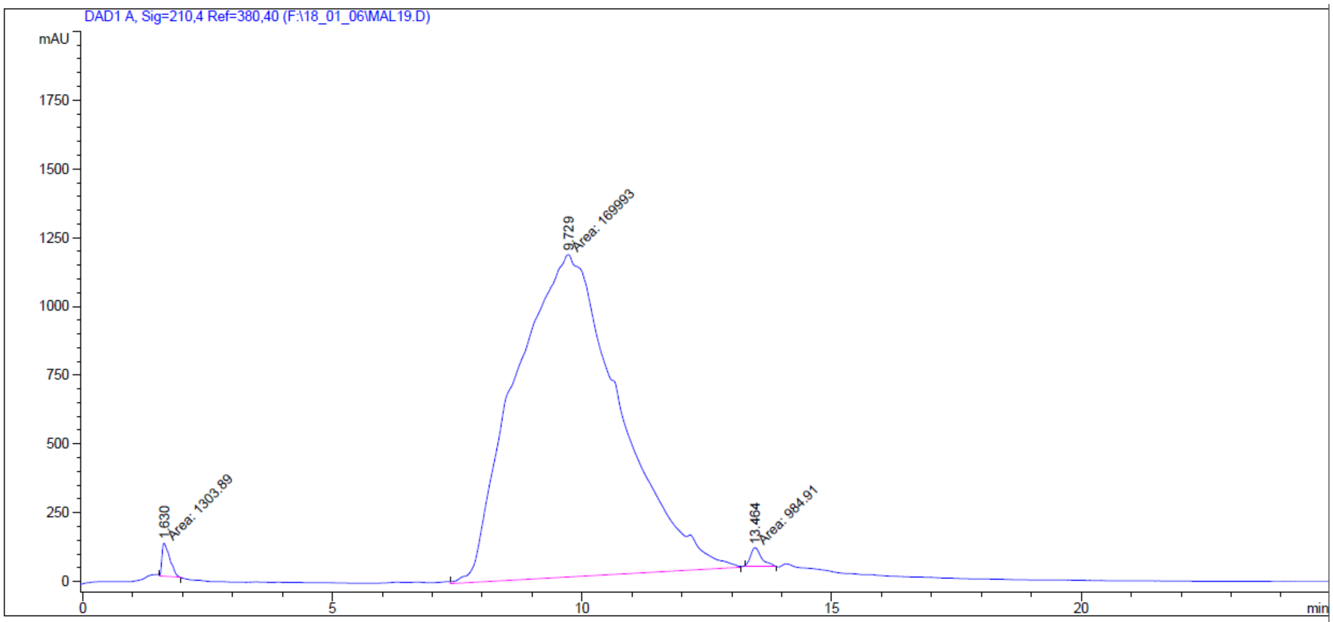


```
=====================================================================
                         Area Percent Report
=====================================================================

Sorted By             :      Signal
Multiplier            :      1.0000
Dilution              :      1.0000
Use Multiplier & Dilution Factor with ISTDs


Signal 1: DAD1 A, Sig=210,4 Ref=380,40

Peak RetTime Type  Width     Area      Height     Area
  #   [min]        [min]   [mAU*s]     [mAU]        %
----|-------|----|-------|----------|----------|--------|
   1   1.630 MM    0.1802 1303.88806  120.57082   0.7568
   2   9.729 MM    2.4164 1.69993e5  1172.46887  98.6715
   3  13.464 MM    0.2480  984.91028   66.19936   0.5717
```

Compound **3c** (*method B*):

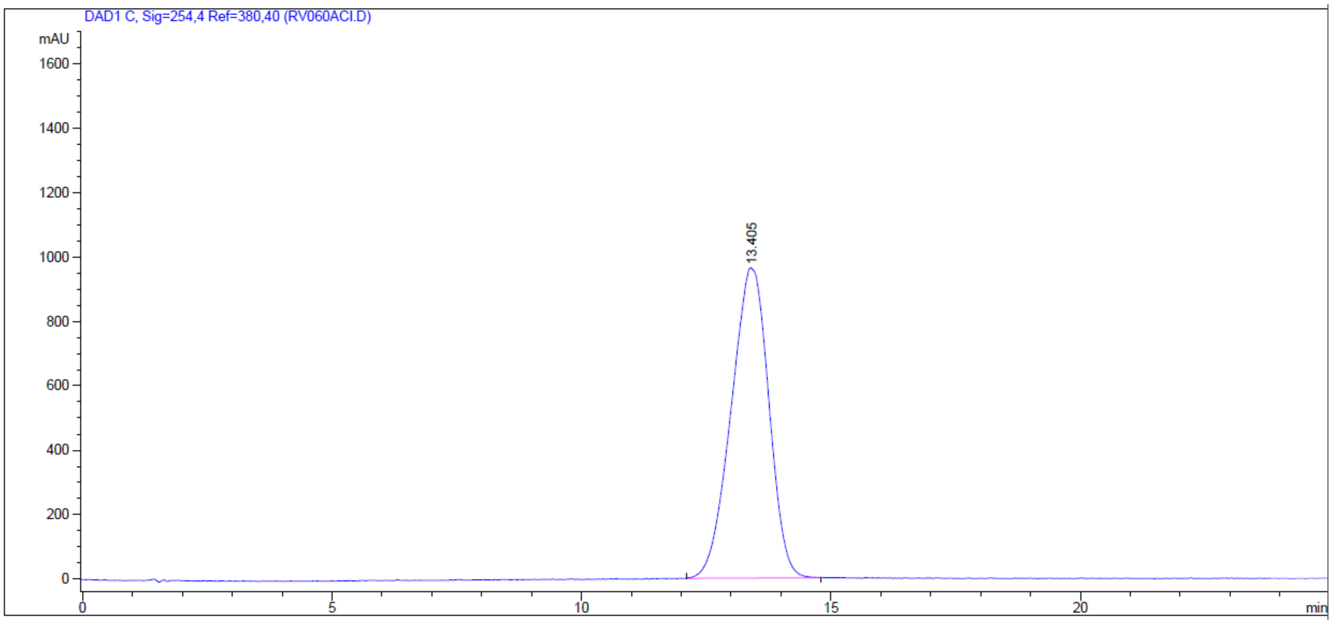


```
=====================================================================
                       Area Percent Report
=====================================================================

Sorted By             :      Signal
Multiplier            :      1.0000
Dilution              :      1.0000
Use Multiplier & Dilution Factor with ISTDs


Signal 1: DAD1 C, Sig=254,4 Ref=380,40

Peak RetTime Type  Width     Area      Height     Area
  #   [min]        [min]   [mAU*s]     [mAU]        %
----|-------|----|-------|----------|----------|--------|
   1  13.405 BB    0.6263 5.05425e4  963.09241 100.0000
```

Compound **3d** (*method B*):

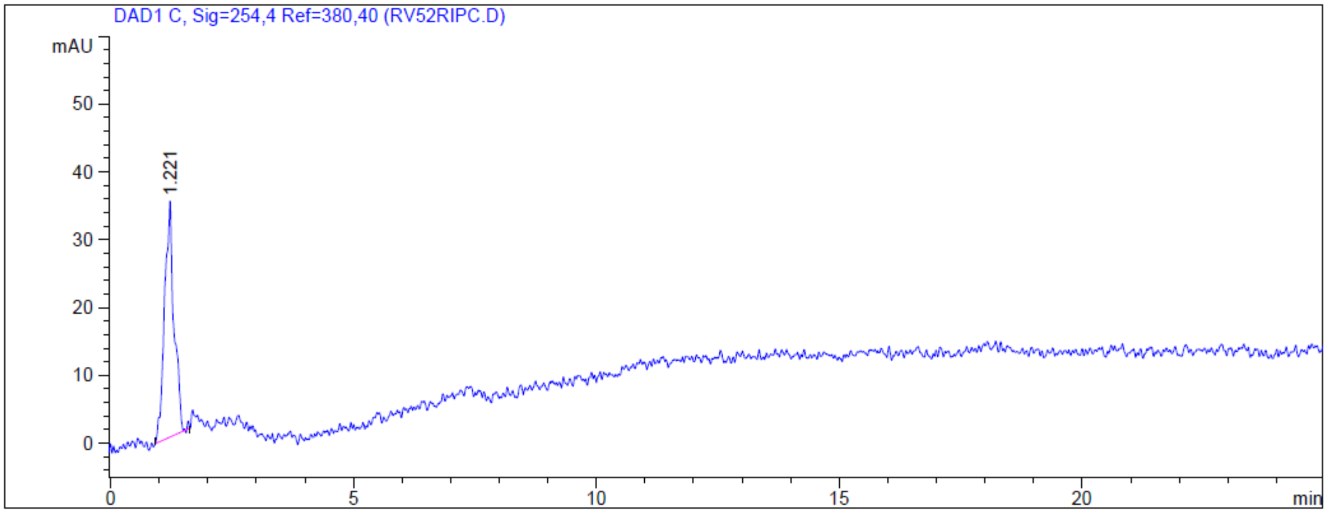


```
==================================================================
                         Area Percent Report
==================================================================

Sorted By             :      Signal
Multiplier            :      1.0000
Dilution              :      1.0000
Use Multiplier & Dilution Factor with ISTDs


Signal 1: DAD1 C, Sig=254,4 Ref=380,40

Peak RetTime Type  Width     Area      Height     Area
  #   [min]        [min]   [mAU*s]     [mAU]        %
----|-------|----|-------|----------|----------|--------|
   1   1.221 PB    0.1721  472.37149   34.84964 100.0000
```

Compound **3e** (*method A*):

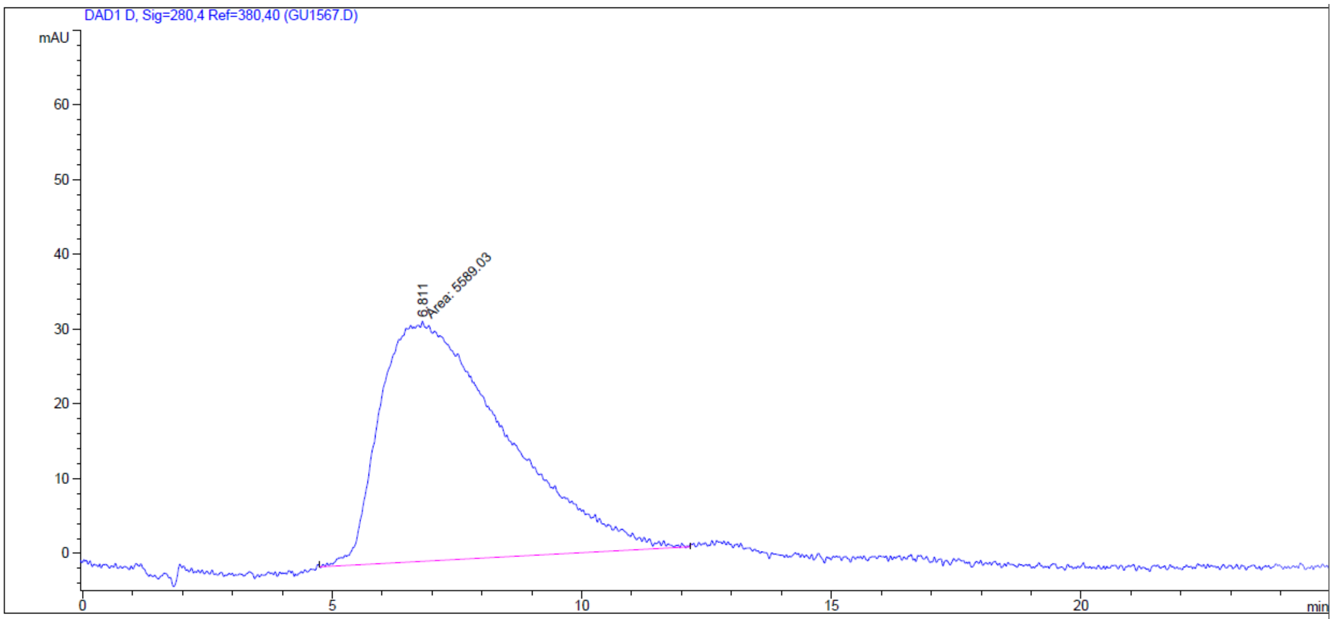


```
=====================================================================
                          Area Percent Report
=====================================================================

Sorted By             :      Signal
Multiplier            :      1.0000
Dilution              :      1.0000
Use Multiplier & Dilution Factor with ISTDs


Signal 1: DAD1 D, Sig=280,4 Ref=380,40

Peak RetTime Type  Width     Area      Height     Area
  #   [min]        [min]   [mAU*s]     [mAU]        %
----|-------|----|-------|----------|----------|--------|
   1   6.811 MM    2.8949 5589.02539   32.17752 100.0000
```

Compound **2e** (*method B*):

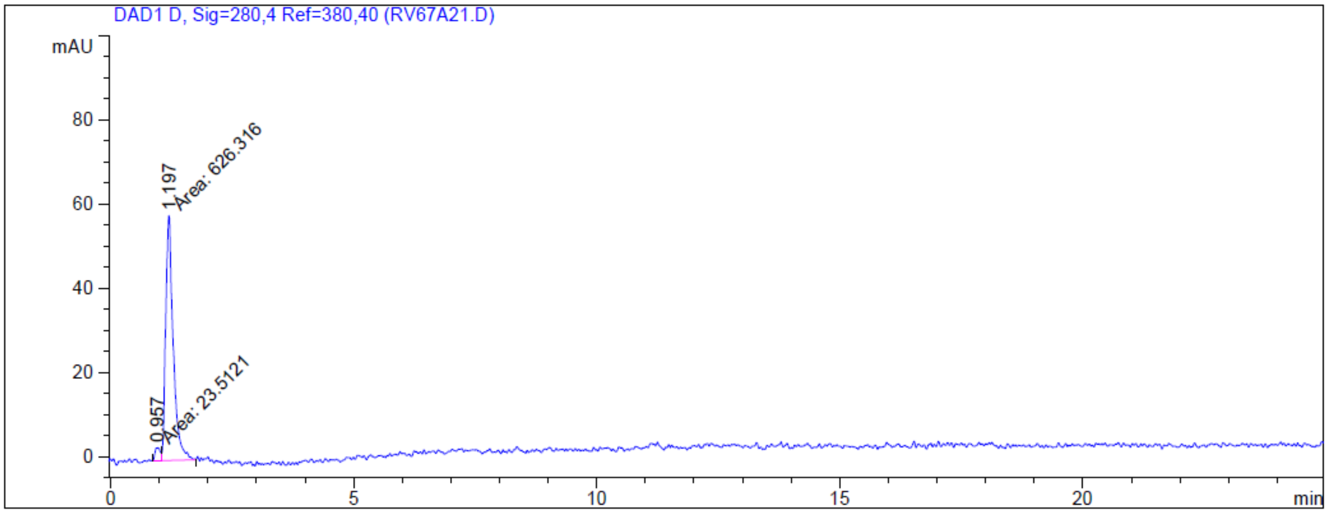

===

Area Percent Report

===

Sorted By : Signal
Multiplier : 1.0000
Dilution : 1.0000
Use Multiplier & Dilution Factor with ISTDs

Signal 1: DAD1 D, Sig=280,4 Ref=380,40

Peak #	RetTime [min]	Type	Width [min]	Area [mAU*s]	Height [mAU]	Area %
1	0.957	MF	0.1273	23.51208	3.07771	3.6182
2	1.197	FM	0.1788	626.31610	58.39663	96.3818

Compound **2f** (*method B*):

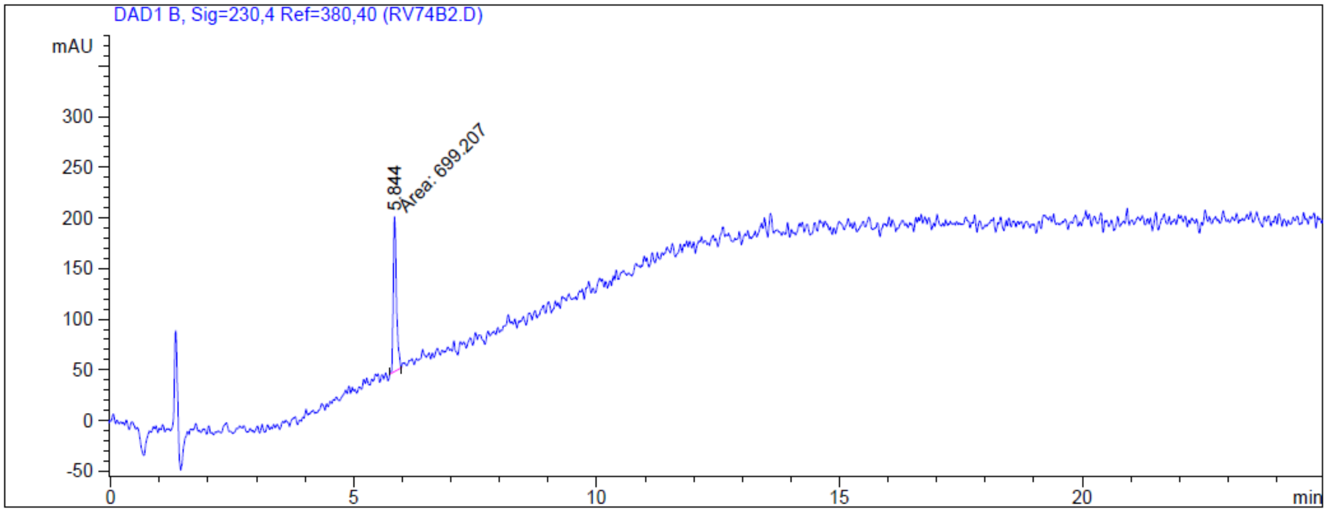


```
=====================================================================
                       Area Percent Report
=====================================================================

Sorted By            :      Signal
Multiplier           :      1.0000
Dilution             :      1.0000
Use Multiplier & Dilution Factor with ISTDs


Signal 1: DAD1 B, Sig=230,4 Ref=380,40

Peak RetTime Type  Width     Area      Height     Area
  #    [min]        [min]    [mAU*s]     [mAU]       %
----|-------|----|-------|----------|----------|--------|
   1   5.844 MM    0.0761  699.20667  153.15910 100.0000
```

References

(1) SMART & SAINT Software Reference Manuals, version 5.051 (Windows NT Version); Bruker Analytical X-ray Instruments Inc.: Madison, WI, **1998**.

(2) Sheldrick, G. M. *SADABS-2008/1 - Bruker AXS Area Detector Scaling and Absorption Correction*, Bruker AXS: Madison, Wisconsin, USA, 2008.

(3) Burla, M. C.; Caliandro, R.; Carrozzini, B.; Cascarano, G. L.; Cuocci, C.; Giacovazzo, C.; Mallamo, M.; Mazzone, A.; Polidori, G. *J. Appl. Cryst.* **2015**, *48*, 306-309.

(4) Sheldrick, G. M. *Acta Cryst C71***,** 2015, 3-8.

(5) Macrae, C. F.; Bruno, I. J.; Chisholm, J. A.; Edgington, P. R.; McCabe, P.; Pidcock, E.; Rodriguez-Monge, L.; Taylor, R.; van de Streek, J.; Wood, P. A. *J. Appl. Cryst.*, **2008**, *41*, 466-470.
